# Supplementary figures and images for: Distribution of myogenic stem cell activator, hepatocyte growth factor, in skeletal muscle extracellular matrix and effect of short-term disuse and reloading
Source: PLoS One. 2025 Sep 3;20(9):e0321839. doi: 10.1371/journal.pone.0321839 (PMC12407438; doi:10.1371/journal.pone.0321839)

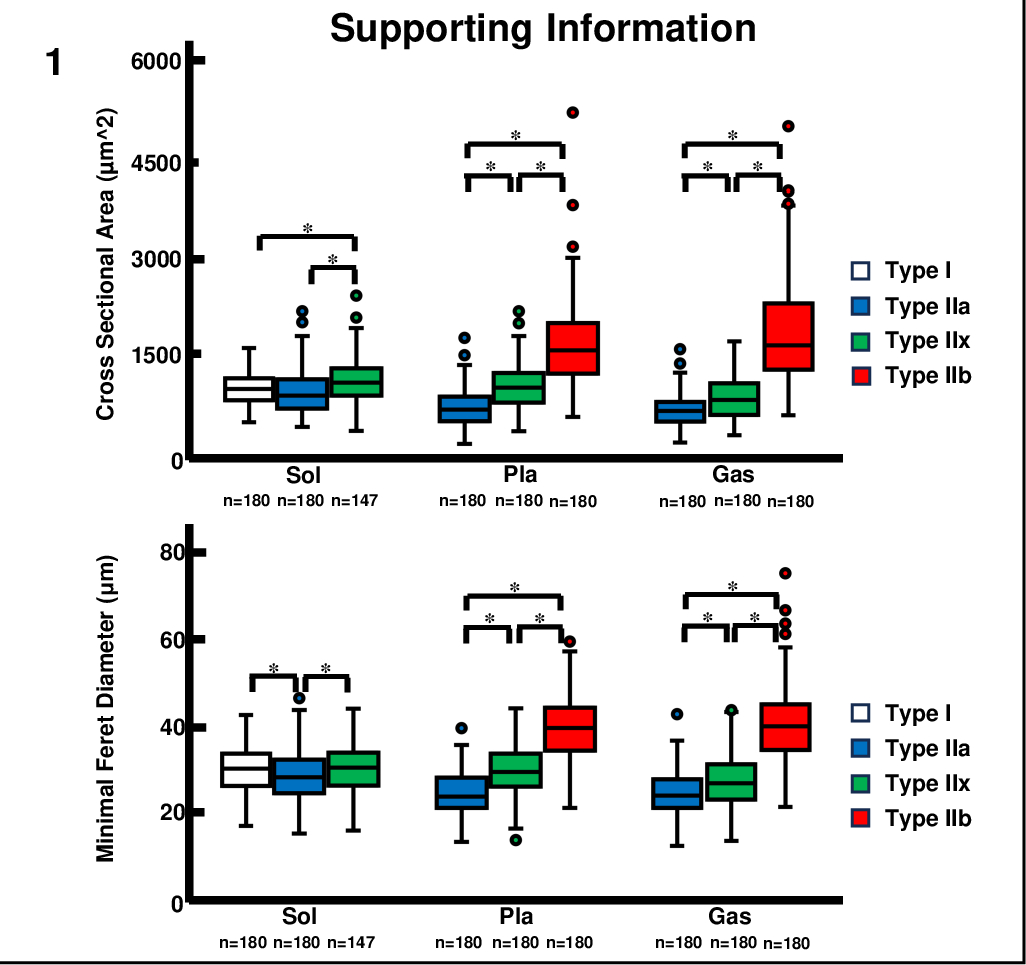

Supplement: S1 Fig — Regarding CSA, type IIx muscle fibers (1144.9 ± 27.7 µm2) were larger than type I (1036.6 ± 18.3 µm2; p = 0.001) and IIa (966.8 ± 24.0 µm2; p < 0.001) in Sol muscle. In Pla and Gas muscles, type IIb muscle fibers (1695.4 ± 46.6 µm2 and 1883.7 ± 57.3 µm2) were larger than type IIa (749.7 ± 21.9 µm2 and 702.4 ± 17.7 µm2; p < 0.001) and IIx (1064.2 ± 25.1 µm2 and 888.0 ± 25.0 µm2; p < 0.001). Regarding the Minimal Feret diameter, type IIa muscle fibers (28.3 ± 0.4 µm) are smaller than type I (29.8 ± 0.4 µm; p = 0.010) and IIx (30.0 ± 0.4 µm; p = 0.007), in Sol muscle. In Pla and Gas muscles, type IIb muscle fibers (39.1 ± 0.5 µm and 40.1 ± 0.7 µm) are larger than type IIa (23.9 ± 0.4 µm and 24.0 ± 0.4 µm; p < 0.001) and IIx (29.4 ± 0.5 µm and 27.0 ± 0.4 µm; p < 0.001). MyHC, myosin heavy chain; Sol: Soleus muscle; Pla: Plantaris muscle; Gas: Gastrocnemius muscle; HGF: Hepatocyte growth factor. Statistically significant differences between the two groups at p < 0.0167, as indicated by (*). (TIF) [file pone.0321839.s001.tif]

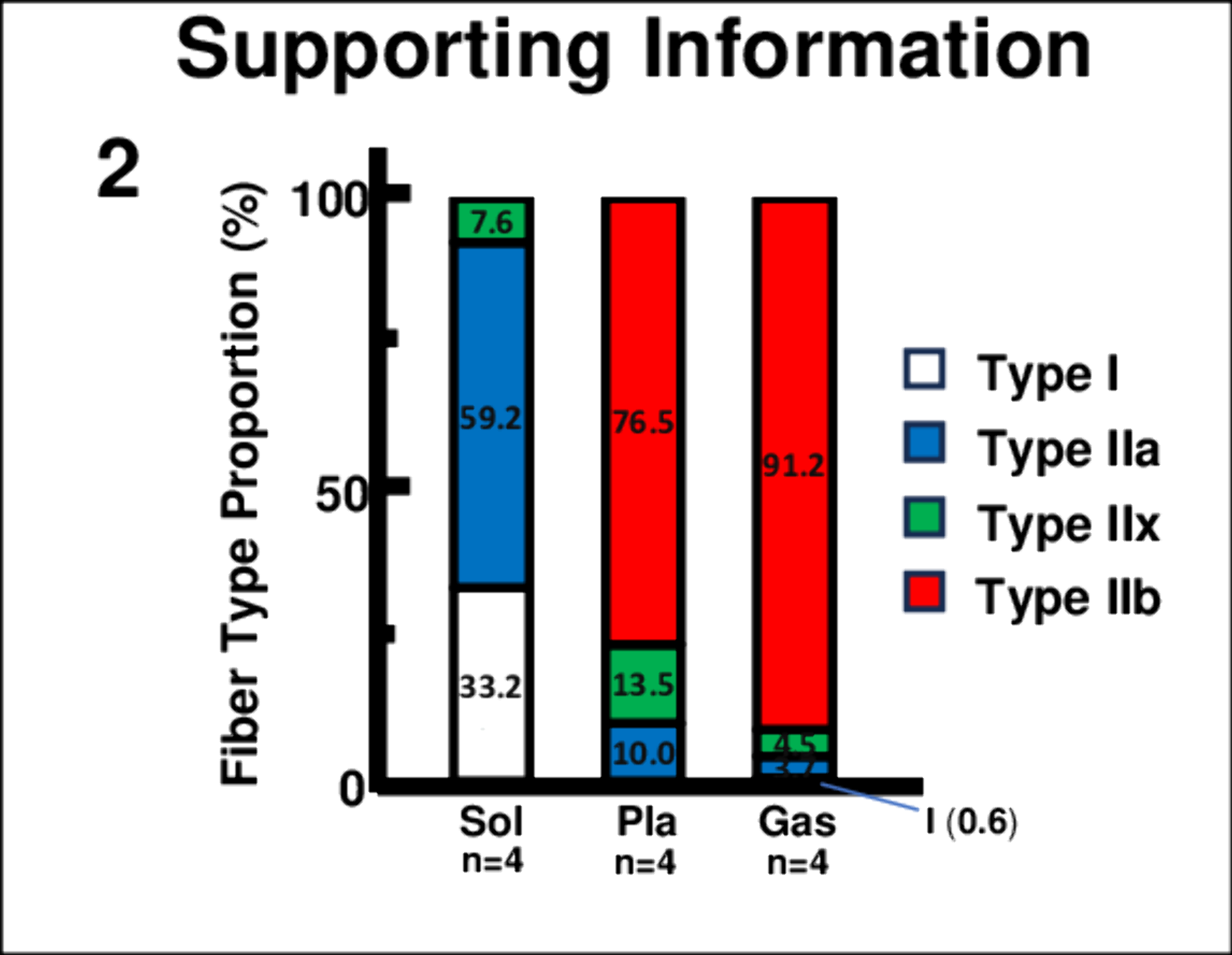

Supplement: S2 Fig — In Sol muscle, type I muscle fibers accounted for 33.2%, type IIa muscle fibers were 59.2%, and type IIx muscle fibers were 7.6%. In Pla muscle, type IIa muscle fibers accounted for 10.0%, type IIx muscle fibers for 13.5%, and type IIb muscle fibers for 76.5%. In Gas muscle, type I muscle fibers accounted for 0.6%, type IIa muscle fibers for 3.7%, type IIx muscle fibers for 4.5%, and type IIb muscle fibers for 91.2%. (TIF) [file pone.0321839.s002.tif]

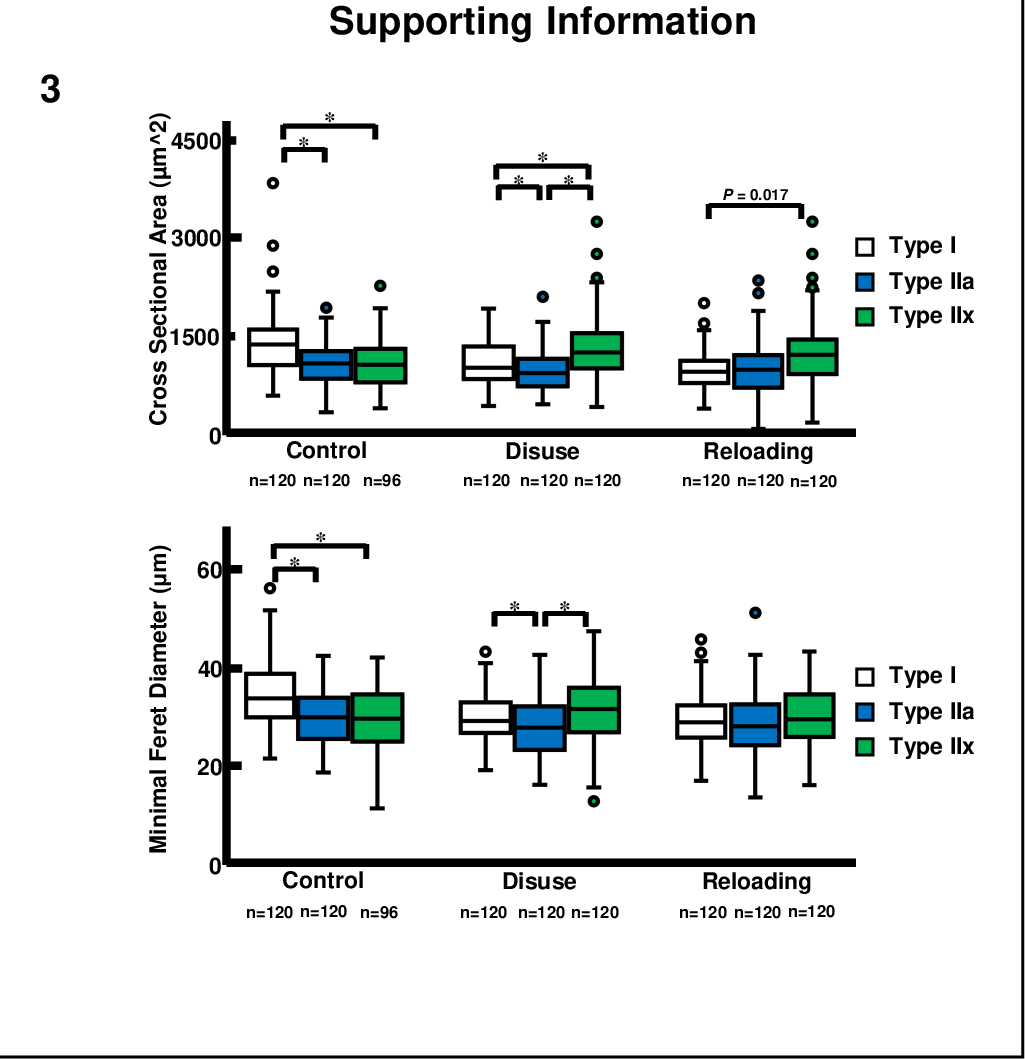

Supplement: S3 Fig — Regarding CSA, type I muscle fiber (1360.5 ± 43.4 µm2) were lager than IIa (1058.3 ± 28.1 µm2; p < 0.001) and IIx muscle fibers (1060.1 ± 38.1 µm2; p < 0.001) in the control group. In the disuse group, type IIx muscle fibers (1283.0 ± 47.8 µm2) were larger than type I (1071.7 ± 29.5 µm2; p < 0.001) and IIa (949.0 ± 27.8 µm2; p < 0.001). In the reloading group, type I (958.4 ± 25.0 µm2), IIa (984.4 ± 35.7 µm2), and IIx muscle fibers (1051.2 ± 29.4 µm2) showed no differences in size. Regarding the minimal Feret diameter, type I muscle fiber (34.2 ± 0.6 µm) were lager than IIa (29.8 ± 0.5 µm; p < 0.001) and IIx muscle fibers (29.2 ± 0.7 µm; p < 0.001) in the control group. In the disuse group, type IIa muscle fibers (28.1 ± 0.5 µm) were smaller than type I (29.9 ± 0.5 µm; p = 0.011) and IIx (31.5 ± 0.6 µm; p < 0.001). In the reloading group, type I (29.2 ± 0.5 µm), IIa (28.7 ± 0.6 µm), and IIx muscle fibers (29.9 ± 0.5 µm) showed no differences in size. (TIF) [file pone.0321839.s003.tif]

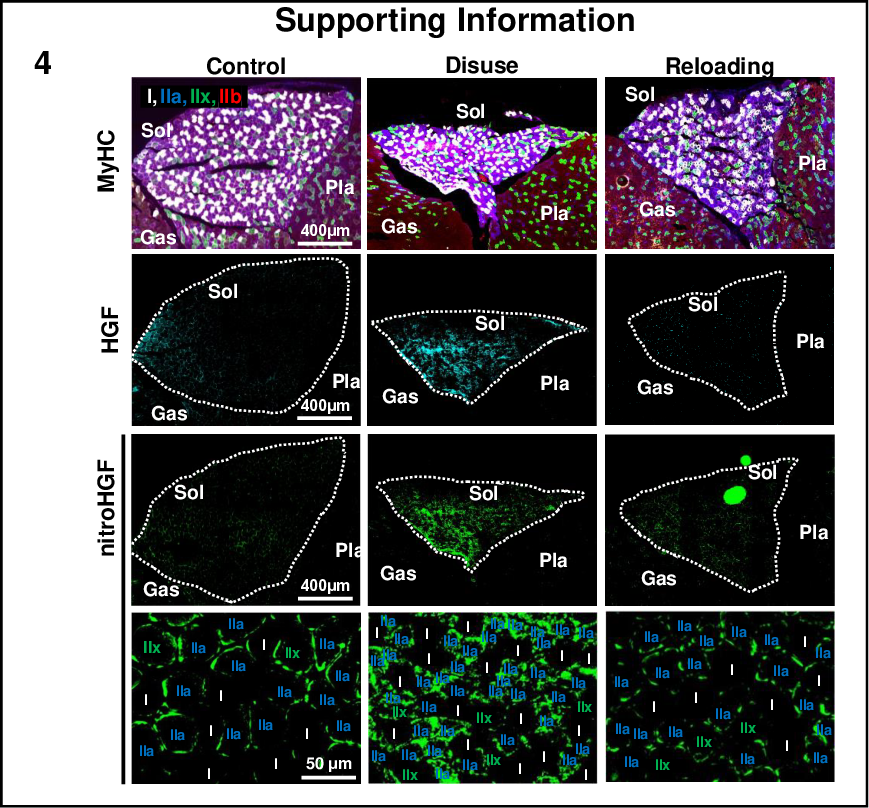

Supplement: S4 Fig — NitroHGF in the ECM was more in the disuse group than that in the control group and less in the reloading group than in the disuse group. In addition, HGF in the ECM was more in the disuse group than in the control group and less in the reloading group than in the disuse group. (TIF) [file pone.0321839.s004.tif]

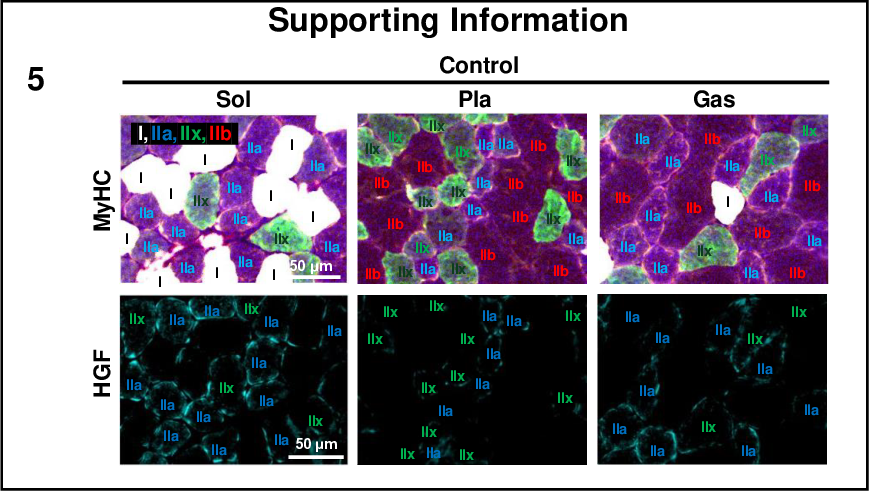

Supplement: S5 Fig — The amount of HGF around type IIa and IIx muscle fibers in the Sol was more than that in the Pla and Gas muscles. (TIF) [file pone.0321839.s005.tif]
