# Supplementary material for: Distribution of myogenic stem cell activator, hepatocyte growth factor, in skeletal muscle extracellular matrix and effect of short-term disuse and reloading
Source: PLoS One. 2025 Sep 3;20(9):e0321839. doi: 10.1371/journal.pone.0321839 (PMC12407438; doi:10.1371/journal.pone.0321839)
Supplement: S2 Table — This is the table of data in Fig 1b. The types of muscle fibers were identified using co-stained images of anti-MyHC and anti-Laminin antibodies, and were compared with co-stained images of anti-HGF and anti-Laminin antibodies in serial sections to randomly select 30 fibers for each muscle and muscle fiber type for each individual. Laminin (µm): The length of the ECM surrounding each muscle fiber was determined using an anti-laminin antibody. HGF (µm): Total HGF length in the ECM was determined using an anti-HGF antibody. Rate (%): Distribution rate of HGF in the ECM was calculated with dividing the total length of HGF by the length of the ECM. HGF: Hepatocyte growth factor, ECM: Extracellular matrix. (DOCX) [file pone.0321839.s007.docx]

**S2 Table. HGF distribution in the ECM of each muscle fiber type in the Sol, Pla, and Gas muscles (n=147–180).**

|  | Sol (%) |  | Pla (%) | Gas (%) |
| --- | --- | --- | --- | --- |
| Type | ave±s.e. | Type | ave±s.e. | ave±s.e. |
| I | 32.33±1.04 | IIa | 55.77±0.87 | 58.78±1.49 |
| IIa | 61.51±0.96 | IIx | 49.56±0.94 | 48.86±1.07 |
| IIx | 56.68±1.07 | IIb | 18.21±0.86 | 12.96±0.96 |
| t-test / Bonferroni correction: α = 0.0133 (0.05/3) | | | | |
|  | Sol |  | Pla | Gas |
| Type | t-test | Type | t-test | t-test |
| I:IIa | 0.0000 | IIa:IIx | 0.0000 | 0.0000 |
| I:IIx | 0.0000 | IIa:IIb | 0.0000 | 0.0000 |
| IIa:IIx | 0.0009 | IIx:IIb | 0.0000 | 0.0000 |

Each data

| Sample1 | Type | Laminin (µm) | HGF (µm) | Rate (%) |
| --- | --- | --- | --- | --- |
| Sol | I | 101.56 | 9.09 | 8.95 |
|  |  | 109.30 | 18.34 | 16.78 |
|  |  | 112.43 | 14.99 | 13.33 |
|  |  | 109.35 | 16.49 | 15.08 |
|  |  | 101.10 | 22.05 | 21.81 |
|  |  | 101.68 | 22.99 | 22.61 |
|  |  | 93.76 | 19.69 | 20.99 |
|  |  | 117.53 | 24.10 | 20.51 |
|  |  | 109.59 | 9.43 | 8.60 |
|  |  | 116.26 | 14.61 | 12.56 |
|  |  | 126.47 | 11.24 | 8.89 |
|  |  | 110.28 | 8.09 | 7.33 |
|  |  | 121.49 | 19.98 | 16.45 |
|  |  | 121.63 | 14.45 | 11.88 |
|  |  | 115.56 | 7.39 | 6.39 |
|  |  | 112.95 | 31.28 | 27.70 |
|  |  | 107.89 | 16.56 | 15.35 |
|  |  | 120.42 | 26.20 | 21.75 |
|  |  | 85.11 | 2.91 | 3.42 |
|  |  | 107.82 | 18.80 | 17.44 |
|  |  | 142.68 | 15.22 | 10.67 |
|  |  | 146.82 | 42.12 | 28.69 |
|  |  | 117.75 | 42.76 | 36.32 |
|  |  | 97.86 | 29.33 | 29.97 |
|  |  | 122.93 | 43.21 | 35.15 |
|  |  | 126.72 | 13.74 | 10.85 |
|  |  | 118.84 | 12.62 | 10.62 |
|  |  | 140.76 | 37.81 | 26.86 |
|  |  | 137.71 | 18.38 | 13.34 |
|  |  | 119.42 | 15.76 | 13.19 |
|  | IIa | 47.21 | 30.21 | 63.99 |
|  |  | 77.96 | 31.05 | 39.83 |
|  |  | 97.31 | 45.99 | 47.26 |
|  |  | 61.78 | 48.82 | 79.02 |
|  |  | 114.98 | 82.86 | 72.07 |
|  |  | 106.77 | 61.29 | 57.41 |
|  |  | 85.75 | 54.76 | 63.86 |
|  |  | 88.75 | 50.23 | 56.60 |
|  |  | 98.45 | 48.50 | 49.26 |
|  |  | 119.17 | 58.01 | 48.67 |
|  |  | 86.58 | 50.24 | 58.03 |
|  |  | 114.86 | 80.50 | 70.08 |
|  |  | 116.62 | 67.70 | 58.05 |
|  |  | 97.14 | 53.63 | 55.21 |
|  |  | 108.61 | 59.78 | 55.04 |
|  |  | 113.21 | 44.67 | 39.45 |
|  |  | 110.30 | 59.19 | 53.66 |
|  |  | 128.33 | 62.01 | 48.32 |
|  |  | 75.17 | 59.58 | 79.25 |
|  |  | 94.38 | 35.30 | 37.40 |
|  |  | 83.42 | 57.94 | 69.46 |
|  |  | 117.34 | 64.60 | 55.05 |
|  |  | 118.07 | 50.20 | 42.51 |
|  |  | 131.51 | 69.42 | 52.79 |
|  |  | 114.68 | 68.05 | 59.34 |
|  |  | 161.61 | 64.65 | 40.01 |
|  |  | 133.59 | 112.93 | 84.54 |
|  |  | 119.46 | 62.50 | 52.32 |
|  |  | 124.17 | 107.47 | 86.55 |
|  |  | 125.71 | 87.82 | 69.86 |
|  | IIx | 90.37 | 43.81 | 48.48 |
|  |  | 106.33 | 60.68 | 57.06 |
|  |  | 96.48 | 50.48 | 52.32 |
|  |  | 134.13 | 76.61 | 57.11 |
|  |  | 122.43 | 51.55 | 42.10 |
|  |  | 111.93 | 58.73 | 52.47 |
|  |  | 147.88 | 62.37 | 42.18 |
|  |  | 113.78 | 47.24 | 41.52 |
|  |  | 131.60 | 82.41 | 62.62 |
|  |  | 88.68 | 37.52 | 42.31 |
|  |  | 75.85 | 33.64 | 44.35 |
|  |  | 128.46 | 85.71 | 66.72 |
|  |  | 115.83 | 71.23 | 61.49 |
|  |  | 93.74 | 56.42 | 60.19 |
|  |  |  |  |  |
|  |  |  |  |  |
|  |  |  |  |  |
|  |  |  |  |  |
|  |  |  |  |  |
|  |  |  |  |  |
|  |  |  |  |  |
|  |  |  |  |  |
|  |  |  |  |  |
|  |  |  |  |  |
|  |  |  |  |  |
|  |  |  |  |  |
|  |  |  |  |  |
|  |  |  |  |  |
|  |  |  |  |  |
|  |  |  |  |  |
| Pla | IIa | 107.13 | 66.32 | 61.91 |
|  |  | 107.62 | 34.80 | 32.33 |
|  |  | 86.35 | 40.12 | 46.46 |
|  |  | 115.82 | 70.00 | 60.44 |
|  |  | 93.54 | 48.49 | 51.84 |
|  |  | 90.29 | 40.51 | 44.87 |
|  |  | 99.56 | 38.36 | 38.53 |
|  |  | 118.71 | 66.43 | 55.96 |
|  |  | 76.88 | 46.80 | 60.87 |
|  |  | 97.51 | 63.60 | 65.22 |
|  |  | 100.47 | 75.53 | 75.18 |
|  |  | 97.26 | 52.44 | 53.92 |
|  |  | 124.24 | 70.59 | 56.81 |
|  |  | 125.69 | 94.58 | 75.25 |
|  |  | 118.47 | 81.54 | 68.83 |
|  |  | 124.77 | 61.98 | 49.68 |
|  |  | 133.94 | 77.07 | 57.54 |
|  |  | 98.64 | 53.78 | 54.52 |
|  |  | 111.67 | 60.35 | 54.04 |
|  |  | 82.19 | 54.12 | 65.85 |
|  |  | 113.26 | 65.36 | 57.71 |
|  |  | 92.82 | 58.51 | 63.03 |
|  |  | 105.56 | 40.46 | 38.33 |
|  |  | 97.26 | 58.25 | 59.89 |
|  |  | 133.48 | 79.31 | 59.42 |
|  |  | 97.65 | 69.70 | 71.37 |
|  |  | 91.50 | 44.60 | 48.74 |
|  |  | 122.41 | 71.23 | 58.19 |
|  |  | 87.96 | 44.96 | 51.11 |
|  |  | 91.58 | 31.36 | 34.24 |
|  | IIx | 129.32 | 45.76 | 35.39 |
|  |  | 121.99 | 46.61 | 38.21 |
|  |  | 148.51 | 96.38 | 64.89 |
|  |  | 126.05 | 56.31 | 44.67 |
|  |  | 137.82 | 77.34 | 56.11 |
|  |  | 156.12 | 71.60 | 45.86 |
|  |  | 155.73 | 67.57 | 43.39 |
|  |  | 129.66 | 76.91 | 59.32 |
|  |  | 151.04 | 81.27 | 53.81 |
|  |  | 126.76 | 65.34 | 51.54 |
|  |  | 157.98 | 92.13 | 58.31 |
|  |  | 122.90 | 44.39 | 36.12 |
|  |  | 141.23 | 86.89 | 61.52 |
|  |  | 123.42 | 30.43 | 24.65 |
|  |  | 147.39 | 76.80 | 52.10 |
|  |  | 115.89 | 54.31 | 46.87 |
|  |  | 127.46 | 48.02 | 37.67 |
|  |  | 145.10 | 75.57 | 52.08 |
|  |  | 133.92 | 80.74 | 60.29 |
|  |  | 140.21 | 64.81 | 46.22 |
|  |  | 154.52 | 65.54 | 42.41 |
|  |  | 137.79 | 75.75 | 54.97 |
|  |  | 147.21 | 93.07 | 63.22 |
|  |  | 132.25 | 46.06 | 34.82 |
|  |  | 120.08 | 69.06 | 57.51 |
|  |  | 146.77 | 63.96 | 43.58 |
|  |  | 124.71 | 64.13 | 51.42 |
|  |  | 129.59 | 42.82 | 33.04 |
|  |  | 143.42 | 87.01 | 60.67 |
|  |  | 137.18 | 73.02 | 53.23 |
|  | IIb | 172.78 | 19.58 | 11.33 |
|  |  | 166.72 | 27.05 | 16.22 |
|  |  | 176.14 | 31.46 | 17.86 |
|  |  | 171.88 | 15.47 | 9.00 |
|  |  | 195.61 | 7.56 | 3.86 |
|  |  | 202.18 | 19.04 | 9.42 |
|  |  | 215.93 | 45.63 | 21.13 |
|  |  | 191.86 | 53.31 | 27.79 |
|  |  | 207.45 | 34.47 | 16.62 |
|  |  | 215.49 | 37.60 | 17.45 |
|  |  | 183.72 | 64.25 | 34.97 |
|  |  | 195.52 | 86.74 | 44.36 |
|  |  | 150.59 | 22.33 | 14.83 |
|  |  | 172.81 | 37.82 | 21.89 |
|  |  | 179.95 | 54.80 | 30.45 |
|  |  | 167.32 | 31.96 | 19.10 |
|  |  | 175.19 | 35.63 | 20.34 |
|  |  | 151.72 | 38.96 | 25.68 |
|  |  | 192.53 | 34.51 | 17.92 |
|  |  | 227.56 | 18.65 | 8.20 |
|  |  | 200.55 | 20.77 | 10.35 |
|  |  | 214.68 | 41.90 | 19.52 |
|  |  | 203.89 | 26.18 | 12.84 |
|  |  | 177.81 | 47.56 | 26.75 |
|  |  | 164.16 | 46.32 | 28.21 |
|  |  | 166.07 | 34.59 | 20.83 |
|  |  | 179.64 | 44.87 | 24.98 |
|  |  | 195.47 | 30.19 | 15.45 |
|  |  | 204.26 | 13.13 | 6.43 |
|  |  | 217.24 | 42.94 | 19.77 |
| Gas | IIa | 113.90 | 81.04 | 71.15 |
|  |  | 126.61 | 99.13 | 78.30 |
|  |  | 128.83 | 107.48 | 83.43 |
|  |  | 123.22 | 69.98 | 56.79 |
|  |  | 114.92 | 81.65 | 71.05 |
|  |  | 121.23 | 58.33 | 48.12 |
|  |  | 135.59 | 53.04 | 39.12 |
|  |  | 122.79 | 86.42 | 70.38 |
|  |  | 107.28 | 62.59 | 58.34 |
|  |  | 145.98 | 85.50 | 58.57 |
|  |  | 124.95 | 59.01 | 47.23 |
|  |  | 85.92 | 44.22 | 51.47 |
|  |  | 125.11 | 76.56 | 61.19 |
|  |  | 114.65 | 62.90 | 54.87 |
|  |  | 96.14 | 55.10 | 57.31 |
|  |  | 99.19 | 54.34 | 54.78 |
|  |  | 98.29 | 68.94 | 70.14 |
|  |  | 113.43 | 34.37 | 30.30 |
|  |  | 123.29 | 85.75 | 69.55 |
|  |  | 120.85 | 74.88 | 61.96 |
|  |  | 91.75 | 45.55 | 49.65 |
|  |  | 111.36 | 84.77 | 76.12 |
|  |  | 103.44 | 68.49 | 66.22 |
|  |  | 114.81 | 73.01 | 63.59 |
|  |  | 132.57 | 104.48 | 78.82 |
|  |  | 88.60 | 52.68 | 59.46 |
|  |  | 113.20 | 47.38 | 41.86 |
|  |  | 104.27 | 67.38 | 64.62 |
|  |  | 99.94 | 70.73 | 70.77 |
|  |  | 124.41 | 61.71 | 49.60 |
|  | IIx | 109.31 | 56.23 | 51.44 |
|  |  | 125.24 | 64.00 | 51.10 |
|  |  | 133.63 | 108.57 | 81.24 |
|  |  | 131.08 | 73.73 | 56.25 |
|  |  | 121.92 | 91.48 | 75.03 |
|  |  | 136.47 | 107.92 | 79.08 |
|  |  | 129.36 | 59.97 | 46.36 |
|  |  | 125.54 | 69.15 | 55.08 |
|  |  | 136.61 | 62.78 | 45.95 |
|  |  | 108.52 | 80.72 | 74.38 |
|  |  | 119.95 | 70.60 | 58.86 |
|  |  | 145.57 | 59.20 | 40.67 |
|  |  | 124.50 | 58.52 | 47.00 |
|  |  | 125.00 | 63.38 | 50.71 |
|  |  | 115.22 | 72.08 | 62.56 |
|  |  | 142.91 | 83.48 | 58.42 |
|  |  | 124.03 | 75.58 | 60.93 |
|  |  | 120.29 | 77.29 | 64.25 |
|  |  | 127.81 | 73.89 | 57.82 |
|  |  | 137.21 | 59.82 | 43.60 |
|  |  | 161.85 | 78.03 | 48.21 |
|  |  | 130.75 | 82.96 | 63.45 |
|  |  | 134.89 | 61.11 | 45.30 |
|  |  | 133.32 | 79.59 | 59.70 |
|  |  | 161.10 | 97.86 | 60.75 |
|  |  | 129.90 | 84.20 | 64.82 |
|  |  | 137.10 | 87.76 | 64.01 |
|  |  | 138.48 | 82.43 | 59.52 |
|  |  | 111.99 | 89.62 | 80.02 |
|  |  | 141.02 | 96.31 | 68.30 |
|  | IIb | 162.36 | 0.00 | 0.00 |
|  |  | 178.38 | 39.93 | 22.39 |
|  |  | 184.71 | 73.14 | 39.60 |
|  |  | 204.82 | 59.49 | 29.05 |
|  |  | 228.52 | 47.93 | 20.97 |
|  |  | 174.23 | 5.94 | 3.41 |
|  |  | 209.13 | 100.98 | 48.29 |
|  |  | 163.08 | 35.12 | 21.53 |
|  |  | 140.19 | 20.41 | 14.56 |
|  |  | 228.93 | 78.28 | 34.19 |
|  |  | 223.49 | 18.61 | 8.33 |
|  |  | 202.21 | 19.16 | 9.47 |
|  |  | 246.40 | 92.97 | 37.73 |
|  |  | 243.70 | 27.30 | 11.20 |
|  |  | 204.32 | 38.99 | 19.08 |
|  |  | 252.39 | 9.60 | 3.80 |
|  |  | 226.47 | 0.00 | 0.00 |
|  |  | 188.06 | 0.00 | 0.00 |
|  |  | 210.43 | 81.14 | 38.56 |
|  |  | 161.25 | 18.24 | 11.31 |
|  |  | 145.70 | 3.21 | 2.20 |
|  |  | 195.85 | 16.75 | 8.55 |
|  |  | 201.32 | 23.35 | 11.60 |
|  |  | 298.58 | 24.18 | 8.10 |
|  |  | 264.60 | 21.29 | 8.05 |
|  |  | 265.77 | 22.56 | 8.49 |
|  |  | 205.22 | 41.22 | 20.08 |
|  |  | 236.59 | 31.78 | 13.43 |
|  |  | 228.45 | 57.48 | 25.16 |
|  |  | 234.71 | 0.00 | 0.00 |
| Sample2 | Type | Laminin (µm) | HGF (µm) | rate (%) |
| Sol | I | 129.46 | 35.62 | 27.52 |
|  |  | 128.45 | 62.55 | 48.70 |
|  |  | 115.20 | 20.35 | 17.66 |
|  |  | 132.61 | 24.34 | 18.36 |
|  |  | 138.90 | 33.98 | 24.46 |
|  |  | 135.27 | 26.52 | 19.60 |
|  |  | 142.06 | 32.15 | 22.63 |
|  |  | 112.44 | 34.26 | 30.47 |
|  |  | 117.94 | 23.67 | 20.07 |
|  |  | 114.36 | 47.92 | 41.90 |
|  |  | 136.73 | 68.00 | 49.73 |
|  |  | 107.02 | 22.14 | 20.69 |
|  |  | 153.18 | 53.49 | 34.92 |
|  |  | 142.22 | 32.96 | 23.18 |
|  |  | 158.71 | 66.74 | 42.05 |
|  |  | 143.44 | 30.91 | 21.55 |
|  |  | 131.38 | 53.43 | 40.67 |
|  |  | 127.40 | 54.82 | 43.03 |
|  |  | 126.16 | 62.41 | 49.47 |
|  |  | 140.40 | 63.52 | 45.24 |
|  |  | 150.48 | 70.51 | 46.86 |
|  |  | 130.86 | 38.32 | 29.28 |
|  |  | 137.76 | 37.10 | 26.93 |
|  |  | 124.04 | 49.69 | 40.06 |
|  |  | 148.34 | 25.63 | 17.28 |
|  |  | 154.79 | 25.70 | 16.60 |
|  |  | 139.47 | 32.09 | 23.01 |
|  |  | 163.21 | 69.08 | 42.32 |
|  |  | 145.00 | 68.84 | 47.47 |
|  |  | 127.67 | 43.61 | 34.16 |
|  | IIa | 141.65 | 72.05 | 50.86 |
|  |  | 122.97 | 111.74 | 90.86 |
|  |  | 90.16 | 43.78 | 48.56 |
|  |  | 132.85 | 88.69 | 66.76 |
|  |  | 131.27 | 83.91 | 63.92 |
|  |  | 135.92 | 83.83 | 61.68 |
|  |  | 143.51 | 110.17 | 76.76 |
|  |  | 135.28 | 91.27 | 67.47 |
|  |  | 120.85 | 94.41 | 78.12 |
|  |  | 122.88 | 101.56 | 82.65 |
|  |  | 108.27 | 58.87 | 54.37 |
|  |  | 126.33 | 85.93 | 68.02 |
|  |  | 117.02 | 77.61 | 66.32 |
|  |  | 130.62 | 107.25 | 82.11 |
|  |  | 133.46 | 106.32 | 79.67 |
|  |  | 160.75 | 127.77 | 79.48 |
|  |  | 123.77 | 88.94 | 71.86 |
|  |  | 123.15 | 95.67 | 77.68 |
|  |  | 115.10 | 70.53 | 61.28 |
|  |  | 118.23 | 66.29 | 56.07 |
|  |  | 165.64 | 110.58 | 66.76 |
|  |  | 117.23 | 100.98 | 86.14 |
|  |  | 124.85 | 100.14 | 80.21 |
|  |  | 119.92 | 98.33 | 81.99 |
|  |  | 142.11 | 91.77 | 64.58 |
|  |  | 139.68 | 101.13 | 72.40 |
|  |  | 168.92 | 121.53 | 71.95 |
|  |  | 171.94 | 143.31 | 83.35 |
|  |  | 147.78 | 106.17 | 71.84 |
|  |  | 185.03 | 102.32 | 55.30 |
|  | IIx | 118.65 | 36.32 | 30.61 |
|  |  | 137.27 | 51.61 | 37.60 |
|  |  | 168.05 | 68.36 | 40.68 |
|  |  | 118.61 | 53.77 | 45.33 |
|  |  | 81.00 | 58.51 | 72.24 |
|  |  | 95.00 | 60.23 | 63.40 |
|  |  | 101.14 | 53.99 | 53.38 |
|  |  | 133.17 | 76.76 | 57.64 |
|  |  | 142.03 | 93.98 | 66.17 |
|  |  | 114.26 | 72.33 | 63.30 |
|  |  | 95.30 | 73.61 | 77.24 |
|  |  | 127.37 | 78.23 | 61.41 |
|  |  | 169.62 | 128.34 | 75.66 |
|  |  | 106.74 | 80.88 | 75.77 |
|  |  | 115.27 | 99.13 | 86.00 |
|  |  | 182.88 | 160.11 | 87.55 |
|  |  | 142.68 | 106.33 | 74.52 |
|  |  | 123.99 | 87.19 | 70.32 |
|  |  | 162.59 | 102.53 | 63.06 |
|  |  | 160.73 | 83.15 | 51.73 |
|  |  | 143.99 | 109.93 | 76.35 |
|  |  | 150.64 | 73.18 | 48.58 |
|  |  | 145.88 | 52.24 | 35.81 |
|  |  | 130.52 | 83.49 | 63.96 |
|  |  | 191.49 | 128.49 | 67.10 |
|  |  | 179.08 | 81.24 | 45.37 |
|  |  | 172.77 | 93.29 | 54.00 |
|  |  | 112.64 | 79.18 | 70.30 |
|  |  | 119.86 | 83.14 | 69.36 |
|  |  | 75.88 | 48.75 | 64.25 |
| Pla | IIa | 98.47 | 51.57 | 52.37 |
|  |  | 100.63 | 29.13 | 28.94 |
|  |  | 120.97 | 115.00 | 95.06 |
|  |  | 123.44 | 76.11 | 61.66 |
|  |  | 116.19 | 86.94 | 74.83 |
|  |  | 112.00 | 67.65 | 60.40 |
|  |  | 97.00 | 55.18 | 56.89 |
|  |  | 100.10 | 35.56 | 35.52 |
|  |  | 116.54 | 62.13 | 53.31 |
|  |  | 109.22 | 70.05 | 64.14 |
|  |  | 78.20 | 57.43 | 73.44 |
|  |  | 136.17 | 78.65 | 57.76 |
|  |  | 124.66 | 76.57 | 61.43 |
|  |  | 95.99 | 60.86 | 63.40 |
|  |  | 137.79 | 85.31 | 61.91 |
|  |  | 114.48 | 80.14 | 70.01 |
|  |  | 109.75 | 63.33 | 57.70 |
|  |  | 118.43 | 80.12 | 67.65 |
|  |  | 116.48 | 49.69 | 42.66 |
|  |  | 63.07 | 28.45 | 45.11 |
|  |  | 56.99 | 40.48 | 71.02 |
|  |  | 71.20 | 42.64 | 59.88 |
|  |  | 118.24 | 67.61 | 57.18 |
|  |  | 83.67 | 48.17 | 57.58 |
|  |  | 82.02 | 48.50 | 59.14 |
|  |  | 101.54 | 56.68 | 55.83 |
|  |  | 137.33 | 114.08 | 83.07 |
|  |  | 112.86 | 76.60 | 67.87 |
|  |  | 108.66 | 56.76 | 52.24 |
|  |  | 128.17 | 70.44 | 54.96 |
|  | IIx | 112.25 | 51.76 | 46.11 |
|  |  | 156.66 | 89.04 | 56.83 |
|  |  | 109.38 | 81.98 | 74.95 |
|  |  | 165.35 | 74.67 | 45.16 |
|  |  | 140.83 | 71.43 | 50.72 |
|  |  | 134.43 | 82.55 | 61.40 |
|  |  | 165.56 | 87.91 | 53.10 |
|  |  | 110.33 | 66.12 | 59.93 |
|  |  | 159.27 | 96.79 | 60.77 |
|  |  | 127.09 | 111.75 | 87.93 |
|  |  | 127.95 | 94.20 | 73.62 |
|  |  | 114.17 | 54.34 | 47.59 |
|  |  | 97.29 | 89.02 | 91.50 |
|  |  | 130.98 | 78.46 | 59.90 |
|  |  | 134.74 | 86.85 | 64.46 |
|  |  | 126.77 | 74.87 | 59.06 |
|  |  | 146.45 | 84.35 | 57.59 |
|  |  | 133.44 | 91.55 | 68.61 |
|  |  | 149.30 | 84.13 | 56.35 |
|  |  | 121.28 | 83.72 | 69.03 |
|  |  | 104.84 | 84.69 | 80.78 |
|  |  | 112.14 | 47.18 | 42.08 |
|  |  | 102.46 | 56.77 | 55.41 |
|  |  | 80.33 | 33.73 | 41.99 |
|  |  | 126.40 | 75.17 | 59.47 |
|  |  | 130.74 | 47.57 | 36.39 |
|  |  | 86.03 | 51.56 | 59.93 |
|  |  | 109.94 | 41.63 | 37.86 |
|  |  | 98.96 | 47.50 | 48.00 |
|  |  | 128.24 | 64.04 | 49.94 |
|  | IIb | 203.00 | 20.47 | 10.09 |
|  |  | 211.79 | 44.56 | 21.04 |
|  |  | 184.19 | 26.52 | 14.40 |
|  |  | 158.47 | 39.84 | 25.14 |
|  |  | 187.24 | 33.41 | 17.85 |
|  |  | 174.85 | 18.30 | 10.47 |
|  |  | 188.35 | 7.48 | 3.97 |
|  |  | 199.95 | 14.83 | 7.42 |
|  |  | 245.46 | 17.17 | 6.99 |
|  |  | 212.58 | 33.66 | 15.83 |
|  |  | 179.49 | 25.60 | 14.26 |
|  |  | 154.63 | 45.08 | 29.16 |
|  |  | 154.94 | 39.67 | 25.60 |
|  |  | 176.10 | 57.17 | 32.47 |
|  |  | 196.52 | 25.78 | 13.12 |
|  |  | 201.52 | 0.00 | 0.00 |
|  |  | 170.86 | 10.57 | 6.18 |
|  |  | 151.87 | 20.42 | 13.45 |
|  |  | 151.33 | 50.52 | 33.38 |
|  |  | 218.14 | 88.62 | 40.63 |
|  |  | 179.87 | 64.36 | 35.78 |
|  |  | 212.82 | 90.46 | 42.51 |
|  |  | 181.34 | 28.61 | 15.78 |
|  |  | 156.00 | 12.91 | 8.27 |
|  |  | 216.14 | 27.77 | 12.85 |
|  |  | 195.99 | 53.40 | 27.25 |
|  |  | 194.36 | 0.00 | 0.00 |
|  |  | 213.33 | 34.89 | 16.35 |
|  |  | 167.65 | 20.71 | 12.35 |
|  |  | 163.81 | 18.43 | 11.25 |
| Gas | IIa | 108.23 | 67.95 | 62.78 |
|  |  | 133.42 | 90.74 | 68.01 |
|  |  | 104.25 | 84.96 | 81.50 |
|  |  | 124.30 | 70.10 | 56.40 |
|  |  | 106.91 | 54.08 | 50.58 |
|  |  | 113.28 | 59.24 | 52.30 |
|  |  | 84.01 | 41.48 | 49.37 |
|  |  | 113.95 | 66.63 | 58.47 |
|  |  | 103.36 | 63.38 | 61.32 |
|  |  | 111.15 | 60.10 | 54.07 |
|  |  | 80.90 | 53.91 | 66.64 |
|  |  | 113.19 | 65.40 | 57.78 |
|  |  | 116.26 | 60.74 | 52.25 |
|  |  | 104.16 | 65.68 | 63.06 |
|  |  | 99.46 | 54.98 | 55.28 |
|  |  | 85.18 | 51.77 | 60.78 |
|  |  | 135.45 | 104.76 | 77.34 |
|  |  | 95.81 | 78.54 | 81.98 |
|  |  | 113.91 | 62.39 | 54.77 |
|  |  | 172.36 | 118.69 | 68.87 |
|  |  | 118.75 | 104.32 | 87.85 |
|  |  | 108.90 | 84.50 | 77.60 |
|  |  | 108.33 | 64.27 | 59.33 |
|  |  | 114.13 | 70.09 | 61.41 |
|  |  | 126.16 | 62.10 | 49.22 |
|  |  | 122.32 | 76.37 | 62.43 |
|  |  | 105.63 | 49.73 | 47.08 |
|  |  | 95.89 | 56.03 | 58.43 |
|  |  | 119.02 | 84.39 | 70.90 |
|  |  | 115.79 | 83.67 | 72.26 |
|  | IIx | 180.77 | 136.99 | 75.78 |
|  |  | 136.07 | 54.99 | 40.41 |
|  |  | 163.00 | 90.32 | 55.41 |
|  |  | 113.31 | 61.32 | 54.12 |
|  |  | 123.15 | 78.64 | 63.85 |
|  |  | 148.42 | 105.37 | 70.99 |
|  |  | 142.01 | 94.24 | 66.37 |
|  |  | 126.75 | 75.11 | 59.26 |
|  |  | 180.03 | 118.55 | 65.85 |
|  |  | 136.29 | 78.40 | 57.52 |
|  |  | 178.08 | 96.22 | 54.04 |
|  |  | 144.95 | 68.04 | 46.94 |
|  |  | 139.08 | 119.48 | 85.91 |
|  |  | 113.60 | 61.59 | 54.22 |
|  |  | 146.96 | 76.10 | 51.78 |
|  |  | 123.13 | 71.66 | 58.20 |
|  |  | 153.54 | 86.08 | 56.06 |
|  |  | 170.43 | 68.67 | 40.30 |
|  |  | 152.27 | 99.62 | 65.42 |
|  |  | 163.72 | 50.40 | 30.78 |
|  |  | 141.04 | 44.41 | 31.49 |
|  |  | 119.06 | 69.99 | 58.79 |
|  |  | 134.14 | 80.75 | 60.20 |
|  |  | 139.48 | 70.33 | 50.42 |
|  |  | 136.48 | 67.97 | 49.80 |
|  |  | 114.02 | 39.30 | 34.47 |
|  |  | 151.01 | 76.60 | 50.72 |
|  |  | 143.10 | 67.40 | 47.10 |
|  |  | 108.29 | 30.46 | 28.13 |
|  |  | 132.87 | 72.62 | 54.66 |
|  | IIb | 243.57 | 84.03 | 34.50 |
|  |  | 217.47 | 20.32 | 9.34 |
|  |  | 212.27 | 26.89 | 12.67 |
|  |  | 180.58 | 7.23 | 4.01 |
|  |  | 183.47 | 4.96 | 2.70 |
|  |  | 202.92 | 0.00 | 0.00 |
|  |  | 228.99 | 14.65 | 6.40 |
|  |  | 179.23 | 50.71 | 28.29 |
|  |  | 234.52 | 52.24 | 22.28 |
|  |  | 269.41 | 27.23 | 10.11 |
|  |  | 253.42 | 65.93 | 26.01 |
|  |  | 176.98 | 29.00 | 16.38 |
|  |  | 193.67 | 61.61 | 31.81 |
|  |  | 202.58 | 41.59 | 20.53 |
|  |  | 211.91 | 79.23 | 37.39 |
|  |  | 219.55 | 0.00 | 0.00 |
|  |  | 173.58 | 33.61 | 19.36 |
|  |  | 199.30 | 95.52 | 47.93 |
|  |  | 218.52 | 54.85 | 25.10 |
|  |  | 226.67 | 22.79 | 10.05 |
|  |  | 365.31 | 78.00 | 21.35 |
|  |  | 271.37 | 33.54 | 12.36 |
|  |  | 239.26 | 45.43 | 18.99 |
|  |  | 221.72 | 14.11 | 6.36 |
|  |  | 202.95 | 0.00 | 0.00 |
|  |  | 177.31 | 7.26 | 4.09 |
|  |  | 192.94 | 21.48 | 11.13 |
|  |  | 132.16 | 15.93 | 12.05 |
|  |  | 178.70 | 0.00 | 0.00 |
|  |  | 202.65 | 62.10 | 30.65 |
| Sample3 | Type | Laminin (µm) | HGF (µm) | rate (%) |
| Sol | I | 149.98 | 46.66 | 31.11 |
|  |  | 134.40 | 24.97 | 18.58 |
|  |  | 130.94 | 54.96 | 41.98 |
|  |  | 122.02 | 73.80 | 60.48 |
|  |  | 130.04 | 53.79 | 41.36 |
|  |  | 138.55 | 56.61 | 40.86 |
|  |  | 130.86 | 32.92 | 25.16 |
|  |  | 129.87 | 77.65 | 59.79 |
|  |  | 127.08 | 44.87 | 35.30 |
|  |  | 131.61 | 49.92 | 37.93 |
|  |  | 133.85 | 49.00 | 36.61 |
|  |  | 118.43 | 35.05 | 29.59 |
|  |  | 140.99 | 38.61 | 27.38 |
|  |  | 111.29 | 29.93 | 26.89 |
|  |  | 121.73 | 58.32 | 47.91 |
|  |  | 113.61 | 37.50 | 33.01 |
|  |  | 125.59 | 38.82 | 30.91 |
|  |  | 151.12 | 72.86 | 48.21 |
|  |  | 141.24 | 50.74 | 35.93 |
|  |  | 162.01 | 71.68 | 44.24 |
|  |  | 134.75 | 63.13 | 46.85 |
|  |  | 112.31 | 30.40 | 27.07 |
|  |  | 135.88 | 85.15 | 62.67 |
|  |  | 138.43 | 73.81 | 53.32 |
|  |  | 138.41 | 66.03 | 47.70 |
|  |  | 108.49 | 21.90 | 20.19 |
|  |  | 122.94 | 36.51 | 29.70 |
|  |  | 128.14 | 21.19 | 16.54 |
|  |  | 154.07 | 91.48 | 59.38 |
|  |  | 124.41 | 37.26 | 29.95 |
|  | IIa | 130.00 | 88.94 | 68.41 |
|  |  | 154.35 | 52.42 | 33.96 |
|  |  | 113.45 | 102.23 | 90.11 |
|  |  | 132.00 | 67.61 | 51.22 |
|  |  | 139.13 | 93.89 | 67.49 |
|  |  | 114.56 | 74.46 | 65.00 |
|  |  | 137.54 | 119.01 | 86.53 |
|  |  | 147.65 | 64.28 | 43.54 |
|  |  | 151.83 | 96.18 | 63.35 |
|  |  | 126.47 | 94.60 | 74.80 |
|  |  | 109.53 | 94.52 | 86.29 |
|  |  | 131.60 | 98.07 | 74.52 |
|  |  | 144.63 | 88.44 | 61.15 |
|  |  | 125.08 | 49.31 | 39.42 |
|  |  | 141.74 | 91.04 | 64.23 |
|  |  | 146.09 | 69.24 | 47.40 |
|  |  | 141.05 | 92.86 | 65.83 |
|  |  | 108.29 | 78.52 | 72.50 |
|  |  | 126.20 | 72.24 | 57.24 |
|  |  | 126.25 | 88.26 | 69.91 |
|  |  | 122.58 | 92.17 | 75.19 |
|  |  | 123.25 | 65.91 | 53.47 |
|  |  | 130.03 | 62.61 | 48.15 |
|  |  | 127.50 | 92.56 | 72.59 |
|  |  | 144.04 | 57.23 | 39.73 |
|  |  | 116.77 | 67.58 | 57.88 |
|  |  | 105.66 | 81.00 | 76.66 |
|  |  | 145.08 | 65.82 | 45.37 |
|  |  | 119.22 | 74.05 | 62.11 |
|  |  | 130.87 | 109.51 | 83.68 |
|  | IIx | 134.12 | 68.86 | 51.34 |
|  |  | 125.94 | 56.49 | 44.85 |
|  |  | 129.00 | 109.63 | 84.99 |
|  |  | 141.91 | 95.10 | 67.01 |
|  |  | 137.12 | 85.11 | 62.07 |
|  |  | 163.13 | 88.36 | 54.17 |
|  |  | 124.38 | 74.98 | 60.28 |
|  |  | 156.05 | 88.63 | 56.80 |
|  |  | 157.58 | 89.73 | 56.94 |
|  |  | 139.12 | 88.42 | 63.56 |
|  |  | 148.13 | 77.42 | 52.26 |
|  |  | 110.81 | 81.21 | 73.29 |
|  |  | 135.12 | 82.88 | 61.34 |
|  |  | 155.06 | 118.05 | 76.13 |
|  |  | 128.86 | 100.56 | 78.04 |
|  |  | 154.66 | 65.25 | 42.19 |
|  |  | 149.50 | 134.56 | 90.01 |
|  |  | 133.61 | 62.21 | 46.56 |
|  |  | 127.82 | 62.25 | 48.70 |
|  |  | 134.56 | 95.38 | 70.88 |
|  |  | 106.85 | 50.87 | 47.60 |
|  |  | 127.93 | 42.03 | 32.85 |
|  |  | 113.58 | 61.90 | 54.50 |
|  |  |  |  |  |
|  |  |  |  |  |
|  |  |  |  |  |
|  |  |  |  |  |
|  |  |  |  |  |
|  |  |  |  |  |
|  |  |  |  |  |
| Pla | IIa | 97.60 | 49.00 | 50.21 |
|  |  | 92.85 | 29.88 | 32.18 |
|  |  | 100.90 | 41.28 | 40.92 |
|  |  | 108.24 | 33.34 | 30.80 |
|  |  | 81.85 | 35.25 | 43.06 |
|  |  | 96.45 | 51.37 | 53.26 |
|  |  | 112.80 | 51.16 | 45.35 |
|  |  | 96.86 | 68.17 | 70.38 |
|  |  | 100.24 | 99.83 | 99.60 |
|  |  | 93.96 | 53.72 | 57.17 |
|  |  | 85.18 | 52.27 | 61.37 |
|  |  | 105.22 | 74.38 | 70.70 |
|  |  | 125.70 | 81.63 | 64.94 |
|  |  | 107.83 | 71.67 | 66.47 |
|  |  | 120.24 | 73.30 | 60.96 |
|  |  | 124.09 | 80.63 | 64.98 |
|  |  | 82.86 | 49.65 | 59.92 |
|  |  | 114.46 | 77.56 | 67.75 |
|  |  | 101.77 | 76.87 | 75.53 |
|  |  | 104.59 | 57.84 | 55.30 |
|  |  | 115.42 | 65.13 | 56.43 |
|  |  | 100.08 | 59.85 | 59.81 |
|  |  | 91.63 | 44.60 | 48.67 |
|  |  | 116.86 | 49.95 | 42.74 |
|  |  | 117.77 | 72.86 | 61.87 |
|  |  | 114.54 | 78.97 | 68.95 |
|  |  | 91.36 | 53.64 | 58.72 |
|  |  | 118.84 | 52.48 | 44.16 |
|  |  | 101.02 | 29.81 | 29.51 |
|  |  | 112.25 | 51.25 | 45.66 |
|  | IIx | 116.02 | 49.65 | 42.79 |
|  |  | 141.41 | 74.68 | 52.81 |
|  |  | 155.90 | 60.77 | 38.98 |
|  |  | 140.87 | 62.68 | 44.50 |
|  |  | 144.30 | 44.68 | 30.97 |
|  |  | 125.92 | 61.15 | 48.57 |
|  |  | 127.84 | 33.14 | 25.93 |
|  |  | 102.40 | 70.80 | 69.14 |
|  |  | 122.62 | 44.69 | 36.45 |
|  |  | 132.79 | 53.12 | 40.00 |
|  |  | 148.73 | 48.19 | 32.40 |
|  |  | 103.48 | 72.99 | 70.54 |
|  |  | 129.71 | 46.97 | 36.21 |
|  |  | 125.42 | 73.50 | 58.60 |
|  |  | 172.36 | 74.22 | 43.06 |
|  |  | 163.23 | 88.60 | 54.28 |
|  |  | 137.52 | 72.27 | 52.55 |
|  |  | 170.56 | 78.07 | 45.77 |
|  |  | 143.45 | 53.80 | 37.50 |
|  |  | 175.57 | 77.34 | 44.05 |
|  |  | 154.62 | 71.82 | 46.45 |
|  |  | 147.41 | 58.19 | 39.48 |
|  |  | 136.45 | 51.61 | 37.82 |
|  |  | 153.38 | 71.30 | 46.49 |
|  |  | 126.74 | 34.33 | 27.09 |
|  |  | 140.67 | 22.95 | 16.31 |
|  |  | 139.86 | 62.36 | 44.59 |
|  |  | 181.35 | 76.09 | 41.96 |
|  |  | 165.69 | 110.57 | 66.73 |
|  |  | 185.12 | 98.45 | 53.18 |
|  | IIb | 180.80 | 52.95 | 29.28 |
|  |  | 160.05 | 46.26 | 28.90 |
|  |  | 159.29 | 19.88 | 12.48 |
|  |  | 168.36 | 29.24 | 17.37 |
|  |  | 120.85 | 28.77 | 23.81 |
|  |  | 144.07 | 17.49 | 12.14 |
|  |  | 176.56 | 21.85 | 12.37 |
|  |  | 168.22 | 16.05 | 9.54 |
|  |  | 145.50 | 43.18 | 29.68 |
|  |  | 175.05 | 55.25 | 31.56 |
|  |  | 133.90 | 36.81 | 27.49 |
|  |  | 169.15 | 54.65 | 32.31 |
|  |  | 146.46 | 36.62 | 25.00 |
|  |  | 155.13 | 24.40 | 15.73 |
|  |  | 179.02 | 69.01 | 38.55 |
|  |  | 147.16 | 35.83 | 24.34 |
|  |  | 160.38 | 56.06 | 34.95 |
|  |  | 156.62 | 42.43 | 27.09 |
|  |  | 175.79 | 71.37 | 40.60 |
|  |  | 198.48 | 53.80 | 27.11 |
|  |  | 181.37 | 59.56 | 32.84 |
|  |  | 163.59 | 43.63 | 26.67 |
|  |  | 153.33 | 42.30 | 27.59 |
|  |  | 161.02 | 37.43 | 23.25 |
|  |  | 167.38 | 55.69 | 33.27 |
|  |  | 140.22 | 24.39 | 17.39 |
|  |  | 157.82 | 35.87 | 22.73 |
|  |  | 188.87 | 50.77 | 26.88 |
|  |  | 176.35 | 41.86 | 23.74 |
|  |  | 177.94 | 36.84 | 20.70 |
| Gas | IIa | 74.72 | 69.05 | 92.40 |
|  |  | 88.09 | 47.04 | 53.40 |
|  |  | 90.39 | 62.72 | 69.39 |
|  |  | 98.53 | 93.32 | 94.71 |
|  |  | 90.39 | 84.87 | 93.89 |
|  |  | 85.28 | 76.41 | 89.60 |
|  |  | 92.68 | 71.31 | 76.94 |
|  |  | 94.85 | 55.23 | 58.23 |
|  |  | 87.90 | 62.40 | 70.99 |
|  |  | 90.85 | 44.56 | 49.05 |
|  |  | 119.30 | 64.03 | 53.67 |
|  |  | 86.14 | 34.35 | 39.88 |
|  |  | 68.39 | 27.49 | 40.19 |
|  |  | 67.05 | 29.96 | 44.68 |
|  |  | 68.21 | 17.42 | 25.54 |
|  |  | 79.39 | 57.39 | 72.29 |
|  |  | 69.58 | 38.61 | 55.49 |
|  |  | 80.56 | 38.11 | 47.31 |
|  |  | 75.10 | 43.43 | 57.83 |
|  |  | 94.38 | 46.68 | 49.45 |
|  |  | 84.54 | 37.01 | 43.78 |
|  |  | 95.76 | 30.17 | 31.50 |
|  |  | 92.72 | 61.58 | 66.42 |
|  |  | 85.48 | 26.08 | 30.51 |
|  |  | 80.28 | 53.81 | 67.02 |
|  |  | 71.62 | 25.22 | 35.22 |
|  |  | 83.30 | 49.69 | 59.65 |
|  |  | 83.43 | 31.11 | 37.29 |
|  |  | 97.20 | 31.67 | 32.59 |
|  |  | 89.67 | 36.87 | 41.12 |
|  | IIx | 141.74 | 82.41 | 58.14 |
|  |  | 99.05 | 70.74 | 71.43 |
|  |  | 121.91 | 61.19 | 50.19 |
|  |  | 106.65 | 99.63 | 93.42 |
|  |  | 93.01 | 63.00 | 67.73 |
|  |  | 90.64 | 55.32 | 61.03 |
|  |  | 108.80 | 45.48 | 41.80 |
|  |  | 113.25 | 64.34 | 56.82 |
|  |  | 162.29 | 81.31 | 50.10 |
|  |  | 124.08 | 64.41 | 51.91 |
|  |  | 127.62 | 55.12 | 43.19 |
|  |  | 150.12 | 63.98 | 42.62 |
|  |  | 120.09 | 48.74 | 40.59 |
|  |  | 140.46 | 40.86 | 29.09 |
|  |  | 132.14 | 67.76 | 51.28 |
|  |  | 131.79 | 26.82 | 20.35 |
|  |  | 88.44 | 48.27 | 54.58 |
|  |  | 112.85 | 47.98 | 42.51 |
|  |  | 73.52 | 19.23 | 26.15 |
|  |  | 88.20 | 26.34 | 29.87 |
|  |  | 111.85 | 18.57 | 16.60 |
|  |  | 121.02 | 43.99 | 36.35 |
|  |  | 103.81 | 21.99 | 21.18 |
|  |  | 119.43 | 39.56 | 33.12 |
|  |  | 120.94 | 28.98 | 23.96 |
|  |  | 139.23 | 27.21 | 19.54 |
|  |  | 132.28 | 20.68 | 15.63 |
|  |  | 154.71 | 47.59 | 30.76 |
|  |  | 152.22 | 35.18 | 23.11 |
|  |  | 145.33 | 56.82 | 39.10 |
|  | IIb | 227.22 | 0.00 | 0.00 |
|  |  | 229.24 | 0.00 | 0.00 |
|  |  | 185.16 | 13.13 | 7.09 |
|  |  | 215.99 | 38.89 | 18.00 |
|  |  | 226.50 | 14.61 | 6.45 |
|  |  | 201.33 | 24.51 | 12.17 |
|  |  | 190.38 | 25.69 | 13.49 |
|  |  | 181.11 | 0.00 | 0.00 |
|  |  | 150.30 | 0.00 | 0.00 |
|  |  | 147.54 | 3.79 | 2.57 |
|  |  | 149.75 | 11.88 | 7.93 |
|  |  | 131.97 | 14.76 | 11.18 |
|  |  | 141.69 | 64.41 | 45.45 |
|  |  | 139.70 | 45.95 | 32.89 |
|  |  | 183.94 | 33.42 | 18.17 |
|  |  | 163.03 | 11.61 | 7.12 |
|  |  | 166.93 | 0.00 | 0.00 |
|  |  | 178.66 | 0.00 | 0.00 |
|  |  | 202.65 | 0.00 | 0.00 |
|  |  | 185.38 | 17.45 | 9.41 |
|  |  | 209.38 | 36.78 | 17.57 |
|  |  | 181.78 | 14.71 | 8.09 |
|  |  | 184.92 | 36.21 | 19.58 |
|  |  | 139.86 | 0.00 | 0.00 |
|  |  | 196.30 | 0.00 | 0.00 |
|  |  | 147.46 | 5.25 | 3.56 |
|  |  | 173.12 | 26.53 | 15.32 |
|  |  | 184.30 | 2.54 | 1.38 |
|  |  | 170.88 | 0.00 | 0.00 |
|  |  | 199.42 | 0.00 | 0.00 |
| Sample4 | Type | Laminin (µm) | HGF (µm) | rate (%) |
| Sol | I | 129.42 | 40.46 | 31.27 |
|  |  | 137.92 | 79.33 | 57.52 |
|  |  | 137.80 | 54.60 | 39.62 |
|  |  | 122.92 | 63.84 | 51.94 |
|  |  | 146.52 | 47.72 | 32.57 |
|  |  | 133.67 | 45.12 | 33.76 |
|  |  | 128.06 | 76.32 | 59.59 |
|  |  | 136.02 | 41.58 | 30.57 |
|  |  | 142.72 | 31.30 | 21.93 |
|  |  | 138.53 | 52.80 | 38.11 |
|  |  | 143.81 | 59.62 | 41.46 |
|  |  | 193.31 | 77.95 | 40.33 |
|  |  | 136.47 | 55.81 | 40.90 |
|  |  | 115.62 | 43.29 | 37.44 |
|  |  | 159.16 | 62.55 | 39.30 |
|  |  | 151.08 | 88.44 | 58.54 |
|  |  | 128.70 | 54.98 | 42.72 |
|  |  | 140.56 | 41.11 | 29.25 |
|  |  | 134.22 | 69.10 | 51.48 |
|  |  | 149.62 | 82.85 | 55.37 |
|  |  | 152.60 | 81.95 | 53.70 |
|  |  | 144.21 | 53.59 | 37.16 |
|  |  | 139.35 | 45.70 | 32.79 |
|  |  | 130.00 | 68.87 | 52.98 |
|  |  | 134.09 | 26.97 | 20.11 |
|  |  | 143.61 | 44.28 | 30.83 |
|  |  | 123.61 | 47.62 | 38.52 |
|  |  | 162.98 | 59.68 | 36.62 |
|  |  | 133.72 | 59.09 | 44.19 |
|  |  | 133.35 | 47.63 | 35.72 |
|  | IIa | 119.45 | 73.42 | 61.47 |
|  |  | 129.97 | 79.12 | 60.88 |
|  |  | 115.96 | 58.55 | 50.49 |
|  |  | 124.12 | 89.88 | 72.41 |
|  |  | 124.75 | 76.06 | 60.96 |
|  |  | 108.78 | 65.57 | 60.27 |
|  |  | 107.24 | 75.86 | 70.74 |
|  |  | 147.65 | 103.82 | 70.31 |
|  |  | 125.44 | 82.06 | 65.42 |
|  |  | 158.01 | 94.01 | 59.50 |
|  |  | 127.55 | 85.44 | 66.98 |
|  |  | 173.36 | 114.73 | 66.18 |
|  |  | 127.75 | 98.76 | 77.31 |
|  |  | 159.18 | 101.00 | 63.45 |
|  |  | 172.69 | 113.93 | 65.97 |
|  |  | 153.83 | 118.11 | 76.78 |
|  |  | 169.16 | 111.93 | 66.17 |
|  |  | 145.96 | 93.75 | 64.23 |
|  |  | 146.44 | 93.10 | 63.57 |
|  |  | 152.92 | 92.10 | 60.23 |
|  |  | 129.73 | 91.17 | 70.28 |
|  |  | 119.15 | 90.79 | 76.19 |
|  |  | 143.11 | 93.74 | 65.50 |
|  |  | 161.42 | 103.49 | 64.11 |
|  |  | 108.84 | 58.80 | 54.03 |
|  |  | 104.45 | 43.29 | 41.45 |
|  |  | 103.39 | 43.39 | 41.97 |
|  |  | 136.80 | 59.98 | 43.84 |
|  |  | 123.44 | 75.67 | 61.30 |
|  |  | 162.72 | 122.42 | 75.23 |
|  | IIx | 128.16 | 77.57 | 60.52 |
|  |  | 115.51 | 81.78 | 70.79 |
|  |  | 127.81 | 54.09 | 42.32 |
|  |  | 133.08 | 74.76 | 56.18 |
|  |  | 94.19 | 52.39 | 55.62 |
|  |  | 132.03 | 55.04 | 41.69 |
|  |  | 113.40 | 49.49 | 43.64 |
|  |  | 119.75 | 48.50 | 40.50 |
|  |  | 137.00 | 85.40 | 62.33 |
|  |  | 141.42 | 68.50 | 48.44 |
|  |  | 140.52 | 84.31 | 60.00 |
|  |  | 135.28 | 68.70 | 50.78 |
|  |  | 134.10 | 77.54 | 57.82 |
|  |  | 166.94 | 135.48 | 81.16 |
|  |  | 128.86 | 104.75 | 81.29 |
|  |  | 133.07 | 80.62 | 60.58 |
|  |  | 121.54 | 60.83 | 50.05 |
|  |  | 126.61 | 85.55 | 67.57 |
|  |  | 110.15 | 53.49 | 48.57 |
|  |  | 170.04 | 102.35 | 60.19 |
|  |  | 124.98 | 36.06 | 28.85 |
|  |  | 146.97 | 97.83 | 66.57 |
|  |  | 157.20 | 97.87 | 62.26 |
|  |  | 156.30 | 79.53 | 50.88 |
|  |  | 162.18 | 81.67 | 50.36 |
|  |  | 166.43 | 114.55 | 68.83 |
|  |  | 151.71 | 104.75 | 69.05 |
|  |  | 169.50 | 85.44 | 50.41 |
|  |  | 118.36 | 66.04 | 55.80 |
|  |  | 164.19 | 98.05 | 59.71 |
| Pla | IIa | 88.08 | 37.33 | 42.38 |
|  |  | 94.65 | 50.21 | 53.05 |
|  |  | 133.35 | 84.30 | 63.22 |
|  |  | 135.02 | 82.71 | 61.26 |
|  |  | 111.11 | 46.28 | 41.65 |
|  |  | 109.81 | 43.76 | 39.85 |
|  |  | 106.86 | 58.52 | 54.76 |
|  |  | 97.78 | 60.54 | 61.91 |
|  |  | 119.41 | 84.10 | 70.43 |
|  |  | 134.09 | 54.46 | 40.62 |
|  |  | 117.64 | 58.48 | 49.71 |
|  |  | 90.19 | 55.08 | 61.07 |
|  |  | 96.07 | 62.20 | 64.74 |
|  |  | 117.16 | 75.16 | 64.15 |
|  |  | 104.29 | 59.62 | 57.16 |
|  |  | 113.09 | 76.68 | 67.80 |
|  |  | 124.75 | 69.65 | 55.84 |
|  |  | 139.87 | 81.26 | 58.10 |
|  |  | 102.40 | 62.19 | 60.73 |
|  |  | 114.97 | 62.39 | 54.27 |
|  |  | 99.71 | 52.04 | 52.19 |
|  |  | 121.82 | 55.81 | 45.81 |
|  |  | 79.59 | 56.95 | 71.56 |
|  |  | 98.86 | 53.68 | 54.30 |
|  |  | 89.35 | 50.65 | 56.69 |
|  |  | 77.51 | 41.70 | 53.80 |
|  |  | 98.54 | 55.91 | 56.74 |
|  |  | 120.84 | 55.63 | 46.04 |
|  |  | 100.06 | 45.84 | 45.81 |
|  |  | 91.26 | 41.16 | 45.10 |
|  | IIx | 144.27 | 78.05 | 54.10 |
|  |  | 193.61 | 96.16 | 49.67 |
|  |  | 156.28 | 64.84 | 41.49 |
|  |  | 158.83 | 78.21 | 49.24 |
|  |  | 131.53 | 79.88 | 60.73 |
|  |  | 164.70 | 83.17 | 50.50 |
|  |  | 160.33 | 83.24 | 51.92 |
|  |  | 148.94 | 81.79 | 54.91 |
|  |  | 133.24 | 73.43 | 55.11 |
|  |  | 140.41 | 50.63 | 36.06 |
|  |  | 126.40 | 53.09 | 42.00 |
|  |  | 127.59 | 75.47 | 59.15 |
|  |  | 128.46 | 32.96 | 25.66 |
|  |  | 122.79 | 47.27 | 38.50 |
|  |  | 117.89 | 67.73 | 57.45 |
|  |  | 125.77 | 71.39 | 56.77 |
|  |  | 136.61 | 65.97 | 48.29 |
|  |  | 143.59 | 70.72 | 49.25 |
|  |  | 168.77 | 87.70 | 51.96 |
|  |  | 146.91 | 84.34 | 57.41 |
|  |  | 153.64 | 68.81 | 44.79 |
|  |  | 132.68 | 65.66 | 49.49 |
|  |  | 126.84 | 49.28 | 38.85 |
|  |  | 179.15 | 80.28 | 44.81 |
|  |  | 146.65 | 71.15 | 48.51 |
|  |  | 127.00 | 44.67 | 35.17 |
|  |  | 112.72 | 59.60 | 52.87 |
|  |  | 144.92 | 37.83 | 26.10 |
|  |  | 117.62 | 67.06 | 57.01 |
|  |  | 119.82 | 58.37 | 48.71 |
|  | IIb | 153.11 | 0.00 | 0.00 |
|  |  | 174.96 | 18.65 | 10.66 |
|  |  | 176.42 | 19.15 | 10.85 |
|  |  | 204.49 | 55.22 | 27.00 |
|  |  | 201.45 | 28.23 | 14.01 |
|  |  | 197.61 | 12.17 | 6.16 |
|  |  | 171.08 | 29.52 | 17.25 |
|  |  | 174.85 | 0.00 | 0.00 |
|  |  | 163.86 | 19.89 | 12.14 |
|  |  | 122.47 | 23.19 | 18.93 |
|  |  | 195.29 | 58.77 | 30.09 |
|  |  | 174.30 | 73.40 | 42.11 |
|  |  | 123.60 | 10.80 | 8.74 |
|  |  | 154.46 | 48.55 | 31.43 |
|  |  | 139.25 | 45.95 | 32.99 |
|  |  | 186.52 | 76.33 | 40.93 |
|  |  | 187.91 | 13.25 | 7.05 |
|  |  | 225.61 | 47.76 | 21.17 |
|  |  | 206.04 | 40.79 | 19.80 |
|  |  | 116.65 | 9.10 | 7.80 |
|  |  | 150.28 | 24.97 | 16.61 |
|  |  | 129.18 | 0.00 | 0.00 |
|  |  | 134.93 | 6.51 | 4.83 |
|  |  | 182.71 | 14.58 | 7.98 |
|  |  | 203.71 | 29.84 | 14.65 |
|  |  | 190.32 | 37.04 | 19.46 |
|  |  | 208.39 | 15.38 | 7.38 |
|  |  | 175.60 | 53.44 | 30.43 |
|  |  | 143.76 | 44.06 | 30.65 |
|  |  | 184.43 | 0.00 | 0.00 |
| Gas | IIa | 139.77 | 134.11 | 95.94 |
|  |  | 114.33 | 59.00 | 51.61 |
|  |  | 169.00 | 104.86 | 62.05 |
|  |  | 118.71 | 60.74 | 51.16 |
|  |  | 101.77 | 79.45 | 78.08 |
|  |  | 114.25 | 78.98 | 69.13 |
|  |  | 119.75 | 61.53 | 51.38 |
|  |  | 95.60 | 49.17 | 51.43 |
|  |  | 99.63 | 56.90 | 57.11 |
|  |  | 128.07 | 56.00 | 43.72 |
|  |  | 126.69 | 73.53 | 58.04 |
|  |  | 118.77 | 67.32 | 56.68 |
|  |  | 127.98 | 43.04 | 33.63 |
|  |  | 101.27 | 30.73 | 30.34 |
|  |  | 120.11 | 59.59 | 49.61 |
|  |  | 93.73 | 48.36 | 51.60 |
|  |  | 116.32 | 52.71 | 45.31 |
|  |  | 113.25 | 76.37 | 67.44 |
|  |  | 148.09 | 97.36 | 65.75 |
|  |  | 112.91 | 65.60 | 58.09 |
|  |  | 122.36 | 68.56 | 56.03 |
|  |  | 108.11 | 83.37 | 77.12 |
|  |  | 126.80 | 65.41 | 51.59 |
|  |  | 114.75 | 61.84 | 53.89 |
|  |  | 108.56 | 80.56 | 74.21 |
|  |  | 110.61 | 65.34 | 59.08 |
|  |  | 113.91 | 46.06 | 40.43 |
|  |  | 106.42 | 68.06 | 63.95 |
|  |  | 127.54 | 73.81 | 57.87 |
|  |  | 107.05 | 71.71 | 66.99 |
|  | IIx | 121.81 | 63.70 | 52.29 |
|  |  | 120.07 | 52.64 | 43.85 |
|  |  | 103.09 | 38.26 | 37.12 |
|  |  | 115.38 | 50.83 | 44.05 |
|  |  | 116.62 | 49.51 | 42.45 |
|  |  | 147.96 | 35.99 | 24.32 |
|  |  | 108.03 | 43.59 | 40.35 |
|  |  | 133.87 | 64.00 | 47.81 |
|  |  | 133.25 | 53.01 | 39.78 |
|  |  | 106.74 | 57.54 | 53.90 |
|  |  | 144.92 | 73.24 | 50.54 |
|  |  | 175.18 | 103.33 | 58.99 |
|  |  | 159.44 | 87.56 | 54.92 |
|  |  | 181.50 | 60.31 | 33.23 |
|  |  | 152.23 | 60.75 | 39.90 |
|  |  | 81.68 | 25.25 | 30.91 |
|  |  | 69.88 | 35.74 | 51.15 |
|  |  | 105.58 | 39.94 | 37.82 |
|  |  | 118.34 | 38.80 | 32.78 |
|  |  | 154.23 | 62.70 | 40.65 |
|  |  | 137.76 | 69.48 | 50.44 |
|  |  | 130.21 | 52.87 | 40.61 |
|  |  | 122.12 | 46.99 | 38.48 |
|  |  | 158.42 | 51.83 | 32.72 |
|  |  | 160.75 | 44.11 | 27.44 |
|  |  | 147.91 | 36.04 | 24.37 |
|  |  | 147.33 | 75.12 | 50.99 |
|  |  | 143.63 | 62.22 | 43.32 |
|  |  | 145.01 | 73.20 | 50.48 |
|  |  | 168.93 | 77.88 | 46.10 |
|  | IIb | 188.66 | 41.22 | 21.85 |
|  |  | 175.91 | 39.71 | 22.57 |
|  |  | 207.31 | 14.97 | 7.22 |
|  |  | 194.37 | 0.00 | 0.00 |
|  |  | 250.80 | 45.20 | 18.02 |
|  |  | 243.83 | 0.00 | 0.00 |
|  |  | 190.15 | 0.00 | 0.00 |
|  |  | 264.63 | 5.24 | 1.98 |
|  |  | 222.54 | 14.93 | 6.71 |
|  |  | 190.45 | 43.18 | 22.68 |
|  |  | 230.00 | 51.41 | 22.35 |
|  |  | 265.71 | 0.00 | 0.00 |
|  |  | 179.05 | 13.94 | 7.79 |
|  |  | 137.01 | 72.89 | 53.20 |
|  |  | 164.66 | 16.35 | 9.93 |
|  |  | 219.09 | 0.00 | 0.00 |
|  |  | 177.16 | 0.00 | 0.00 |
|  |  | 166.28 | 34.70 | 20.87 |
|  |  | 189.50 | 28.64 | 15.11 |
|  |  | 177.92 | 0.00 | 0.00 |
|  |  | 187.65 | 0.00 | 0.00 |
|  |  | 185.03 | 53.84 | 29.10 |
|  |  | 153.09 | 55.40 | 36.18 |
|  |  | 188.65 | 40.72 | 21.58 |
|  |  | 236.70 | 35.63 | 15.05 |
|  |  | 188.00 | 0.00 | 0.00 |
|  |  | 226.95 | 0.00 | 0.00 |
|  |  | 231.82 | 60.83 | 26.24 |
|  |  | 208.41 | 19.58 | 9.40 |
|  |  | 183.30 | 6.90 | 3.77 |
| Sample5 | Type | Laminin (µm) | HGF (µm) | rate (%) |
| Sol | I | 135.49 | 66.17 | 48.84 |
|  |  | 132.42 | 57.88 | 43.71 |
|  |  | 163.28 | 21.16 | 12.96 |
|  |  | 133.99 | 72.71 | 54.27 |
|  |  | 115.65 | 35.86 | 31.01 |
|  |  | 127.64 | 37.58 | 29.44 |
|  |  | 121.53 | 56.66 | 46.62 |
|  |  | 139.13 | 63.66 | 45.76 |
|  |  | 133.66 | 42.46 | 31.76 |
|  |  | 119.94 | 29.14 | 24.30 |
|  |  | 131.81 | 94.61 | 71.78 |
|  |  | 157.82 | 43.64 | 27.65 |
|  |  | 144.79 | 58.41 | 40.34 |
|  |  | 131.58 | 66.72 | 50.71 |
|  |  | 126.69 | 75.40 | 59.51 |
|  |  | 169.28 | 62.06 | 36.66 |
|  |  | 155.04 | 65.52 | 42.26 |
|  |  | 143.57 | 82.44 | 57.42 |
|  |  | 154.26 | 36.74 | 23.82 |
|  |  | 134.44 | 35.06 | 26.08 |
|  |  | 133.17 | 52.30 | 39.27 |
|  |  | 130.25 | 63.99 | 49.13 |
|  |  | 133.95 | 71.99 | 53.75 |
|  |  | 153.13 | 20.53 | 13.41 |
|  |  | 131.44 | 29.32 | 22.30 |
|  |  | 131.55 | 52.20 | 39.68 |
|  |  | 117.74 | 37.56 | 31.90 |
|  |  | 111.52 | 55.75 | 49.99 |
|  |  | 125.44 | 26.54 | 21.16 |
|  |  | 157.07 | 53.46 | 34.04 |
|  | IIa | 113.96 | 61.49 | 53.96 |
|  |  | 104.38 | 64.61 | 61.90 |
|  |  | 133.89 | 87.14 | 65.08 |
|  |  | 133.80 | 67.92 | 50.76 |
|  |  | 121.59 | 75.71 | 62.26 |
|  |  | 118.91 | 83.88 | 70.54 |
|  |  | 126.49 | 66.84 | 52.84 |
|  |  | 139.09 | 82.84 | 59.56 |
|  |  | 151.51 | 80.62 | 53.21 |
|  |  | 126.91 | 98.60 | 77.70 |
|  |  | 130.64 | 85.72 | 65.61 |
|  |  | 111.48 | 69.78 | 62.59 |
|  |  | 155.14 | 96.06 | 61.92 |
|  |  | 156.08 | 121.08 | 77.58 |
|  |  | 159.11 | 115.36 | 72.50 |
|  |  | 126.75 | 96.78 | 76.35 |
|  |  | 109.11 | 69.51 | 63.71 |
|  |  | 145.42 | 97.35 | 66.94 |
|  |  | 128.06 | 99.57 | 77.76 |
|  |  | 126.76 | 98.31 | 77.56 |
|  |  | 104.18 | 62.50 | 59.99 |
|  |  | 129.17 | 73.56 | 56.95 |
|  |  | 149.19 | 88.56 | 59.36 |
|  |  | 112.73 | 57.28 | 50.81 |
|  |  | 121.04 | 65.21 | 53.88 |
|  |  | 86.75 | 52.35 | 60.34 |
|  |  | 103.82 | 55.79 | 53.74 |
|  |  | 107.44 | 43.05 | 40.07 |
|  |  | 140.44 | 58.39 | 41.57 |
|  |  | 128.79 | 82.52 | 64.08 |
|  | IIx | 134.78 | 82.23 | 61.01 |
|  |  | 123.40 | 92.94 | 75.32 |
|  |  | 150.35 | 78.42 | 52.15 |
|  |  | 162.03 | 78.24 | 48.29 |
|  |  | 150.33 | 56.62 | 37.66 |
|  |  | 134.14 | 95.37 | 71.10 |
|  |  | 141.11 | 89.36 | 63.33 |
|  |  | 117.38 | 85.46 | 72.80 |
|  |  | 148.07 | 82.74 | 55.88 |
|  |  | 126.01 | 66.91 | 53.10 |
|  |  | 116.74 | 66.14 | 56.66 |
|  |  | 134.72 | 44.91 | 33.34 |
|  |  | 137.21 | 62.85 | 45.80 |
|  |  | 126.41 | 48.92 | 38.70 |
|  |  | 136.42 | 63.54 | 46.58 |
|  |  | 139.48 | 70.60 | 50.62 |
|  |  | 131.63 | 71.08 | 54.00 |
|  |  | 102.15 | 49.08 | 48.05 |
|  |  | 131.25 | 50.34 | 38.36 |
|  |  | 121.45 | 80.11 | 65.96 |
|  |  | 119.86 | 52.40 | 43.72 |
|  |  | 132.52 | 77.79 | 58.70 |
|  |  | 111.05 | 61.09 | 55.02 |
|  |  | 115.54 | 69.10 | 59.80 |
|  |  | 132.74 | 114.02 | 85.89 |
|  |  | 159.02 | 104.22 | 65.54 |
|  |  | 173.91 | 109.80 | 63.14 |
|  |  | 128.19 | 89.90 | 70.13 |
|  |  | 128.74 | 76.12 | 59.13 |
|  |  | 120.87 | 76.27 | 63.11 |
| Pla | IIa | 103.74 | 57.25 | 55.19 |
|  |  | 115.22 | 61.76 | 53.61 |
|  |  | 113.95 | 61.73 | 54.17 |
|  |  | 105.18 | 56.04 | 53.28 |
|  |  | 95.46 | 45.43 | 47.59 |
|  |  | 120.99 | 40.55 | 33.52 |
|  |  | 122.07 | 55.67 | 45.61 |
|  |  | 101.80 | 51.83 | 50.91 |
|  |  | 96.87 | 63.31 | 65.36 |
|  |  | 93.37 | 47.21 | 50.57 |
|  |  | 123.70 | 42.04 | 33.99 |
|  |  | 131.77 | 52.20 | 39.61 |
|  |  | 120.95 | 51.41 | 42.50 |
|  |  | 121.19 | 50.93 | 42.02 |
|  |  | 113.55 | 64.57 | 56.87 |
|  |  | 78.99 | 47.25 | 59.82 |
|  |  | 77.83 | 48.50 | 62.32 |
|  |  | 106.78 | 56.82 | 53.22 |
|  |  | 108.82 | 43.43 | 39.92 |
|  |  | 69.74 | 36.17 | 51.87 |
|  |  | 94.23 | 49.89 | 52.95 |
|  |  | 96.96 | 56.62 | 58.39 |
|  |  | 93.48 | 59.86 | 64.03 |
|  |  | 118.80 | 80.04 | 67.37 |
|  |  | 93.37 | 47.15 | 50.50 |
|  |  | 88.54 | 58.78 | 66.39 |
|  |  | 87.49 | 52.09 | 59.54 |
|  |  | 71.42 | 34.57 | 48.41 |
|  |  | 69.77 | 34.72 | 49.76 |
|  |  | 111.23 | 64.06 | 57.59 |
|  | IIx | 131.34 | 57.48 | 43.77 |
|  |  | 130.77 | 71.18 | 54.43 |
|  |  | 169.11 | 94.60 | 55.94 |
|  |  | 142.16 | 91.27 | 64.20 |
|  |  | 159.70 | 91.45 | 57.26 |
|  |  | 144.19 | 62.80 | 43.55 |
|  |  | 122.36 | 61.61 | 50.35 |
|  |  | 135.39 | 62.95 | 46.50 |
|  |  | 124.49 | 46.49 | 37.34 |
|  |  | 115.20 | 55.65 | 48.30 |
|  |  | 133.77 | 81.12 | 60.64 |
|  |  | 122.83 | 82.56 | 67.22 |
|  |  | 132.01 | 50.35 | 38.14 |
|  |  | 147.15 | 35.75 | 24.29 |
|  |  | 128.50 | 84.23 | 65.55 |
|  |  | 134.81 | 79.25 | 58.79 |
|  |  | 104.02 | 90.89 | 87.38 |
|  |  | 91.08 | 46.10 | 50.62 |
|  |  | 103.21 | 41.04 | 39.76 |
|  |  | 104.71 | 30.60 | 29.22 |
|  |  | 122.32 | 74.16 | 60.63 |
|  |  | 115.24 | 63.83 | 55.39 |
|  |  | 153.10 | 46.41 | 30.32 |
|  |  | 112.37 | 68.56 | 61.01 |
|  |  | 134.93 | 79.13 | 58.64 |
|  |  | 129.29 | 92.60 | 71.63 |
|  |  | 125.86 | 40.50 | 32.18 |
|  |  | 100.40 | 76.19 | 75.88 |
|  |  | 130.10 | 58.51 | 44.97 |
|  |  | 107.87 | 68.19 | 63.21 |
|  | IIb | 154.16 | 35.78 | 23.21 |
|  |  | 233.44 | 59.68 | 25.57 |
|  |  | 169.68 | 60.29 | 35.53 |
|  |  | 188.99 | 10.15 | 5.37 |
|  |  | 168.58 | 40.97 | 24.30 |
|  |  | 135.13 | 0.00 | 0.00 |
|  |  | 192.21 | 20.58 | 10.71 |
|  |  | 177.61 | 14.02 | 7.89 |
|  |  | 158.82 | 36.70 | 23.11 |
|  |  | 178.77 | 0.00 | 0.00 |
|  |  | 155.82 | 18.93 | 12.15 |
|  |  | 178.48 | 21.64 | 12.12 |
|  |  | 191.39 | 9.37 | 4.90 |
|  |  | 206.01 | 0.00 | 0.00 |
|  |  | 193.37 | 38.27 | 19.79 |
|  |  | 152.18 | 29.71 | 19.53 |
|  |  | 168.92 | 0.00 | 0.00 |
|  |  | 150.37 | 13.39 | 8.90 |
|  |  | 222.15 | 40.23 | 18.11 |
|  |  | 223.32 | 10.20 | 4.57 |
|  |  | 164.83 | 22.95 | 13.92 |
|  |  | 194.37 | 128.67 | 66.20 |
|  |  | 215.28 | 10.40 | 4.83 |
|  |  | 152.55 | 67.62 | 44.33 |
|  |  | 144.83 | 53.50 | 36.94 |
|  |  | 167.96 | 14.60 | 8.69 |
|  |  | 184.96 | 25.02 | 13.53 |
|  |  | 195.91 | 0.00 | 0.00 |
|  |  | 181.57 | 49.96 | 27.51 |
|  |  | 207.69 | 15.74 | 7.58 |
| Gas | IIa | 72.08 | 28.99 | 40.21 |
|  |  | 93.83 | 67.21 | 71.63 |
|  |  | 89.55 | 54.99 | 61.40 |
|  |  | 94.61 | 47.53 | 50.23 |
|  |  | 75.60 | 51.29 | 67.85 |
|  |  | 114.47 | 59.03 | 51.57 |
|  |  | 123.32 | 63.93 | 51.84 |
|  |  | 100.05 | 49.20 | 49.17 |
|  |  | 87.63 | 52.44 | 59.85 |
|  |  | 91.46 | 46.00 | 50.29 |
|  |  | 95.55 | 63.51 | 66.46 |
|  |  | 89.74 | 51.27 | 57.12 |
|  |  | 86.40 | 61.67 | 71.38 |
|  |  | 112.04 | 75.32 | 67.22 |
|  |  | 124.86 | 84.99 | 68.07 |
|  |  | 105.21 | 77.45 | 73.61 |
|  |  | 87.93 | 62.13 | 70.66 |
|  |  | 100.47 | 74.25 | 73.91 |
|  |  | 118.15 | 65.78 | 55.68 |
|  |  | 108.10 | 54.62 | 50.53 |
|  |  | 106.46 | 55.37 | 52.01 |
|  |  | 111.15 | 57.75 | 51.95 |
|  |  | 96.52 | 60.39 | 62.56 |
|  |  | 146.81 | 95.72 | 65.20 |
|  |  | 70.21 | 42.75 | 60.90 |
|  |  | 86.15 | 32.69 | 37.95 |
|  |  | 82.93 | 14.36 | 17.31 |
|  |  | 85.31 | 39.26 | 46.02 |
|  |  | 85.98 | 47.08 | 54.76 |
|  |  | 129.63 | 57.85 | 44.63 |
|  | IIx | 104.41 | 45.66 | 43.73 |
|  |  | 136.46 | 99.02 | 72.56 |
|  |  | 120.30 | 66.46 | 55.24 |
|  |  | 102.37 | 58.53 | 57.18 |
|  |  | 115.98 | 31.24 | 26.93 |
|  |  | 108.12 | 41.90 | 38.76 |
|  |  | 109.41 | 32.09 | 29.33 |
|  |  | 109.17 | 64.22 | 58.83 |
|  |  | 129.05 | 54.70 | 42.38 |
|  |  | 139.20 | 50.61 | 36.36 |
|  |  | 134.05 | 45.23 | 33.74 |
|  |  | 105.87 | 42.31 | 39.97 |
|  |  | 99.52 | 52.68 | 52.93 |
|  |  | 121.50 | 47.98 | 39.49 |
|  |  | 98.69 | 65.51 | 66.38 |
|  |  | 125.07 | 58.81 | 47.02 |
|  |  | 118.67 | 52.56 | 44.29 |
|  |  | 130.20 | 52.29 | 40.16 |
|  |  | 93.77 | 43.54 | 46.43 |
|  |  | 108.87 | 31.14 | 28.61 |
|  |  | 135.04 | 38.79 | 28.72 |
|  |  | 117.25 | 42.31 | 36.09 |
|  |  | 117.83 | 44.69 | 37.93 |
|  |  | 107.85 | 56.07 | 51.99 |
|  |  | 101.88 | 40.08 | 39.34 |
|  |  | 118.21 | 80.06 | 67.73 |
|  |  | 126.04 | 59.90 | 47.53 |
|  |  | 114.33 | 58.12 | 50.83 |
|  |  | 131.69 | 37.54 | 28.51 |
|  |  | 139.31 | 58.99 | 42.34 |
|  | IIb | 202.07 | 63.04 | 31.20 |
|  |  | 153.87 | 30.95 | 20.11 |
|  |  | 176.53 | 18.85 | 10.68 |
|  |  | 178.42 | 0.00 | 0.00 |
|  |  | 204.41 | 0.00 | 0.00 |
|  |  | 202.20 | 18.63 | 9.21 |
|  |  | 170.74 | 46.50 | 27.23 |
|  |  | 144.94 | 11.06 | 7.63 |
|  |  | 168.02 | 55.36 | 32.95 |
|  |  | 175.27 | 31.42 | 17.93 |
|  |  | 150.69 | 0.00 | 0.00 |
|  |  | 182.96 | 63.31 | 34.60 |
|  |  | 186.54 | 3.31 | 1.77 |
|  |  | 155.36 | 76.94 | 49.52 |
|  |  | 180.68 | 23.46 | 12.98 |
|  |  | 184.41 | 0.00 | 0.00 |
|  |  | 209.05 | 0.00 | 0.00 |
|  |  | 245.56 | 18.38 | 7.48 |
|  |  | 156.02 | 74.91 | 48.01 |
|  |  | 195.24 | 0.00 | 0.00 |
|  |  | 191.55 | 19.31 | 10.08 |
|  |  | 183.31 | 0.00 | 0.00 |
|  |  | 211.63 | 0.00 | 0.00 |
|  |  | 213.52 | 29.80 | 13.96 |
|  |  | 176.43 | 15.81 | 8.96 |
|  |  | 138.12 | 0.00 | 0.00 |
|  |  | 181.12 | 21.77 | 12.02 |
|  |  | 160.63 | 0.00 | 0.00 |
|  |  | 178.81 | 0.00 | 0.00 |
|  |  | 189.79 | 15.49 | 8.16 |
| Sample6 | Type | Laminin (µm) | HGF (µm) | rate (%) |
| Sol | I | 129.19 | 30.25 | 23.41 |
|  |  | 134.27 | 24.79 | 18.46 |
|  |  | 143.05 | 51.36 | 35.90 |
|  |  | 120.30 | 32.95 | 27.39 |
|  |  | 119.55 | 6.09 | 5.10 |
|  |  | 113.40 | 28.58 | 25.20 |
|  |  | 130.64 | 35.43 | 27.12 |
|  |  | 122.76 | 24.97 | 20.34 |
|  |  | 120.23 | 20.38 | 16.95 |
|  |  | 125.02 | 24.81 | 19.84 |
|  |  | 117.24 | 18.65 | 15.91 |
|  |  | 141.73 | 44.67 | 31.52 |
|  |  | 138.85 | 51.65 | 37.20 |
|  |  | 140.74 | 41.87 | 29.75 |
|  |  | 130.59 | 57.65 | 44.15 |
|  |  | 115.58 | 24.93 | 21.57 |
|  |  | 138.01 | 46.05 | 33.36 |
|  |  | 123.44 | 27.32 | 22.14 |
|  |  | 136.94 | 60.67 | 44.30 |
|  |  | 117.82 | 31.34 | 26.60 |
|  |  | 153.68 | 30.24 | 19.68 |
|  |  | 116.90 | 19.37 | 16.57 |
|  |  | 142.40 | 34.94 | 24.54 |
|  |  | 144.60 | 64.53 | 44.62 |
|  |  | 138.89 | 70.28 | 50.60 |
|  |  | 138.89 | 27.97 | 20.14 |
|  |  | 136.26 | 30.06 | 22.06 |
|  |  | 124.05 | 54.40 | 43.85 |
|  |  | 144.66 | 47.72 | 32.99 |
|  |  | 136.71 | 21.55 | 15.76 |
|  | IIa | 98.57 | 43.99 | 44.63 |
|  |  | 101.36 | 45.13 | 44.52 |
|  |  | 125.83 | 54.56 | 43.36 |
|  |  | 119.01 | 70.08 | 58.88 |
|  |  | 135.30 | 103.81 | 76.72 |
|  |  | 108.85 | 58.01 | 53.30 |
|  |  | 102.84 | 47.01 | 45.71 |
|  |  | 118.07 | 92.81 | 78.61 |
|  |  | 128.50 | 77.23 | 60.10 |
|  |  | 134.50 | 77.27 | 57.45 |
|  |  | 154.07 | 66.11 | 42.91 |
|  |  | 126.03 | 67.22 | 53.33 |
|  |  | 134.38 | 79.51 | 59.16 |
|  |  | 150.45 | 99.76 | 66.31 |
|  |  | 102.94 | 50.64 | 49.20 |
|  |  | 105.66 | 57.69 | 54.60 |
|  |  | 114.52 | 50.38 | 43.99 |
|  |  | 89.96 | 27.38 | 30.43 |
|  |  | 102.97 | 51.39 | 49.90 |
|  |  | 80.95 | 34.43 | 42.53 |
|  |  | 102.93 | 49.82 | 48.40 |
|  |  | 110.75 | 54.54 | 49.24 |
|  |  | 112.30 | 69.98 | 62.32 |
|  |  | 110.18 | 49.33 | 44.77 |
|  |  | 122.06 | 57.92 | 47.45 |
|  |  | 99.14 | 45.66 | 46.06 |
|  |  | 125.63 | 73.30 | 58.34 |
|  |  | 88.03 | 42.20 | 47.93 |
|  |  | 137.65 | 56.67 | 41.17 |
|  |  | 120.73 | 73.71 | 61.05 |
|  | IIx | 123.12 | 89.90 | 73.02 |
|  |  | 100.80 | 53.42 | 52.99 |
|  |  | 147.24 | 91.44 | 62.10 |
|  |  | 186.68 | 82.60 | 44.25 |
|  |  | 151.88 | 84.48 | 55.63 |
|  |  | 107.18 | 39.95 | 37.28 |
|  |  | 135.63 | 58.39 | 43.05 |
|  |  | 151.06 | 81.21 | 53.76 |
|  |  | 137.03 | 69.55 | 50.75 |
|  |  | 67.44 | 41.60 | 61.67 |
|  |  | 107.11 | 61.86 | 57.76 |
|  |  | 129.16 | 55.81 | 43.21 |
|  |  | 125.65 | 75.83 | 60.35 |
|  |  | 120.51 | 44.51 | 36.93 |
|  |  | 79.22 | 40.47 | 51.09 |
|  |  | 79.66 | 31.03 | 38.96 |
|  |  | 83.93 | 30.20 | 35.98 |
|  |  | 126.15 | 51.45 | 40.79 |
|  |  | 91.47 | 40.06 | 43.80 |
|  |  | 112.25 | 41.66 | 37.11 |
|  |  |  |  |  |
|  |  |  |  |  |
|  |  |  |  |  |
|  |  |  |  |  |
|  |  |  |  |  |
|  |  |  |  |  |
|  |  |  |  |  |
|  |  |  |  |  |
|  |  |  |  |  |
|  |  |  |  |  |
| Pla | IIa | 87.77 | 31.44 | 35.82 |
|  |  | 87.45 | 74.33 | 85.00 |
|  |  | 90.91 | 53.19 | 58.50 |
|  |  | 91.70 | 41.77 | 45.55 |
|  |  | 84.50 | 47.65 | 56.39 |
|  |  | 109.79 | 54.63 | 49.75 |
|  |  | 96.60 | 52.04 | 53.87 |
|  |  | 88.33 | 58.59 | 66.33 |
|  |  | 94.82 | 36.75 | 38.76 |
|  |  | 107.51 | 67.53 | 62.81 |
|  |  | 106.23 | 52.11 | 49.05 |
|  |  | 104.19 | 53.84 | 51.68 |
|  |  | 82.08 | 56.17 | 68.43 |
|  |  | 97.65 | 51.51 | 52.75 |
|  |  | 128.56 | 54.01 | 42.01 |
|  |  | 99.74 | 56.04 | 56.19 |
|  |  | 79.93 | 43.46 | 54.37 |
|  |  | 89.52 | 49.38 | 55.16 |
|  |  | 97.96 | 61.57 | 62.85 |
|  |  | 90.42 | 70.50 | 77.98 |
|  |  | 90.59 | 60.95 | 67.28 |
|  |  | 102.27 | 60.66 | 59.31 |
|  |  | 101.16 | 58.86 | 58.19 |
|  |  | 77.20 | 49.67 | 64.34 |
|  |  | 75.48 | 46.44 | 61.53 |
|  |  | 99.30 | 34.29 | 34.54 |
|  |  | 78.98 | 30.01 | 37.99 |
|  |  | 100.90 | 33.96 | 33.66 |
|  |  | 96.88 | 59.86 | 61.79 |
|  |  | 109.14 | 61.46 | 56.31 |
|  | IIx | 144.96 | 49.37 | 34.05 |
|  |  | 116.12 | 47.88 | 41.23 |
|  |  | 137.30 | 55.17 | 40.18 |
|  |  | 128.66 | 55.94 | 43.47 |
|  |  | 96.60 | 36.96 | 38.26 |
|  |  | 132.02 | 63.64 | 48.20 |
|  |  | 123.04 | 61.85 | 50.27 |
|  |  | 119.68 | 42.22 | 35.28 |
|  |  | 125.12 | 67.75 | 54.15 |
|  |  | 107.96 | 76.05 | 70.44 |
|  |  | 121.26 | 42.46 | 35.01 |
|  |  | 119.94 | 63.50 | 52.94 |
|  |  | 117.17 | 60.24 | 51.41 |
|  |  | 112.14 | 35.65 | 31.80 |
|  |  | 98.03 | 50.17 | 51.18 |
|  |  | 133.84 | 59.00 | 44.08 |
|  |  | 113.91 | 54.93 | 48.22 |
|  |  | 88.84 | 30.87 | 34.74 |
|  |  | 124.61 | 58.55 | 46.99 |
|  |  | 135.65 | 52.83 | 38.95 |
|  |  | 134.72 | 75.96 | 56.39 |
|  |  | 126.46 | 55.26 | 43.69 |
|  |  | 123.74 | 82.24 | 66.46 |
|  |  | 110.23 | 35.09 | 31.84 |
|  |  | 113.26 | 54.64 | 48.24 |
|  |  | 129.59 | 69.54 | 53.66 |
|  |  | 124.64 | 59.66 | 47.86 |
|  |  | 130.69 | 51.35 | 39.29 |
|  |  | 119.51 | 56.48 | 47.26 |
|  |  | 126.62 | 44.94 | 35.50 |
|  | IIb | 137.49 | 7.98 | 5.80 |
|  |  | 191.36 | 10.16 | 5.31 |
|  |  | 174.47 | 17.35 | 9.94 |
|  |  | 171.05 | 6.72 | 3.93 |
|  |  | 172.44 | 31.86 | 18.48 |
|  |  | 164.88 | 40.95 | 24.84 |
|  |  | 171.81 | 23.74 | 13.82 |
|  |  | 163.47 | 4.40 | 2.69 |
|  |  | 190.29 | 43.12 | 22.66 |
|  |  | 160.28 | 16.68 | 10.41 |
|  |  | 168.28 | 0.00 | 0.00 |
|  |  | 227.87 | 44.48 | 19.52 |
|  |  | 157.12 | 49.80 | 31.70 |
|  |  | 178.73 | 48.29 | 27.02 |
|  |  | 128.82 | 17.58 | 13.65 |
|  |  | 146.61 | 42.44 | 28.95 |
|  |  | 144.82 | 15.48 | 10.69 |
|  |  | 174.64 | 50.02 | 28.64 |
|  |  | 150.72 | 17.49 | 11.60 |
|  |  | 159.44 | 21.37 | 13.40 |
|  |  | 147.49 | 7.81 | 5.30 |
|  |  | 188.64 | 8.49 | 4.50 |
|  |  | 159.49 | 8.82 | 5.53 |
|  |  | 170.08 | 57.51 | 33.82 |
|  |  | 160.72 | 23.79 | 14.80 |
|  |  | 167.66 | 53.27 | 31.77 |
|  |  | 143.48 | 21.51 | 14.99 |
|  |  | 174.84 | 17.72 | 10.13 |
|  |  | 158.63 | 12.91 | 8.14 |
|  |  | 207.86 | 46.19 | 22.22 |
| Gas | IIa | 112.52 | 58.08 | 51.62 |
|  |  | 106.39 | 68.55 | 64.43 |
|  |  | 110.33 | 58.45 | 52.98 |
|  |  | 105.57 | 55.39 | 52.47 |
|  |  | 112.33 | 49.14 | 43.74 |
|  |  | 98.60 | 46.52 | 47.18 |
|  |  | 95.81 | 63.58 | 66.35 |
|  |  | 101.75 | 55.98 | 55.02 |
|  |  | 109.40 | 70.22 | 64.18 |
|  |  | 89.16 | 39.45 | 44.24 |
|  |  | 106.45 | 66.07 | 62.07 |
|  |  | 111.56 | 63.81 | 57.19 |
|  |  | 119.03 | 60.03 | 50.43 |
|  |  | 115.38 | 57.47 | 49.81 |
|  |  | 85.71 | 32.83 | 38.31 |
|  |  | 107.80 | 61.76 | 57.29 |
|  |  | 90.83 | 29.40 | 32.37 |
|  |  | 113.07 | 55.70 | 49.26 |
|  |  | 115.94 | 60.57 | 52.25 |
|  |  | 107.54 | 24.89 | 23.14 |
|  |  | 131.24 | 74.32 | 56.63 |
|  |  | 114.96 | 50.11 | 43.59 |
|  |  | 117.91 | 58.20 | 49.36 |
|  |  | 109.04 | 62.44 | 57.27 |
|  |  | 104.41 | 61.49 | 58.89 |
|  |  | 94.00 | 45.63 | 48.54 |
|  |  | 100.14 | 67.75 | 67.66 |
|  |  | 102.54 | 66.49 | 64.85 |
|  |  | 105.61 | 49.69 | 47.05 |
|  |  | 100.97 | 63.13 | 62.53 |
|  | IIx | 128.24 | 64.35 | 50.18 |
|  |  | 129.67 | 52.74 | 40.67 |
|  |  | 136.10 | 55.91 | 41.08 |
|  |  | 129.01 | 83.28 | 64.56 |
|  |  | 121.65 | 39.88 | 32.78 |
|  |  | 119.55 | 58.63 | 49.05 |
|  |  | 108.88 | 57.80 | 53.09 |
|  |  | 117.48 | 49.45 | 42.09 |
|  |  | 120.83 | 39.28 | 32.51 |
|  |  | 99.88 | 60.15 | 60.22 |
|  |  | 141.26 | 86.39 | 61.16 |
|  |  | 144.88 | 82.55 | 56.98 |
|  |  | 138.65 | 66.36 | 47.86 |
|  |  | 148.80 | 79.45 | 53.40 |
|  |  | 143.44 | 91.96 | 64.11 |
|  |  | 115.69 | 73.70 | 63.70 |
|  |  | 160.32 | 100.08 | 62.42 |
|  |  | 127.04 | 78.38 | 61.69 |
|  |  | 119.19 | 63.14 | 52.97 |
|  |  | 131.25 | 52.07 | 39.67 |
|  |  | 116.58 | 60.32 | 51.75 |
|  |  | 116.88 | 92.48 | 79.12 |
|  |  | 145.24 | 95.21 | 65.56 |
|  |  | 129.82 | 43.27 | 33.33 |
|  |  | 136.77 | 72.02 | 52.66 |
|  |  | 125.48 | 76.89 | 61.28 |
|  |  | 127.19 | 77.32 | 60.80 |
|  |  | 145.05 | 54.08 | 37.29 |
|  |  | 168.14 | 76.98 | 45.79 |
|  |  | 119.23 | 58.08 | 48.71 |
|  | IIb | 198.46 | 32.07 | 16.16 |
|  |  | 206.52 | 57.69 | 27.93 |
|  |  | 196.60 | 67.55 | 34.36 |
|  |  | 212.08 | 0.00 | 0.00 |
|  |  | 189.50 | 0.00 | 0.00 |
|  |  | 182.17 | 51.09 | 28.04 |
|  |  | 221.93 | 14.23 | 6.41 |
|  |  | 204.38 | 64.53 | 31.57 |
|  |  | 192.04 | 45.51 | 23.70 |
|  |  | 166.54 | 44.30 | 26.60 |
|  |  | 178.15 | 0.00 | 0.00 |
|  |  | 155.41 | 0.00 | 0.00 |
|  |  | 170.32 | 0.00 | 0.00 |
|  |  | 252.99 | 18.01 | 7.12 |
|  |  | 203.37 | 28.23 | 13.88 |
|  |  | 172.25 | 0.00 | 0.00 |
|  |  | 208.98 | 10.34 | 4.95 |
|  |  | 201.55 | 7.21 | 3.57 |
|  |  | 146.31 | 15.84 | 10.82 |
|  |  | 155.70 | 36.02 | 23.13 |
|  |  | 167.32 | 17.24 | 10.30 |
|  |  | 189.22 | 58.12 | 30.71 |
|  |  | 170.92 | 35.22 | 20.61 |
|  |  | 156.63 | 48.08 | 30.70 |
|  |  | 175.82 | 8.27 | 4.71 |
|  |  | 229.01 | 0.00 | 0.00 |
|  |  | 160.99 | 0.00 | 0.00 |
|  |  | 213.20 | 0.00 | 0.00 |
|  |  | 187.37 | 12.10 | 6.46 |
|  |  | 211.24 | 34.01 | 16.10 |
