# Supplementary material for: Distribution of myogenic stem cell activator, hepatocyte growth factor, in skeletal muscle extracellular matrix and effect of short-term disuse and reloading
Source: PLoS One. 2025 Sep 3;20(9):e0321839. doi: 10.1371/journal.pone.0321839 (PMC12407438; doi:10.1371/journal.pone.0321839)
Supplement: S3 Table — This is the table of data in Fig 2b. Body weight and the weight of each skeletal muscle were measured for each individual, and the ratio was calculated. To calculate relative values, the ratio of skeletal muscle weight to body weight in the disuse and reloading groups was divided by the ratio of skeletal muscle weight to body weight in the control group. BW: Body weight. (DOCX) [file pone.0321839.s008.docx]

**S3 Table. Relative muscle weight to body weight in the control, disuse, and reloading groups (n=4).**

| ave±s.e. | Control | Disuse | Reloading |
| --- | --- | --- | --- |
| Sol/BW(mg/g) | 0.261±0.007 | 0.253±0.015 | 0.255±0.007 |
| Sol/BW  (Relative value) | 1.000±0.026 | 0.969±0.057 | 0.976±0.025 |
| t-test / Bonferroni correction: α = 0.0167 (0.05/3) | | | |
| t-test | Con:Dis | Dis:Re | Con:Re |
| P value | 0.6404 | 0.9212 | 0.5300 |

Each data

| Control | Con1 | Con2 | Con3 | Con4 |
| --- | --- | --- | --- | --- |
| Sol(mg) | 6.5 | 6.3 | 7.0 | 6.3 |
| BW(g) | 24.56 | 24.22 | 25.36 | 25.91 |
| Sol/BW(mg/g) | 0.2647 | 0.2601 | 0.2760 | 0.2431 |
| Disuse | Dis1 | Dis2 | Dis3 | Dis4 |
| Sol(mg) | 6.9 | 6.1 | 5.9 | 4.8 |
| BW(g) | 24.89 | 22.47 | 23.48 | 22.64 |
| Sol/BW(mg/g) | 0.2772 | 0.2715 | 0.2513 | 0.2120 |
| Reloading | Re1 | Re2 | Re3 | Re4 |
| Sol(mg) | 5.9 | 7.2 | 5.8 | 6.4 |
| BW(g) | 24.16 | 26.75 | 23.89 | 24.38 |
| Sol/BW(mg/g) | 0.2442 | 0.2692 | 0.2428 | 0.2625 |
