# Supplementary material for: Distribution of myogenic stem cell activator, hepatocyte growth factor, in skeletal muscle extracellular matrix and effect of short-term disuse and reloading
Source: PLoS One. 2025 Sep 3;20(9):e0321839. doi: 10.1371/journal.pone.0321839 (PMC12407438; doi:10.1371/journal.pone.0321839)
Supplement: S4 Table — This is the table of data in Fig 2c. The types of muscle fibers were identified using co-stained images of anti-MyHC and Laminin antibodies to randomly select 30 fibers for each muscle and muscle fiber type for each individual, and myofiber size was evaluated by measuring myofiber cross-sectional area (CSA) and the Minimal Feret’s diameter as short diameter, using morphometric measurements (ImageJ software). (DOCX) [file pone.0321839.s009.docx]

**S4 Table. Cross sectional area and minimal Feret diameter of each muscle fiber in the first experiment (n=96–120).**

| Cross sectional area (µm^2^) | | | |
| --- | --- | --- | --- |
|  | Control (µm^2^) | Disuse (µm^2^) | Reloading (µm^2^) |
| Type | ave±s.e | ave±s.e | ave±s.e |
| I | 1360.50±43.38 | 1071.69±29.51 | 958.43±25.02 |
| IIa | 1058.25±28.11 | 949.01±27.82 | 984.44±35.68 |
| IIx | 1060.12±38.13 | 1291.32±46.90 | 1051.21±29.44 |
| t-test / Bonferroni correction: α = 0.0133 (0.05/3) | | | |
|  | Con:Dis | Dis:Re | Con:Re |
| Type | t-test | t-test | t-test |
| I | 0.0000 | 0.0038 | 0.0000 |
| IIa | 0.0062 | 0.4344 | 0.1056 |
| IIx | 0.0003 | 0.0001 | 0.8534 |
| Minimal Feret diameter (µm) | | | |
|  | Control (µm) | Disuse (µm) | Reloading (µm) |
| Type | ave±s.e | ave±s.e | ave±s.e |
| I | 34.24±0.60 | 29.94±0.48 | 29.24±0.47 |
| IIa | 29.75±0.49 | 28.10±0.53 | 28.69±0.57 |
| IIx | 29.18±0.72 | 31.46±0.64 | 29.93±0.53 |
| t-test / Bonferroni correction: α = 0.0133 (0.05/3) | | | |
|  | Con:Dis | Dis:Re | Con:Re |
| Type | t-test | t-test | t-test |
| I | 0.0000 | 0.3031 | 0.0000 |
| IIa | 0.0231 | 0.1598 | 0.4447 |
| IIx | 0.0191 | 0.0663 | 0.3997 |

Each data

| Cross sectional area (µm^2^) | | | | |
| --- | --- | --- | --- | --- |
|  | Control |  |  |  |
| Type | Con1 | Con2 | Con3 | Con4 |
| I | 569.19 | 1586.15 | 1103.02 | 1199.39 |
|  | 760.67 | 1584.46 | 1097.97 | 908.59 |
|  | 1080.51 | 963.51 | 983.71 | 1068.51 |
|  | 913.01 | 1123.22 | 1505.98 | 1548.27 |
|  | 1110.39 | 830.74 | 1239.80 | 1096.29 |
|  | 1599.40 | 979.30 | 133.06 | 879.35 |
|  | 998.02 | 767.61 | 1368.99 | 1805.40 |
|  | 1643.17 | 1437.80 | 1035.89 | 1097.55 |
|  | 1405.40 | 1745.64 | 1436.75 | 1876.74 |
|  | 1367.73 | 1636.86 | 1555.00 | 2118.72 |
|  | 1548.69 | 1682.94 | 1131.85 | 1439.06 |
|  | 1673.26 | 962.67 | 1469.57 | 1484.30 |
|  | 1007.07 | 1849.80 | 874.51 | 1070.62 |
|  | 1395.51 | 1867.06 | 1594.14 | 2888.44 |
|  | 1564.47 | 1853.38 | 1209.07 | 1141.95 |
|  | 1328.80 | 1949.33 | 1901.78 | 1330.70 |
|  | 1645.27 | 2602.26 | 1572.05 | 1375.73 |
|  | 1497.14 | 1256.84 | 1621.08 | 1456.53 |
|  | 1144.05 | 2485.06 | 1725.65 | 846.73 |
|  | 1512.92 | 1481.36 | 1823.08 | 842.52 |
|  | 3850.69 | 1382.67 | 1401.40 | 825.06 |
|  | 1222.96 | 1448.95 | 1095.45 | 1256.00 |
|  | 955.94 | 1473.99 | 1457.37 | 1222.96 |
|  | 1203.39 | 1625.07 | 1240.43 | 1160.89 |
|  | 1008.96 | 1546.17 | 950.89 | 968.56 |
|  | 1029.38 | 1555.00 | 796.65 | 1893.15 |
|  | 1374.04 | 1221.07 | 996.55 | 674.82 |
|  | 854.73 | 2009.72 | 1032.53 | 1686.94 |
|  | 800.02 | 1679.99 | 646.62 | 2173.85 |
|  | 1142.16 | 1589.72 | 1181.51 | 1354.89 |
| IIa | 1140.06 | 1130.80 | 914.28 | 955.94 |
|  | 715.01 | 1477.15 | 1277.25 | 884.19 |
|  | 446.30 | 1562.79 | 914.49 | 1207.81 |
|  | 676.50 | 1730.92 | 874.51 | 1109.55 |
|  | 680.50 | 823.16 | 842.52 | 602.43 |
|  | 726.37 | 1379.09 | 999.29 | 1144.90 |
|  | 857.88 | 1175.62 | 520.37 | 1186.98 |
|  | 311.42 | 1300.40 | 1329.85 | 1337.22 |
|  | 885.45 | 1573.10 | 770.35 | 1435.49 |
|  | 862.30 | 1426.44 | 826.32 | 1161.52 |
|  | 731.63 | 1241.06 | 818.96 | 1152.05 |
|  | 716.90 | 1777.21 | 1460.95 | 1224.43 |
|  | 893.86 | 837.26 | 909.86 | 1166.99 |
|  | 706.38 | 1086.82 | 600.96 | 850.52 |
|  | 856.20 | 1703.98 | 1928.08 | 1198.55 |
|  | 854.52 | 1107.02 | 620.32 | 1065.99 |
|  | 807.38 | 759.20 | 1203.81 | 819.38 |
|  | 964.99 | 1575.42 | 1383.93 | 1284.61 |
|  | 990.45 | 1351.53 | 1051.05 | 690.60 |
|  | 1420.34 | 1286.72 | 1466.00 | 1098.39 |
|  | 954.89 | 828.42 | 1150.79 | 818.32 |
|  | 1294.50 | 1251.58 | 1262.10 | 1063.46 |
|  | 842.94 | 1166.99 | 1126.17 | 1101.13 |
|  | 552.14 | 938.89 | 1165.31 | 1361.84 |
|  | 633.36 | 939.10 | 1237.27 | 1139.64 |
|  | 954.68 | 1532.49 | 951.10 | 951.94 |
|  | 1040.95 | 1485.57 | 1085.14 | 1504.92 |
|  | 828.42 | 1152.68 | 1064.52 | 713.32 |
|  | 1126.59 | 858.30 | 621.37 | 774.98 |
|  | 580.97 | 1698.51 | 1096.71 | 1297.87 |
| IIx | 470.29 | 1628.86 | 665.35 | 935.95 |
|  | 437.88 | 732.89 | 952.15 | 1509.97 |
|  | 879.98 | 1393.61 | 954.04 | 648.94 |
|  | 614.43 | 964.99 | 745.31 | 1334.69 |
|  | 2264.96 | 1181.51 | 892.18 | 1449.37 |
|  | 950.05 | 1174.56 | 1043.05 | 1162.36 |
|  | 1508.50 | 1630.33 | 798.54 | 1179.40 |
|  | 1512.50 | 1029.80 | 795.81 | 1351.32 |
|  | 1760.58 | 1272.41 | 1177.93 | 1040.11 |
|  | 1865.16 | 1269.67 | 1117.96 | 977.82 |
|  | 1317.86 | 1559.84 | 936.16 | 1501.98 |
|  | 1275.15 | 1299.98 | 1193.92 | 1252.00 |
|  | 822.95 | 1063.88 | 564.14 | 883.76 |
|  |  | 1273.88 | 867.77 | 676.08 |
|  |  | 1357.42 | 1070.83 | 733.31 |
|  |  | 1005.60 | 601.38 | 533.41 |
|  |  | 1176.25 | 1020.22 | 1199.60 |
|  |  | 1209.07 | 1408.76 | 1296.82 |
|  |  | 1102.81 | 1011.91 | 1628.86 |
|  |  | 1646.54 | 1079.46 | 744.89 |
|  |  | 1372.99 | 652.09 | 1021.38 |
|  |  | 577.81 | 1920.50 | 1148.89 |
|  |  | 992.76 | 1194.98 | 766.14 |
|  |  | 995.07 | 388.23 | 604.33 |
|  |  | 654.62 | 1055.26 | 871.35 |
|  |  | 1229.48 | 482.70 | 1098.81 |
|  |  | 1349.00 | 494.70 | 487.12 |
|  |  |  | 444.83 |  |
|  |  |  | 376.65 |  |
|  |  |  |  |  |
|  | Disuse |  |  |  |
| Type | Dis1 | Dis2 | Dis3 | Dis4 |
| I | 1316.18 | 848.83 | 1476.31 | 858.93 |
|  | 1028.06 | 970.25 | 1862.64 | 642.41 |
|  | 1686.73 | 755.83 | 1509.34 | 1049.79 |
|  | 1133.95 | 1122.80 | 1595.62 | 1057.36 |
|  | 981.19 | 1144.05 | 1598.77 | 889.66 |
|  | 1128.27 | 845.89 | 1672.00 | 1219.17 |
|  | 991.50 | 945.21 | 1334.06 | 774.56 |
|  | 963.51 | 823.79 | 1411.29 | 777.71 |
|  | 1369.83 | 731.21 | 1100.50 | 703.01 |
|  | 699.86 | 997.81 | 1488.09 | 970.88 |
|  | 1338.90 | 797.91 | 1487.88 | 830.95 |
|  | 1443.90 | 630.00 | 854.31 | 725.11 |
|  | 1003.49 | 795.81 | 1359.10 | 1242.95 |
|  | 792.23 | 946.47 | 1500.08 | 925.64 |
|  | 1349.21 | 868.47 | 1379.93 | 924.59 |
|  | 517.42 | 1335.54 | 1403.08 | 1121.96 |
|  | 1051.68 | 717.32 | 1254.52 | 832.42 |
|  | 940.16 | 1109.33 | 1506.82 | 732.05 |
|  | 1575.63 | 953.41 | 1324.59 | 780.87 |
|  | 1327.75 | 970.88 | 1163.41 | 729.95 |
|  | 1259.15 | 640.31 | 1521.76 | 515.53 |
|  | 722.58 | 409.90 | 1767.11 | 853.25 |
|  | 798.97 | 814.96 | 1244.63 | 1029.38 |
|  | 831.58 | 955.73 | 1734.49 | 689.34 |
|  | 1889.57 | 701.75 | 1176.88 | 986.45 |
|  | 1034.21 | 1000.97 | 1376.57 | 1105.97 |
|  | 1017.17 | 640.10 | 1311.97 | 1114.17 |
|  | 984.77 | 713.75 | 1196.87 | 861.88 |
|  | 875.56 | 672.08 | 1914.40 | 1291.77 |
|  | 869.25 | 905.65 | 1360.37 | 789.08 |
| IIa | 671.45 | 875.98 | 1074.19 | 704.28 |
|  | 1019.06 | 654.62 | 892.18 | 1014.01 |
|  | 1163.83 | 1173.09 | 779.19 | 510.48 |
|  | 1339.74 | 704.91 | 1030.64 | 526.89 |
|  | 1245.69 | 712.48 | 937.63 | 862.30 |
|  | 878.92 | 609.59 | 1460.74 | 1007.70 |
|  | 1376.36 | 1216.23 | 763.83 | 1187.82 |
|  | 1593.09 | 1053.57 | 1042.42 | 596.12 |
|  | 946.63 | 628.53 | 1023.69 | 1336.80 |
|  | 1519.02 | 828.21 | 1446.43 | 982.66 |
|  | 900.18 | 1221.91 | 1491.46 | 1197.08 |
|  | 964.14 | 783.60 | 908.59 | 514.90 |
|  | 1109.55 | 1192.87 | 1322.07 | 990.24 |
|  | 934.05 | 1051.05 | 1091.45 | 592.12 |
|  | 879.56 | 1133.32 | 890.29 | 582.44 |
|  | 995.71 | 643.46 | 1290.93 | 760.46 |
|  | 438.52 | 922.48 | 2098.73 | 766.98 |
|  | 494.49 | 966.55 | 1257.89 | 877.66 |
|  | 973.82 | 709.54 | 1266.52 | 1019.70 |
|  | 677.13 | 752.25 | 1267.15 | 1387.93 |
|  | 889.24 | 455.14 | 923.74 | 741.52 |
|  | 1382.25 | 796.86 | 1322.09 | 719.01 |
|  | 824.85 | 753.51 | 1114.38 | 515.74 |
|  | 621.58 | 630.21 | 981.82 | 913.22 |
|  | 911.54 | 1142.58 | 1238.74 | 909.23 |
|  | 590.86 | 714.59 | 653.14 | 532.78 |
|  | 563.93 | 522.26 | 1706.51 | 942.89 |
|  | 596.07 | 697.33 | 1706.93 | 1092.08 |
|  | 1192.16 | 1137.53 | 818.96 | 736.68 |
|  | 1029.16 | 487.96 | 896.39 | 765.93 |
| IIx | 1000.55 | 1065.78 | 1620.02 | 889.87 |
|  | 1138.16 | 933.21 | 1582.78 | 1266.52 |
|  | 594.23 | 1122.80 | 1945.54 | 766.56 |
|  | 1604.87 | 1309.23 | 2757.76 | 394.75 |
|  | 989.18 | 1467.89 | 2185.42 | 411.16 |
|  | 493.22 | 1005.60 | 1205.29 | 1154.24 |
|  | 1434.01 | 1016.75 | 1246.74 | 629.58 |
|  | 1533.12 | 1272.94 | 3256.25 | 737.31 |
|  | 1637.28 | 1261.68 | 1308.60 | 1157.10 |
|  | 1267.99 | 1472.52 | 1624.02 | 1036.11 |
|  | 430.52 | 1284.61 | 1824.34 | 895.34 |
|  | 805.28 | 1261.68 | 2391.21 | 1270.10 |
|  | 1482.20 | 1206.97 | 2197.63 | 627.68 |
|  | 980.35 | 1189.93 | 2836.04 | 1224.43 |
|  | 522.47 | 1121.33 | 2320.09 | 1095.03 |
|  | 817.48 | 1095.24 | 1799.09 | 1010.65 |
|  | 1787.10 | 505.43 | 1833.18 | 836.42 |
|  | 955.52 | 547.72 | 1565.74 | 1197.29 |
|  | 1555.43 | 1529.33 | 1379.09 | 914.49 |
|  | 1228.43 | 772.24 | 1708.61 | 1409.60 |
|  | 1230.54 | 1069.57 | 2156.60 | 1210.13 |
|  | 1276.20 | 1007.91 | 1483.04 | 846.94 |
|  | 1285.04 | 452.40 | 1824.97 | 1438.22 |
|  | 472.81 | 1200.45 | 1681.26 | 1104.71 |
|  | 1496.72 | 1245.27 | 1923.87 | 1305.45 |
|  | 1271.15 | 1398.87 | 1329.01 | 1170.57 |
|  | 882.50 | 792.23 | 1959.22 | 567.50 |
|  | 1368.78 | 909.23 | 2167.12 | 1121.96 |
|  | 1138.79 | 1299.34 | 1894.83 | 1363.73 |
|  | 1775.31 | 1163.62 | 2239.29 | 1220.86 |
|  | Reloading |  |  |  |
| Type | Re1 | Re2 | Re3 | Re4 |
| I | 704.91 | 1050.42 | 947.10 | 1013.38 |
|  | 594.02 | 1025.38 | 596.75 | 829.06 |
|  | 756.46 | 760.25 | 718.58 | 1071.67 |
|  | 781.08 | 1042.21 | 978.03 | 731.84 |
|  | 627.05 | 1076.72 | 952.15 | 928.37 |
|  | 782.34 | 1227.38 | 994.45 | 1098.81 |
|  | 620.95 | 1998.15 | 810.33 | 1335.33 |
|  | 603.49 | 966.46 | 1319.75 | 902.49 |
|  | 742.99 | 974.67 | 1181.93 | 1152.89 |
|  | 797.91 | 1245.27 | 1161.10 | 1005.60 |
|  | 819.80 | 911.75 | 1036.32 | 994.45 |
|  | 704.70 | 691.65 | 1188.03 | 853.25 |
|  | 858.93 | 749.10 | 899.97 | 1405.82 |
|  | 441.04 | 1154.36 | 1112.28 | 915.12 |
|  | 598.22 | 1175.41 | 1093.97 | 865.88 |
|  | 717.11 | 810.12 | 974.67 | 966.88 |
|  | 367.60 | 854.31 | 1128.90 | 1729.65 |
|  | 394.75 | 1051.47 | 1049.58 | 1685.89 |
|  | 1052.10 | 1278.51 | 1059.25 | 1050.42 |
|  | 764.25 | 955.10 | 926.69 | 1025.80 |
|  | 836.42 | 1042.00 | 910.07 | 868.19 |
|  | 415.79 | 1074.83 | 1126.59 | 1282.30 |
|  | 1142.37 | 902.28 | 522.68 | 1276.20 |
|  | 1335.54 | 984.56 | 744.68 | 1255.58 |
|  | 681.76 | 1145.11 | 875.56 | 758.78 |
|  | 929.85 | 744.05 | 929.22 | 563.51 |
|  | 797.70 | 1344.37 | 734.16 | 919.54 |
|  | 1144.26 | 1769.00 | 642.62 | 772.66 |
|  | 1123.43 | 1578.57 | 887.76 | 842.94 |
|  | 750.57 | 1198.76 | 971.93 | 764.25 |
| IIa | 585.39 | 2380.27 | 967.51 | 636.73 |
|  | 692.07 | 1400.56 | 978.66 | 1069.78 |
|  | 1190.56 | 1299.76 | 1400.35 | 749.10 |
|  | 1186.35 | 1214.54 | 1252.00 | 966.46 |
|  | 950.68 | 1111.02 | 1123.01 | 1034.85 |
|  | 575.29 | 1612.03 | 1196.87 | 915.96 |
|  | 627.47 | 1620.44 | 58.29 | 483.55 |
|  | 819.59 | 1037.58 | 1458.21 | 788.87 |
|  | 588.97 | 1318.49 | 1189.72 | 836.21 |
|  | 634.42 | 786.34 | 1267.15 | 588.33 |
|  | 840.21 | 1078.19 | 1369.20 | 1039.05 |
|  | 621.37 | 1411.71 | 1029.16 | 807.59 |
|  | 774.98 | 1062.83 | 1001.39 | 607.90 |
|  | 468.82 | 1254.73 | 975.72 | 688.71 |
|  | 554.46 | 872.19 | 1090.19 | 1152.47 |
|  | 747.41 | 1018.01 | 1107.23 | 644.94 |
|  | 784.03 | 1215.81 | 2150.70 | 774.98 |
|  | 764.67 | 1879.89 | 846.31 | 1092.92 |
|  | 874.09 | 1485.36 | 1734.28 | 1062.41 |
|  | 440.62 | 1107.02 | 1345.85 | 436.62 |
|  | 433.04 | 904.18 | 1078.40 | 605.17 |
|  | 536.15 | 1583.62 | 1110.60 | 704.28 |
|  | 701.12 | 1127.64 | 1247.79 | 301.74 |
|  | 781.71 | 932.16 | 1127.01 | 655.67 |
|  | 588.76 | 1367.52 | 2349.76 | 358.56 |
|  | 483.12 | 1418.02 | 862.51 | 559.93 |
|  | 1015.28 | 1278.30 | 1163.31 | 863.14 |
|  | 485.65 | 1209.71 | 1033.37 | 837.89 |
|  | 956.78 | 1261.47 | 721.95 | 1046.00 |
|  | 654.20 | 1513.55 | 922.90 | 543.73 |
| IIx | 713.75 | 1065.78 | 1620.02 | 889.87 |
|  | 1132.69 | 933.21 | 1582.78 | 1266.52 |
|  | 1237.90 | 1122.80 | 1945.54 | 766.56 |
|  | 1169.09 | 1309.23 | 2757.76 | 394.75 |
|  | 1011.07 | 1467.89 | 2185.42 | 411.16 |
|  | 959.52 | 1005.60 | 1205.29 | 154.24 |
|  | 1260.00 | 1016.75 | 1246.74 | 629.58 |
|  | 1269.25 | 1272.94 | 3256.25 | 737.31 |
|  | 916.38 | 1261.68 | 1308.60 | 1157.10 |
|  | 1138.37 | 1472.52 | 1624.02 | 1036.11 |
|  | 1301.87 | 1284.61 | 1824.34 | 895.34 |
|  | 1378.25 | 1261.68 | 2391.21 | 1270.10 |
|  | 1247.16 | 1206.97 | 2197.63 | 627.68 |
|  | 1533.33 | 1189.93 | 2836.04 | 1224.43 |
|  | 1333.43 | 1121.33 | 2320.09 | 1095.03 |
|  | 1308.18 | 1095.24 | 1799.09 | 1010.65 |
|  | 1505.35 | 505.43 | 1833.18 | 836.42 |
|  | 769.30 | 547.72 | 1565.74 | 1197.29 |
|  | 1162.99 | 1529.33 | 1379.09 | 914.49 |
|  | 1079.46 | 772.24 | 1708.61 | 1409.60 |
|  | 864.83 | 1069.57 | 2156.60 | 1210.13 |
|  | 1126.17 | 1007.91 | 1483.04 | 846.94 |
|  | 1100.29 | 452.40 | 1824.97 | 1438.22 |
|  | 770.35 | 1200.45 | 1681.26 | 1104.71 |
|  | 802.96 | 1245.27 | 1923.87 | 1305.45 |
|  | 912.38 | 1398.87 | 1329.01 | 1170.57 |
|  | 1105.97 | 792.23 | 1959.22 | 567.50 |
|  | 1136.48 | 909.23 | 2167.12 | 1121.96 |
|  | 1010.02 | 1299.34 | 1894.83 | 1363.73 |
|  | 1428.33 | 1163.62 | 2239.29 | 1220.86 |
| Minimal Feret diameter (µm) | | | | |
|  | Control |  |  |  |
| Type | Con1 | Con2 | Con3 | Con4 |
| I | 23.13 | 37.70 | 30.29 | 27.98 |
|  | 29.90 | 37.22 | 35.90 | 27.06 |
|  | 31.17 | 30.54 | 29.44 | 23.22 |
|  | 30.70 | 36.18 | 38.87 | 36.36 |
|  | 27.13 | 25.88 | 32.42 | 36.51 |
|  | 27.97 | 24.23 | 33.83 | 33.57 |
|  | 33.22 | 30.57 | 30.30 | 43.87 |
|  | 40.63 | 31.07 | 29.31 | 26.27 |
|  | 35.75 | 39.65 | 37.84 | 35.52 |
|  | 29.66 | 40.33 | 36.26 | 47.81 |
|  | 34.14 | 34.17 | 26.79 | 39.84 |
|  | 27.10 | 31.45 | 37.86 | 32.33 |
|  | 32.07 | 37.70 | 30.58 | 31.54 |
|  | 40.02 | 38.88 | 30.98 | 49.04 |
|  | 36.26 | 42.88 | 33.58 | 35.38 |
|  | 34.74 | 43.02 | 31.91 | 39.62 |
|  | 32.32 | 49.58 | 38.86 | 38.03 |
|  | 35.02 | 35.50 | 31.75 | 35.90 |
|  | 27.62 | 51.76 | 40.01 | 30.29 |
|  | 37.75 | 31.27 | 35.68 | 25.06 |
|  | 56.28 | 39.55 | 29.86 | 28.18 |
|  | 29.68 | 38.84 | 29.75 | 39.16 |
|  | 24.96 | 41.10 | 39.56 | 36.11 |
|  | 27.12 | 40.17 | 40.48 | 27.68 |
|  | 28.43 | 42.91 | 30.90 | 24.21 |
|  | 34.06 | 40.08 | 30.72 | 43.36 |
|  | 33.71 | 30.70 | 30.05 | 26.12 |
|  | 21.42 | 42.69 | 27.40 | 42.24 |
|  | 21.91 | 34.03 | 22.88 | 48.20 |
|  | 30.97 | 32.01 | 35.54 | 42.00 |
| IIa | 33.97 | 37.57 | 30.40 | 23.18 |
|  | 20.92 | 36.97 | 20.56 | 21.49 |
|  | 20.23 | 34.01 | 28.25 | 30.68 |
|  | 21.07 | 37.47 | 21.95 | 33.51 |
|  | 23.92 | 32.72 | 26.13 | 21.97 |
|  | 26.97 | 40.09 | 29.84 | 33.15 |
|  | 24.75 | 33.69 | 21.94 | 34.58 |
|  | 18.57 | 34.89 | 35.98 | 27.39 |
|  | 28.65 | 42.31 | 25.43 | 37.77 |
|  | 25.97 | 33.94 | 25.64 | 33.06 |
|  | 28.16 | 40.58 | 20.33 | 33.43 |
|  | 26.55 | 42.55 | 27.60 | 33.57 |
|  | 25.02 | 26.47 | 26.52 | 34.25 |
|  | 22.98 | 31.39 | 23.81 | 31.55 |
|  | 27.29 | 30.02 | 31.76 | 37.09 |
|  | 23.81 | 25.00 | 27.49 | 33.95 |
|  | 30.77 | 27.48 | 34.74 | 31.63 |
|  | 29.63 | 38.53 | 30.48 | 30.31 |
|  | 33.02 | 36.33 | 34.07 | 20.80 |
|  | 32.14 | 35.43 | 36.53 | 32.93 |
|  | 24.11 | 27.65 | 25.28 | 21.98 |
|  | 34.93 | 30.06 | 29.20 | 25.92 |
|  | 25.31 | 29.97 | 35.78 | 34.34 |
|  | 20.64 | 30.60 | 33.39 | 36.31 |
|  | 26.97 | 25.44 | 36.24 | 27.80 |
|  | 31.30 | 33.03 | 28.96 | 32.87 |
|  | 28.07 | 35.61 | 26.30 | 35.15 |
|  | 20.50 | 28.87 | 29.71 | 28.59 |
|  | 31.13 | 28.47 | 23.40 | 24.92 |
|  | 23.46 | 35.19 | 28.46 | 37.11 |
| IIx | 17.44 | 39.51 | 28.88 | 20.85 |
|  | 16.33 | 26.02 | 31.73 | 30.60 |
|  | 25.11 | 39.57 | 30.33 | 20.06 |
|  | 22.86 | 20.72 | 26.35 | 30.45 |
|  | 39.21 | 33.77 | 30.07 | 34.65 |
|  | 25.66 | 34.89 | 30.07 | 30.70 |
|  | 28.62 | 32.38 | 27.63 | 26.31 |
|  | 28.65 | 34.65 | 25.34 | 28.55 |
|  | 39.93 | 38.47 | 34.73 | 31.19 |
|  | 36.16 | 26.19 | 35.61 | 24.92 |
|  | 30.31 | 38.53 | 24.47 | 42.22 |
|  | 25.92 | 34.64 | 33.59 | 37.96 |
|  | 29.23 | 36.67 | 18.92 | 22.45 |
|  |  | 39.84 | 27.87 | 21.76 |
|  |  | 38.53 | 27.90 | 27.27 |
|  |  | 32.49 | 15.72 | 20.04 |
|  |  | 29.74 | 29.53 | 38.36 |
|  |  | 39.75 | 27.41 | 31.00 |
|  |  | 29.43 | 23.57 | 38.05 |
|  |  | 42.17 | 28.11 | 25.00 |
|  |  | 32.31 | 17.49 | 33.37 |
|  |  | 18.77 | 40.35 | 34.37 |
|  |  | 30.41 | 30.13 | 30.37 |
|  |  | 34.86 | 18.17 | 18.38 |
|  |  | 29.20 | 29.45 | 20.75 |
|  |  | 40.78 | 19.80 | 31.17 |
|  |  | 31.23 | 11.17 | 19.40 |
|  |  |  | 18.44 |  |
|  |  |  | 17.07 |  |
|  |  |  |  |  |
|  | Disuse |  |  |  |
| Type | Dis1 | Dis2 | Dis3 | Dis4 |
| I | 28.34 | 26.07 | 35.78 | 23.63 |
|  | 28.90 | 20.79 | 32.38 | 23.47 |
|  | 41.07 | 24.17 | 31.80 | 28.28 |
|  | 29.88 | 31.95 | 36.85 | 30.64 |
|  | 31.92 | 34.86 | 44.04 | 28.17 |
|  | 32.82 | 26.34 | 28.00 | 30.35 |
|  | 29.18 | 29.17 | 31.33 | 28.29 |
|  | 27.01 | 29.17 | 31.98 | 27.23 |
|  | 39.56 | 21.81 | 31.02 | 24.65 |
|  | 22.93 | 30.71 | 39.79 | 30.38 |
|  | 36.94 | 21.97 | 34.19 | 19.18 |
|  | 38.66 | 26.67 | 31.96 | 27.90 |
|  | 27.12 | 28.90 | 34.96 | 31.66 |
|  | 29.36 | 24.51 | 33.75 | 29.47 |
|  | 32.73 | 27.93 | 35.79 | 26.37 |
|  | 22.04 | 30.02 | 33.44 | 36.05 |
|  | 34.94 | 27.02 | 33.03 | 26.62 |
|  | 28.38 | 28.53 | 37.78 | 22.42 |
|  | 38.62 | 27.90 | 29.68 | 25.42 |
|  | 36.33 | 27.31 | 36.18 | 20.70 |
|  | 36.21 | 25.59 | 31.90 | 19.04 |
|  | 26.94 | 21.76 | 31.87 | 24.28 |
|  | 28.15 | 23.51 | 35.21 | 32.78 |
|  | 25.28 | 31.32 | 40.87 | 26.87 |
|  | 40.74 | 28.79 | 26.89 | 22.87 |
|  | 23.89 | 33.77 | 39.29 | 26.81 |
|  | 29.86 | 27.43 | 32.92 | 34.74 |
|  | 26.17 | 27.21 | 28.97 | 27.08 |
|  | 24.01 | 26.50 | 43.40 | 29.47 |
|  | 28.08 | 29.00 | 35.09 | 31.18 |
| IIa | 22.23 | 23.33 | 30.46 | 28.01 |
|  | 20.75 | 17.81 | 23.46 | 32.17 |
|  | 31.44 | 29.75 | 20.24 | 18.87 |
|  | 38.69 | 20.77 | 32.05 | 16.05 |
|  | 32.56 | 24.09 | 28.22 | 27.25 |
|  | 26.45 | 25.25 | 33.72 | 34.31 |
|  | 35.32 | 30.48 | 25.90 | 26.66 |
|  | 39.30 | 30.99 | 32.60 | 19.95 |
|  | 26.07 | 27.61 | 29.55 | 37.10 |
|  | 36.90 | 26.38 | 40.04 | 32.48 |
|  | 23.14 | 32.95 | 42.77 | 29.82 |
|  | 35.12 | 26.85 | 31.22 | 20.94 |
|  | 25.24 | 36.18 | 22.23 | 30.54 |
|  | 31.93 | 28.69 | 33.86 | 17.08 |
|  | 34.30 | 27.98 | 28.07 | 24.01 |
|  | 33.82 | 27.08 | 31.90 | 26.48 |
|  | 22.34 | 25.18 | 35.82 | 25.83 |
|  | 22.94 | 28.99 | 31.12 | 20.77 |
|  | 31.52 | 27.05 | 32.69 | 27.50 |
|  | 22.94 | 23.01 | 34.94 | 30.39 |
|  | 28.34 | 21.46 | 27.36 | 25.95 |
|  | 36.52 | 22.44 | 32.19 | 28.93 |
|  | 23.81 | 28.22 | 35.69 | 22.91 |
|  | 20.97 | 21.99 | 26.91 | 29.59 |
|  | 34.36 | 35.31 | 31.42 | 30.99 |
|  | 25.04 | 23.16 | 21.72 | 23.78 |
|  | 19.47 | 18.81 | 42.07 | 33.97 |
|  | 23.97 | 22.84 | 39.77 | 33.10 |
|  | 31.83 | 30.48 | 25.56 | 18.57 |
|  | 22.38 | 17.11 | 26.97 | 25.06 |
| IIx | 31.35 | 26.57 | 32.76 | 29.15 |
|  | 25.32 | 19.97 | 37.86 | 33.93 |
|  | 24.48 | 24.14 | 42.62 | 27.02 |
|  | 26.84 | 33.39 | 35.82 | 21.29 |
|  | 31.52 | 27.06 | 41.50 | 19.42 |
|  | 17.36 | 25.37 | 27.73 | 12.61 |
|  | 30.13 | 30.37 | 26.46 | 24.67 |
|  | 36.06 | 31.61 | 38.71 | 19.69 |
|  | 34.99 | 30.28 | 28.99 | 30.69 |
|  | 24.77 | 36.68 | 39.11 | 30.66 |
|  | 19.14 | 32.21 | 31.64 | 29.30 |
|  | 27.49 | 33.55 | 31.73 | 29.75 |
|  | 33.88 | 29.66 | 39.53 | 15.45 |
|  | 28.88 | 36.49 | 47.59 | 35.31 |
|  | 24.34 | 36.63 | 43.43 | 26.73 |
|  | 27.90 | 25.28 | 35.44 | 28.47 |
|  | 43.64 | 21.42 | 40.27 | 30.08 |
|  | 24.06 | 24.75 | 29.80 | 27.89 |
|  | 32.83 | 42.08 | 39.63 | 23.39 |
|  | 34.42 | 16.25 | 35.17 | 41.07 |
|  | 37.61 | 32.48 | 39.81 | 29.69 |
|  | 32.56 | 31.78 | 35.11 | 26.88 |
|  | 31.08 | 19.91 | 40.75 | 37.75 |
|  | 19.45 | 35.12 | 34.07 | 32.55 |
|  | 36.11 | 31.65 | 45.59 | 35.72 |
|  | 35.40 | 27.80 | 36.37 | 36.12 |
|  | 31.77 | 26.49 | 40.93 | 26.76 |
|  | 31.55 | 31.11 | 45.07 | 33.22 |
|  | 25.14 | 39.55 | 32.73 | 34.16 |
|  | 47.09 | 28.64 | 39.74 | 34.03 |
|  | Reloading |  |  |  |
| Type | Re1 | Re2 | Re3 | Re4 |
| I | 24.11 | 32.66 | 29.27 | 27.08 |
|  | 16.85 | 32.80 | 25.74 | 28.23 |
|  | 22.40 | 24.48 | 26.12 | 32.94 |
|  | 27.49 | 31.53 | 26.12 | 18.88 |
|  | 24.78 | 37.21 | 24.10 | 28.35 |
|  | 22.15 | 30.31 | 26.66 | 27.93 |
|  | 25.70 | 40.39 | 23.90 | 38.39 |
|  | 23.91 | 28.51 | 28.32 | 31.26 |
|  | 27.24 | 27.64 | 20.52 | 32.59 |
|  | 29.99 | 35.62 | 33.22 | 32.48 |
|  | 29.07 | 31.03 | 28.09 | 30.43 |
|  | 28.40 | 24.31 | 32.11 | 27.07 |
|  | 31.91 | 19.76 | 30.39 | 35.10 |
|  | 22.36 | 34.09 | 32.83 | 26.65 |
|  | 23.90 | 37.03 | 33.69 | 31.63 |
|  | 28.37 | 24.68 | 33.18 | 32.65 |
|  | 20.50 | 28.82 | 30.49 | 41.47 |
|  | 17.51 | 31.79 | 29.03 | 39.97 |
|  | 31.19 | 31.85 | 35.05 | 34.81 |
|  | 29.18 | 27.77 | 31.76 | 29.97 |
|  | 27.96 | 30.10 | 45.94 | 23.63 |
|  | 20.11 | 31.25 | 33.44 | 25.47 |
|  | 30.86 | 23.50 | 25.66 | 38.57 |
|  | 38.36 | 24.78 | 29.17 | 34.55 |
|  | 20.11 | 34.73 | 28.94 | 26.27 |
|  | 31.37 | 28.75 | 30.91 | 24.81 |
|  | 28.29 | 33.75 | 27.88 | 27.96 |
|  | 31.65 | 43.22 | 24.84 | 29.23 |
|  | 28.41 | 32.72 | 28.75 | 25.33 |
|  | 27.84 | 27.09 | 31.64 | 23.70 |
| IIa | 25.27 | 51.23 | 28.83 | 19.88 |
|  | 27.14 | 38.27 | 26.50 | 22.55 |
|  | 34.36 | 32.41 | 38.87 | 19.85 |
|  | 27.99 | 27.77 | 29.76 | 27.52 |
|  | 29.99 | 24.42 | 34.54 | 28.90 |
|  | 26.36 | 34.27 | 31.70 | 30.63 |
|  | 25.95 | 36.25 | 24.10 | 23.38 |
|  | 30.96 | 21.77 | 39.87 | 30.73 |
|  | 23.86 | 38.68 | 37.90 | 25.84 |
|  | 27.61 | 28.07 | 33.05 | 22.88 |
|  | 25.42 | 33.51 | 38.93 | 32.25 |
|  | 25.51 | 32.09 | 27.41 | 27.85 |
|  | 21.31 | 35.05 | 32.49 | 26.27 |
|  | 20.08 | 32.27 | 31.81 | 23.98 |
|  | 24.27 | 26.18 | 28.48 | 31.56 |
|  | 24.42 | 31.74 | 35.70 | 18.63 |
|  | 20.36 | 24.58 | 37.63 | 21.15 |
|  | 26.84 | 35.87 | 31.25 | 27.76 |
|  | 33.39 | 30.00 | 42.79 | 29.01 |
|  | 20.65 | 27.80 | 36.49 | 21.42 |
|  | 22.34 | 30.14 | 35.01 | 17.92 |
|  | 17.21 | 38.16 | 36.28 | 26.09 |
|  | 23.03 | 33.64 | 33.31 | 17.43 |
|  | 23.58 | 30.15 | 32.29 | 22.62 |
|  | 25.83 | 26.38 | 35.80 | 13.41 |
|  | 18.71 | 31.18 | 29.20 | 24.78 |
|  | 27.96 | 34.87 | 27.31 | 19.73 |
|  | 23.78 | 29.49 | 32.59 | 21.90 |
|  | 25.77 | 34.23 | 22.87 | 30.73 |
|  | 29.18 | 40.49 | 32.51 | 20.98 |
| IIx | 29.51 | 29.91 | 29.44 | 37.54 |
|  | 32.26 | 35.31 | 28.44 | 40.76 |
|  | 27.81 | 35.77 | 29.01 | 42.26 |
|  | 30.44 | 30.14 | 24.75 | 30.08 |
|  | 22.63 | 36.19 | 36.52 | 28.71 |
|  | 27.10 | 34.95 | 37.29 | 32.19 |
|  | 30.75 | 35.86 | 43.48 | 25.07 |
|  | 29.23 | 28.44 | 29.34 | 32.82 |
|  | 34.28 | 33.26 | 32.43 | 32.64 |
|  | 30.99 | 26.19 | 38.11 | 15.95 |
|  | 39.99 | 34.73 | 25.70 | 16.48 |
|  | 40.08 | 34.76 | 38.22 | 24.79 |
|  | 34.13 | 30.54 | 23.10 | 29.37 |
|  | 39.61 | 25.85 | 30.03 | 22.09 |
|  | 39.24 | 30.70 | 26.20 | 24.77 |
|  | 37.18 | 34.79 | 28.97 | 29.21 |
|  | 37.33 | 26.73 | 27.62 | 35.35 |
|  | 28.45 | 23.07 | 31.64 | 28.61 |
|  | 34.06 | 21.93 | 30.50 | 25.33 |
|  | 27.19 | 22.42 | 23.39 | 17.45 |
|  | 30.54 | 29.00 | 33.50 | 23.03 |
|  | 34.69 | 34.75 | 27.54 | 26.47 |
|  | 26.80 | 39.54 | 28.00 | 26.87 |
|  | 23.74 | 25.67 | 28.80 | 31.39 |
|  | 25.49 | 19.71 | 33.62 | 31.77 |
|  | 24.90 | 29.05 | 26.57 | 24.07 |
|  | 33.32 | 36.75 | 23.60 | 19.54 |
|  | 35.83 | 21.50 | 29.70 | 29.79 |
|  | 22.53 | 25.23 | 28.55 | 17.69 |
|  | 35.33 | 31.35 | 40.45 | 25.97 |
