# Supplementary material for: Distribution of myogenic stem cell activator, hepatocyte growth factor, in skeletal muscle extracellular matrix and effect of short-term disuse and reloading
Source: PLoS One. 2025 Sep 3;20(9):e0321839. doi: 10.1371/journal.pone.0321839 (PMC12407438; doi:10.1371/journal.pone.0321839)
Supplement: S5 Table — This is the table of data in Fig 2d. Muscle fiber types were identified using co-staining images of anti-MyHC and anti-Laminin antibodies, and the number of each type was counted. Proportion was obtained for each skeletal muscle by dividing the number of each muscle fiber type by the total number of muscle fibers. (DOCX) [file pone.0321839.s010.docx]

**S5 Table. Proportions of muscle fiber types in the Sol for the control, disuse, and reloading groups (n=4).**

|  | Control (%) | Disuse (%) | Reloading(%) |
| --- | --- | --- | --- |
| Type | ave±s.e. | ave±s.e. | ave±s.e. |
| I | 35.48±4.72 | 30.34±0.50 | 33.68±1.68 |
| IIa | 56.63±5.20 | 57.99±2.28 | 49.49±3.12 |
| IIx | 7.89±0.59 | 11.67±2.26 | 16.84±2.08 |
| t-test / Bonferroni correction: α = 0.0133 (0.05/3) | | | |
|  | Con:Dis | Dis:Re | Con:Re |
| Type | t-test | t-test | t-test |
| I | 0.3207 | 0.1049 | 0.7388 |
| IIa | 0.8191 | 0.0701 | 0.2829 |
| IIx | 0.1563 | 0.1435 | 0.0061 |

Each data

|  | Control |  |  |  |
| --- | --- | --- | --- | --- |
| Type | Con1 | Con2 | Con3 | Con4 |
| I | 68 | 114 | 145 | 92 |
| IIa | 67 | 158 | 245 | 277 |
| IIx | 13 | 27 | 29 | 27 |
| Total | 148 | 299 | 419 | 396 |
|  | Disuse |  |  |  |
| Type | Dis1 | Dis2 | Dis3 | Dis4 |
| I | 145 | 205 | 116 | 224 |
| IIa | 279 | 375 | 250 | 391 |
| IIx | 35 | 98 | 32 | 122 |
| Total | 459 | 678 | 398 | 737 |
|  | Reloading |  |  |  |
| Type | Re1 | Re2 | Re3 | Re4 |
| I | 173 | 116 | 147 | 96 |
| IIa | 215 | 231 | 194 | 136 |
| IIx | 96 | 48 | 58 | 61 |
| Total | 484 | 395 | 399 | 293 |
