# Supplementary material for: Distribution of myogenic stem cell activator, hepatocyte growth factor, in skeletal muscle extracellular matrix and effect of short-term disuse and reloading
Source: PLoS One. 2025 Sep 3;20(9):e0321839. doi: 10.1371/journal.pone.0321839 (PMC12407438; doi:10.1371/journal.pone.0321839)
Supplement: Attachment — Submitted filename: PONE-D-25-12995_ReviewerNotes.pdf [file pone.0321839.s011.pdf]

Criteria	Comments
**Experiments, statistics, and other analyses performed to a high technical standard and described in sufficient detail**	**Major issues:** (1) **Very small sample size** (only 3 or 4 mice per group), (2) **Statistical approach is basic** — just t-tests without correction for multiple comparisons despite analyzing many groups/types, (3) Some **methods are described but lack critical details** (e.g., how signal intensity for nitrated HGF was quantified if at all; they call it "qualitative"), (4) **No true quantification** of some key measurements, only qualitative staining.
**Conclusions are appropriately presented and supported by the data**	They reasonably report their observations, but they **make some strong mechanistic claims** ("nitration inhibits satellite cell activation" etc.) that are **not directly proven** by their data — they would need functional assays (like satellite cell activity) to really conclude that. They even acknowledge six limitations, but the conclusions still stretch beyond their evidence.

Major Concerns

1. **Small sample sizes** severely limit the strength of conclusions.
    - Only 3 mice for HGF distribution studies and 4 mice per group for the disuse/reloading study are too few for biological experiments, especially considering fiber-type heterogeneity.
    - Recommend increasing sample size to improve statistical power.
2. **Limited and basic statistical analyses.**
    - Only simple **unpaired Student’s t-tests** are used, with no correction for multiple comparisons (despite >10 comparisons across fiber types and muscles).
    - Consider more rigorous statistical approaches (e.g., ANOVA with post hoc tests, correction for multiple comparisons such as Bonferroni or Benjamini-Hochberg).
3. **Qualitative rather than quantitative evaluation of critical endpoints.**
    - For key outcomes (like levels of nitrated HGF), no real quantification is presented — only representative images and subjective descriptions.
    - Signal intensity from immunostaining could be quantified (e.g., using ImageJ/Fiji or a similar software, standardized thresholds, and reporting mean fluorescence intensity ± SEM).
    - Without quantification, the claims about "increase" or "decrease" remain anecdotal.
4. **No functional confirmation of biological consequences.**
    - The study links HGF nitration to impaired satellite cell activation, but no direct measurement of satellite cell behavior (activation markers, proliferation rates, fusion assays) is provided.
    - Consider adding markers like Pax7, MyoD, or Ki67 staining to actually assess satellite cell status.
5. **Overinterpretation of findings.**
    - Some conclusions are speculative (e.g., mechanisms that "counteract nitration") without experimental validation.

    - It would strengthen the manuscript to more clearly separate observational findings from speculative discussion.
6. **Important experimental details missing.**
    - How were muscle fiber types randomly selected? How was consistency ensured between samples?
    - How was antibody specificity validated (particularly for anti-nitrotyrosine and anti-nitrated HGF antibodies)?
    - How was background fluorescence corrected in imaging?
7. **Unclear normalization strategy.**
    - It is not clearly stated how measurements were normalized across different animals, images, or staining batches.
    - This is important for comparability, especially in immunofluorescence studies.

Moderate Concerns

- **Muscle fiber type transitions are discussed but not tracked properly.**
  They discuss fiber type transitions during reloading but do not use fiber typing across timepoints in a rigorous, quantified way (e.g., no longitudinal tracking or lineage tracing).
- **Oxidative stress mechanisms are speculative.**
  They suggest oxidative stress during disuse causes nitration, but they only use indirect indicators (fiber atrophy and nitrotyrosine staining). Direct ROS assays or mitochondrial stress markers would strengthen the conclusions.

Summary of Key Recommendations

- Quantify immunofluorescence signal intensities.
- Use larger sample sizes.
- Apply more rigorous statistical tests.
- Include direct satellite cell functional assays (even basic ones like Pax7 or MyoD counts).
- Titrate claims carefully, separating data-based conclusions from hypotheses/speculation.
- Add missing experimental and imaging details.

Line notes

- Pg. 5 lines 2-5: Satellite cell fusion (SSC fusion)—which you pointed out—is a key mechanism. When satellite cells are activated, they proliferate and fuse with existing muscle fibers, contributing new myonuclei, which supports muscle growth by allowing increased transcriptional capacity. The sentence suggests satellite cells "cause

hypertrophy" simply by "providing new myonuclear cells," but this skips over the crucial fusion step.

- Pg. 6 line 12-13: It would be helpful to provide more information on why nNOS is being used as a proxy for HGF so the reader understands how they are connected and the spatial correlation between them.
- Pg. 7 lines 7-8: I'm not sure that using the tamoxifen example is the most effective for highlighting inactivation of SSCs, since lack of estrogen is known to have detrimental impacts on muscle that include more than just SSC inactivation.
- Pg. 9 line 3: How was 4 mice per group decided? Power analysis based on prior data?
- Pg. 9/10: How were 5 days of deloading and 3 days reloading decided? Citation?
- Pg. 13: Was t-test really the appropriate statistical method to use? Since there were three groups wouldn't an ANOVA have been more appropriate?
- Pg. 18: Why was signal intensity assessed qualitatively? It's unclear why HGF was quantitatively assessed in the first part of the study but not in the disuse section.
- Pg. 21 lines 16-18: Is that statement that oxidative stress shortened the fiber diameter really supported?
- Misc. method note: Need to justify why min fiber diameter used versus other metrics to look at overall changes (CSA, avg fiber diameter, etc.). Were other metrics collected on the fibers? Also, were 10 fibers really enough? Was it tested with more to see if it was sufficient?
- I don't think this sentence is supported by the data or prior literature "This suggests that similar to aging, the activation of satellite cells is suppressed when exercise is resumed in disuse muscle atrophy."
- Pg. 24 lines 7-9: limitation is unclear sounds more like a general statement of a scientific need.
