# Supplementary material for: Distribution of myogenic stem cell activator, hepatocyte growth factor, in skeletal muscle extracellular matrix and effect of short-term disuse and reloading
Source: PLoS One. 2025 Sep 3;20(9):e0321839. doi: 10.1371/journal.pone.0321839 (PMC12407438; doi:10.1371/journal.pone.0321839)
Supplement: Attachment — Submitted filename: report_10.1101+2025.03.14.643206.pdf [file pone.0321839.s012.pdf]

ScreenIT Report

This is an automated report for 10.1101+2025.03.14.643206.pdf. This report was solicited by the PLOS One editorial team and provided by ScreenIT.

ScreenIT is an independent group of scientists developing automated tools that analyze academic papers. A set of automated tools screened your submitted manuscript and provided the report below. Each tool was created by your academic colleagues with the goal of helping authors.

The tools look for factors that are important for transparency, rigor and reproducibility, and we hope that the report might help you to improve reporting in your manuscript. Within the report you will find links to more information about the items that the tools check. These links include helpful papers, websites, or videos that explain why the item is important.

While our screening tools aim to improve and maintain quality standards they may, on occasion, miss nuances specific to your study type or flag something incorrectly. Each tool has limitations that are described on the ScreenIT website. The tools screen the main file for the paper; they are not able to screen supplements stored in separate files.

Please note that the Academic Editor had access to these comments while making a decision on your manuscript. The Academic Editor may ask that issues flagged in this report be addressed.

If you would like to provide feedback on the ScreenIT tool, please email the team at ScreenIt@bih-charite.de. If you have questions or concerns about the review process, please contact the PLOS One office at plosone@plos.org.

How to read the report:

- no action required
- review for potential issues
- a critical issue was found by a tool
- may not be applicable to your paper

- more information about this item can be obtained using this link

Rigor

n/a	Ethics	not required.
n/a	Inclusion and Exclusion Criteria	not required.
?	Flow charts and attrition	We did not find a study flow chart of excluded observations. We strongly recommend using flow charts because they provide an overview of the study design and more information about attrition. If you included a study flow chart in your supplemental files, we apologize for missing this. Our tool is not able to screen separate supplemental files.<br><br>Sentence about attrition: *"not required."*
n/a	Sex as a biological variable	not required.
n/a	Subject Demographics	Age: not required.<br>Weight: not required.
n/a	Randomization	not required.
n/a	Blinding	not required.
n/a	Power Analysis	not required.
n/a	Replication	not required.

Generated by Barzooka (flow charts), SciScore (everything else). SciScore only screens the text of the methods section; we apologize if the tool has missed items reported elsewhere in the paper or supplements.

Resources

No key resources detected.
Generated by SciScore. SciScore only screens the text of the methods

section; we apologize if the tool has missed items reported elsewhere in the paper or supplements.

Transparency

?	Open code	ℹ If you wrote code to analyze your data, please consider sharing the code in a public repository. This makes it easier for others to reproduce your analyses, and may also aid others seeking to analyze similar datasets.
?	Open data	ℹ If permitted, sharing data on a public repository with a persistent identifier (a DOI or accession number) can improve reproducibility and make it easier for other scientists to expand on your work. Papers with open data are cited more often than papers without open data. Some institutions have an expert who can provide advice on data sharing.
✓	Self-acknowledged limitations	ℹ *"Our experiment has six limitations: (1) The study was conducted on mice, and similar reactions in humans remain unguaranteed. Using human muscle tissue samples, confirming that HGF in the ECM is abundant around type IIa and IIx muscle fibers is necessary. (2) A clear characterization of HGF abundance in the ECM is required. Increasing the myonuclear number and administering tamoxifen must clearly distinguish between type IIa, IIx, and type IIb muscle fibers. (3) We used only nitrotyrosine as an oxidative stress marker, and the generation of reactive oxygen species was indirectly indicated by the atrophy of type I muscle fibers. A more detailed evaluation would require the use of other oxidative stress markers. (4) Other more accurate methods may exist for quantifying HGF levels in the ECM surrounding each muscle fiber. A higher magnification microscope might enable us to determine which side of the ECM, labeled with the anti-laminin antibody, HGF is. (5) More quantitative methods are r..."*
✓	Conflict of interest statement	ℹ Thank you for stating any conflicts of interest.
✓	Funding statement	ℹ Thank you for reporting your funding source(s), or stating that there was no specific funding for this work.

n/a	Registration statement	ⓘ A registered protocol makes the planned research project more transparent and can protect against bias. Please consider registering future confirmatory studies.

Generated by ODDPub, limitation-recognizer, rtransparent, trial-identifier.

Figures

?	Bar graphs of continuous data	ⓘ We found bar graphs of continuous data. We recommend replacing these with more informative graphics (e.g. dotplots, box plots, violin plots), as many different datasets can lead to the same bar graph. The actual data may suggest different conclusions from the summary statistics alone. Please see the following resources for more information on the problems with bar graphs (Weissgerber et al 2015) and what to use instead (Weissgerber et al 2019).
?	Rainbow color maps	ⓘ Please consider replacing the rainbow ("jet") colormap(s) that we detected in at least one figure with alternatives like Viridis or Cividis. Rainbow color maps distort readers' perception of the data by introducing visual artifacts. Rainbow color maps are also inaccessible to readers with colorblindness. Alternatives colormaps like Viridis and Cividis are accessible to colorblind readers and avoid visual artifacts.

Generated by JetFighter, Barzooka.
