# Supplementary material for: Distribution of myogenic stem cell activator, hepatocyte growth factor, in skeletal muscle extracellular matrix and effect of short-term disuse and reloading
Source: PLoS One. 2025 Sep 3;20(9):e0321839. doi: 10.1371/journal.pone.0321839 (PMC12407438; doi:10.1371/journal.pone.0321839)
Supplement: Attachment — Submitted filename: Response to Reviewers.docx [file pone.0321839.s013.docx]

Ms.ID: PONE-D-25-12995

June 30, 2025

Dear Dr Emily Chenette

*Editor-in-Chief*, *PLoS ONE*

Dear Editor:

We acknowledge that the manuscript includes experiments that are dependent on background details (from previous reports by us and others); therefore, reviewer comments were helpful, and numerous revisions were made. In summary, to address the reviewers’ comments, we briefly explained the outline of the findings in our previous reports in the introduction. Subsequently, we increased the number of muscle fibers to be measured to 30 for each muscle fiber type and redid the measurements and analysis. We also added cross-sectional area to the measurement items. As some muscle fibers had fewer than 30 fibers, we gave up on two-way ANOVA and used Bonferroni correction to adjust for multiple comparisons. We hope the revisions and corrections adequately address the concerns of the editor and three reviewers.

Response to Reviewer #1:

Major suggestions

Reviewer #1: One major problem is that the description of image analysis is inadequate and needs to be corrected.

1. Representation of the word "tamoxifen" (p7 line6)

The wording ‘administration of tamoxifen’ does not adequately describe the content of Englund's study and should be amended as follows. Also, Englund et al. (2020) used 5-month-old mice in their experiments, so young is ambiguous and the age in months should be specified.

Englund et al. (2020) reported that young mice treated with tamoxifen to inhibit satellite cells showed slower growth in the long term [26].

->

Englund et al. (2020) reported that young (5 month old) Pax7-diphtheria toxin A (DTA) mice treated with tamoxifen to deplete satellite cells showed slower growth in the long term [26].

Similarly, the meaning of (2) administering tamoxifen in the discussion (p.24 lines 7-9) is unclear: tamoxifen is a drug that kills satellite cells in Pax7-diphtheria toxin A (DTA) mice with diphtheria toxin. It is a drug that kills satellite cells in Pax7-diphtheria toxin A (DTA) mice by diphtheria toxin and does not affect satellite cells when administered to wild-type mice. The myonuclear number does not lead to a method to distinguish between types IIa, IIx, and IIb.

Thank you for pointing that out. Following the reviewer's comments, I have revised the relevant part of the Introduction (p.8, lines 12–15) as follows:

Englund et al. (2020) reported that young (5 months old) Pax7-diphtheria toxin A (DTA) mice treated with tamoxifen to deplete satellite cells showed slower growth in the long term [27]

Next, the authors apologize for the ambiguity of the intended meaning of limitation (2) in the discussion owing to inappropriate wording. Researchers in several studies claim that muscle stem cells are not necessary for muscle hypertrophy in mature individuals, because muscle hypertrophy was observed in the experimental group of Pax7-diphtheria toxin A (DTA) mice administered tamoxifen. Most of these papers are based on hypertrophy of fast-twitch type II muscle fibers, and only a few reports distinguish type IIa and IIx muscle fibers from type IIb muscle fibers, as pointed out in this paper. The characteristics of HGF distribution in the ECM, which is a characteristic of type IIa and type IIx muscle fibers, will be clarified by evaluating the satellite cell depletion experiment after distinguishing the subtypes of type II muscle fibers. Therefore, the relevant part (p. 27, lines 6–11) has been revised as follows.

(2) The merits of HGF distribution in the ECM have not been clearly characterized. The increase in myonuclear number and trends in muscle hypertrophy should be investigated by distinguishing between subtypes of type II muscle fibers. In particular, type IIb muscle fibers may hypertrophy faster than type IIa and IIx muscle fibers even if satellite cells are depleted in Pax7-diphtheria toxin A (DTA) mice treated with tamoxifen.

2. Imaging analysis methods

The methods for calculating HGF distribution and muscle fibre diameter are unclear, with only a brief mention in the Methods (p12 lines13-16) or Results section, which is insufficient. With regard to HGF distribution, as myofibres are adjacent to each other, overlap should occur in the ECM stained with anti-laminin for each adjacent type. This should be clearly stated. In any case, an Image analysis section should be set up in Methods, which should state how the laminin staining sites were processed in the neighbouring fibre types, how the Minimal Feret diameter of the myofibres was measured (what application?), the number of myofibres analysed, the number of myofibres analysed, the number of fibres analysed, and the number of myofibres analysed. ), how many myofibres per individual were analysed, and should be detailed to the extent that other researchers can reproduce the results.

Thank you for pointing that out. The authors have added the image analysis item to the Materials and Methods section. The author also clarified that the boundary area with adjacent muscle fibers is included in both measurements. If we could distinguish the extracellular matrix from adjacent muscle fibers and extract it in a specified area, this processing would not be necessary. However, even with this method, the author was noted the difference in the muscle stem cell activation control mechanism for each muscle fiber type, as there was a significant difference in the distribution length of HGF for each muscle fiber type. In addition, because muscle fibers do not necessarily run horizontally to skeletal muscles, even if skeletal muscles are cut horizontally, the muscle fibers may be cut obliquely. In an elliptical cut surface, the short diameter is not affected by the angle of the cut surface to the muscle fibers, so this was prioritized. In response to the reviewers' comments, the number of muscle fibers measured was increased to 30, and both the minimal Feret diameter, as the short diameter, and the cross-sectional area were measured. As a result, the cross-sectional area showed more statistically significant differences. The relevant parts of the Abstract (p.3, line 14 – p.4, 1), Introduction (p.6, line 17 – p.7, line 10), Materials and Methods (p.14, line 8 – p.15, line 3), Results (p.16, lines 11–15, p.16, line 16 – p.17, line 6, p.18, lines 18 – p.19, line 3, p.19, line 3 – p.20, line 3), Discussion (p.27, lines 15–17), and Figures (p.17, lines 14–15, p.20, lines 12–13, p.39, lines 3–13, p.40, line 8 – p.41, line 2) have been revised as follows.

In Sol muscle, type IIa and IIx muscle fibers exhibited higher HGF distribution in the ECM (61.5±1.0% and 56.7±1.1%, respectively) than type I fibers (32.3±1.0%; p<0.001). In Pla and Gas muscle, type IIa (55.8±0.9% and 58.8±1.5%, respectively) and type IIx fibers (49.6±0.9% and 48.9±1.1%, respectively) had significantly higher HGF distribution in the ECM than type IIb fibers (18.6±0.9% and 13.0±1.0%; p<0.001, respectively).

We attempted to quantitatively evaluate the distribution of HGF around muscle fibers as the average signal intensity in immunofluorescence staining, but gave up owing to the difficulty of extracting the cross-sectional area of the extracellular matrix layer around a single muscle fiber as a specified region and distinguishing it from the extracellular matrix of adjacent muscle fibers. To assess the distribution of HGF in the tissue surrounding myofibers, the activation rates of neuronal nitric oxide (NO) synthase (nNOS) [20] can be used as a reference. Sarcolemmal nNOS activity was assessed using modified NADPH-diaphorase (NADPH-d) histochemistry, and the length of NADPH-d-positive sarcolemma was calculated relative to the cross-sectional circumference (CSC). Therefore, the main objective of this study was to quantitatively evaluate the distribution of HGF in the ECM surrounding different types of myofibers using this method.

Morphometric analyses

Micrographs were analyzed with morphometric measurements (ImageJ software). To measure each muscle fiber, we distinguished each muscle fiber via the ECM identified by laminin, and compared serial sections to randomly select 30 fibers for each muscle and muscle fiber type for each individual. However, certain individuals in Sol had fewer than 30 type IIx fibers; therefore, we measured the maximum number of fibers. Myofiber size was evaluated by measuring myofiber cross-sectional area (CSA), and additionally the Minimal Feret's diameter as short diameter, as its value is independent of orientation allowing obliquely oriented myofibers. Subsequently, to calculate the distribution rates of HGF in ECM around each muscle fiber type, we measured the length of BL, as the cross-sectional circumference, rebelled with anti-laminin and the length of HGF distribution in ECM, rebelled with anti-HGF antibody. The antibody-positive areas at the boundaries between the muscle fibers being measured and adjacent muscle fibers were not distinguished and were all included in the distribution length.

Thirty muscle fibers were randomly selected for each muscle fiber type from the stained images of each muscle, and the length of ECM surrounding each muscle fiber labeled with anti-laminin antibody or the HGF distribution on ECM labeled with anti-HGF antibody was measured using Image J; then, the distribution rate was calculated (n=147–180).

In Sol muscle, type IIa, IIx muscle fibers (61.5 ± 1.0%, 56.7 ± 1.1%) have higher HGF distribution in ECM than type I muscle fibers (32.3 ± 1.0%; p<0.001). In Pla muscle, IIa, IIx muscle fibers (55.8 ± 0.9%, 46.9 ± 0.9%) have higher HGF distribution in ECM than IIb muscle fibers (18.2 ± 0.9%; p<0.001), and IIa muscle fibers have higher HGF distribution in ECM than IIx muscle fibers (p=0.006). Additionally, in Gas muscle, IIa, IIx muscle fibers (58.8 ± 1.5%, 48.9 ± 1.1%) have higher HGF distribution in ECM than IIb muscle fibers (13.0 ± 1.0%; p<0.001), and IIa muscle fibers have higher HGF distribution in ECM than IIx muscle fibers (p=0.001).

Thirty muscle fibers were randomly selected for each muscle fiber type from the stained images of the Sol muscles in each group, then CSA and the Minimal Feret Diameter of each muscle fiber, as the short diameter, was measured using ImageJ (n=96–120)

Regarding CSA, type I muscle fibers were larger in the control group (1360.5 ± 43.4 µm^2^) than in the disuse (1071.7 ± 29.5 µm^2^; p<0.001) and reloading groups (958.4 ± 25.0 µm^2^; p<0.001). Type IIa muscle fibers were also larger in the control group (1058.3 ± 28.1 µm^2^) than in the disuse (949.0 ± 27.8 µm^2^; p=0.006) but did not differ from reloading groups (984.4 ± 35.7 µm^2^). Type IIx muscle fibers were larger in the disuse group (1291.3 ± 46.9 µm^2^) than in control group (1060.1 ± 38.1 µm^2^; p<0.001) and reloading groups (1051.2 ± 29.4 µm^2^; p<0.001). Regarding the Minimal Feret Diameter, type I muscle fibers were longer in the control group (34.2 ± 0.6 µm) than in the disuse (29.9 ± 0.5 µm; p<0.001) and reloading groups (29.2 ± 0.5 µm; p<0.001). Type IIa muscle fibers did not differ among the control (29.8 ± 0.5 µm), disuse (28.1 ± 0.5 µm), and reloading groups (28.7 ± 0.6 µm). Type IIx muscle fibers also had no difference among the control (29.2 ± 0.7 µm), disuse (31.5 ± 0.6 µm), and reloading groups (29.9 ± 0.5 µm) (Fig 2c). In the proportion of muscle fiber types (n=4), type I muscle fibers showed no differences among the control (35.5 ± 4.7%), disuse (30.3 ± 0.5%), and reloading groups (33.7 ± 1.7%). Type IIa muscle fibers also had no difference among control (56.6 ± 5.2%), disuse (58.0 ± 2.3%), and reloading groups (49.5 ± 3.1%) Type IIx muscle fibers were larger in the reloading group (16.8 ± 2.1%) than in the control group (7.9 ± 0.6%; p=0.006), but not in the disuse group (11.7 ± 2.3%) (Fig 2d).

A means must be established to distinguish the extracellular matrix surrounding muscle fibers from adjacent muscle fibers and extract designated regions.

[b] HGF distribution in the ECM of each muscle fiber type in the Sol, Pla, and Gas muscles (n=147–180).

[c] Cross sectional area and minimal Feret diameter of each muscle fiber in the Sol (n=96–120).

S1 Fig. Cross sectional area and minimal Feret diameter of each muscle fiber in the first experiment (n=147–180).

Regarding CSA, type IIx muscle fibers (1144.9 ± 27.7 µm^2^) were larger than type I (1036.6 ± 18.3 µm^2^; p=0.001) and IIa (966.8 ± 24.0 µm^2^; p<0.001) in Sol muscle. In Pla and Gas muscles, type IIb muscle fibers (1695.4 ± 46.6 µm^2^ and 1883.7 ± 57.3 µm^2^) were larger than type IIa (749.7 ± 21.9 µm^2^ and 702.4 ± 17.7 µm^2^; p<0.001) and IIx (1064.2 ± 25.1 µm^2^ and 888.0 ± 25.0 µm^2^; p<0.001). Regarding the Minimal Feret diameter, type IIa muscle fibers (28.3 ± 0.4 µm) are smaller than type I (29.8 ± 0.4 µm; p=0.010) and IIx (30.0 ± 0.4 µm; p=0.007), in Sol muscle. In Pla and Gas muscles, type IIb muscle fibers (39.1 ± 0.5 µm and 40.1± 0.7 µm) are larger than type IIa (23.9 ± 0.4 µm and 24.0 ± 0.4 µm; p<0.001) and IIx (29.4 ± 0.5 µm and 27.0 ± 0.4 µm; p<0.001).

S3 Fig. Cross sectional area and minimum Feret diameter of each muscle fiber in Sol (n=96–120).

Regarding CSA, type I muscle fiber (1360.5 ± 43.4 µm^2^) were lager than IIa (1058.3 ± 28.1 µm^2^; p<0.001) and IIx muscle fibers (1060.1 ± 38.1 µm^2^; p<0.001) in the control group. In the disuse group, type IIx muscle fibers (1283.0 ± 47.8 µm^2^) were larger than type I (1071.7 ± 29.5 µm^2^; p<0.001) and IIa (949.0 ± 27.8 µm^2^; p<0.001). In the reloading group, type I (958.4 ± 25.0 µm^2^), IIa (984.4 ± 35.7 µm^2^), and IIx muscle fibers (1051.2 ± 29.4 µm^2^) showed no differences in size. Regarding the minimal Feret diameter, type I muscle fiber (34.2 ± 0.6 µm) were lager than IIa (29.8 ± 0.5 µm; p<0.001) and IIx muscle fibers (29.2 ± 0.7 µm; p<0.001) in the control group. In the disuse group, type IIa muscle fibers (28.1 ± 0.5 µm) were smaller than type I (29.9 ± 0.5 µm; p=0.011) and IIx (31.5 ± 0.6 µm; p<0.001). In the reloading group, type I (29.2 ± 0.5 µm), IIa (28.7 ± 0.6 µm), and IIx muscle fibers (29.9 ± 0.5 µm) showed no differences in size.

Reviewer #2:

Major Concerns

1. Small sample sizes severely limit the strength of conclusions.

o Only 3 mice for HGF distribution studies and 4 mice per group for the disuse/reloading study are too few for biological experiments, especially considering fiber-type heterogeneity.

o Recommend increasing sample size to improve statistical power.

Thank you for pointing that out. The sample size was based on the previous study (Alaa et al., 2023) in the lab, where three mice per group were used. Because the distribution rate of HGF was a comparison between muscle fiber types, not between individual mice, we increased the number of measurement targets per muscle fiber type per muscle from 10 to 30 and redid the measurements. In addition, as suggested, we added three mice to Experiment 1. However, in Experiment 2, we determined that there would be no significant benefit in increasing the number of samples because muscle fiber atrophy and muscle fiber type transition in the disuse model are known information, and the results regarding HGF and nitroHGF amounts were qualitative evaluations as described below. The relevant sections of Materials and Methods (p.9, line 16 – p.10, line 1, p.14, lines 8–12), Results (p.15 line 18 – p.16, line 8, p.16, line 11 – p.17, line 6, p.18, line 17 – p.20, line 3), and Figures (p.17, line 9, lines 14–15, p.20, lines 12–13, p.39, lines 3–13, p.40, line 8 – p.41, line 2) have been revised and added as follows:

Six C57BL6/J mice (male, 9–11 weeks) were ordered from KBT Oriental Co Ltd, bred, and housed at 23 ± 2 °C and 55 ± 10% humidity on a 12 h light/dark cycle (lights on at 8 a.m.) with free access to regular food (CRF-1, Oriental Yeast Co., Ltd.; Tokyo, Japan) and water.

Morphometric analyses

Micrographs were analyzed with morphometric measurements (ImageJ software). To measure each muscle fiber, we distinguished each muscle fiber using the ECM identified by laminin, and compared serial sections to randomly select 30 fibers for each muscle and muscle fiber type for each individual.

The muscle fiber type proportion was obtained by quadruple immunostaining for the different MyHC isoforms (n=6). In soleus (Sol) muscle, type I muscle fibers accounted for 29.3%, type IIa muscle fibers 63.4%, and type IIx muscle fibers 7.2%. Plantaris (Pla) and gastrocnemius (Gas) muscles contained almost no type I muscle fibers, and type IIa muscle fibers were less abundant than in Sol muscle (19.8% and 6.1%). Type IIx muscle fibers were more abundant in Pla muscle (20.8%) and increased in number but only in a small proportion in the Gas muscle (6.4%). However, type IIb muscle fibers were absent in Sol muscle but accounted for 59.4% and 86.9% of Pla and Gas muscles (Fig 1a).

Thirty muscle fibers were randomly selected for each muscle fiber type from the stained images of each muscle, and the length of ECM surrounding each muscle fiber labeled with anti-laminin antibody or the HGF distribution on ECM labeled with anti-HGF antibody was measured using Image J, and the distribution rate was calculated (n=147–180). Total HGF length in the ECM was determined using an anti-HGF antibody. In Sol muscle, type IIa, IIx muscle fibers (61.5 ± 1.0%, 56.7 ± 1.1%) have higher HGF distribution in ECM than type I muscle fibers (32.3 ± 1.0%; p<0.001). In Pla muscle, IIa, IIx muscle fibers (55.8 ± 0.9%, 49.6 ± 0.9%) have higher HGF distribution in ECM than IIb muscle fibers (18.2 ± 0.9%; p<0.001), and IIa muscle fibers have higher HGF distribution in ECM than IIx muscle fibers (p=0.006). Additionally, in Gas muscle, IIa, IIx muscle fibers (58.8 ± 1.5%, 48.9 ± 1.1%) have higher HGF distribution in ECM than IIb muscle fibers (13.0 ± 1.0%; p<0.001), and IIa muscle fibers have higher HGF distribution in ECM than IIx muscle fibers (p=0.001).

Thirty muscle fibers were randomly selected for each muscle fiber type from the stained images of the Sol muscles in each group, then CSA and the Minimal Feret Diameter of each muscle fiber, as the short diameter, was measured using ImageJ (n=96–120). Regarding CSA, type I muscle fibers were larger in the control group (1360.5 ± 43.4 µm^2^) than in the disuse (1071.7 ± 29.5 µm^2^; p<0.001) and reloading groups (958.4 ± 25.0 µm^2^; p<0.001). Type IIa muscle fibers were also larger in the control group (1058.3 ± 28.1 µm2) than in the disuse (949.0 ± 27.8 µm^2^; p=0.006) but had no difference with reloading groups (984.4 ± 35.7 µm^2^). Type IIx muscle fibers were larger in the disuse group (1291.3 ± 46.9 µm^2^) than in the control group (1060.1 ± 38.1 µm^2^; p<0.001) and the reloading group (1051.2 ± 29.4 µm^2^; p<0.001). Regarding the Minimal Feret Diameter, type I muscle fibers were longer in the control group (34.2 ± 0.6 µm) than in the disuse (29.9 ± 0.5 µm; p<0.001) and reloading groups (29.2 ± 0.5 µm; p<0.001). Type IIa muscle fibers did not differ among the control (29.8 ± 0.5 µm), disuse (28.1 ± 0.5 µm), and reloading groups (28.7 ± 0.6 µm). Type IIx muscle fibers also did not differ among the control (29.2 ± 0.7 µm), disuse (31.5 ± 0.6 µm), and reloading groups (29.9 ± 0.5 µm) (Fig 2c). In the proportion of muscle fiber types (n=4), type I muscle fibers did not differ among the control (35.5 ± 4.7%), disuse (30.3 ± 0.5%), and reloading groups (33.7 ± 1.7%). Type IIa muscle fibers also did not differ among the control (56.6 ± 5.2%), disuse (58.0 ± 2.3%), and reloading groups (49.5 ± 3.1%). Type IIx muscle fibers were larger in the reloading group (16.8 ± 2.1%) than in the control group (7.9 ± 0.6%; p=0.006), but not in the disuse group (11.7 ± 2.3%) (Fig 2d).

[a] Proportion of muscle fiber types in the Sol, Pla, and Gas muscles (n=6).

[b] HGF distribution in the ECM of each muscle fiber type in the Sol, Pla, and Gas muscles (n=147–180).

[c] Cross sectional area and minimal Feret diameter of each muscle fiber in the Sol (n=96–120).

S1 Fig. Cross sectional area and Minimal Feret diameter of each muscle fiber in the first experiment (n=147–180).

Regarding CSA, type IIx muscle fibers (1144.9 ± 27.7 µm^2^) were larger than type I (1036.6 ± 18.3 µm^2^; p<0.0167) and IIa (966.8 ± 24.0 µm^2^; p<0.001) in Sol muscle. In Pla and Gas muscles, type IIb muscle fibers (1695.4 ± 46.6 µm^2^ and 1883.7 ± 57.3 µm^2^) were larger than type IIa (749.7 ± 21.9 µm^2^ and 702.4 ± 17.7 µm^2^; p<0.001) and IIx (1064.2 ± 25.1 µm^2^ and 888.0 ± 25.0 µm^2^; p<0.001). Regarding the Minimal Feret diameter, type IIa muscle fibers (28.3 ± 0.4 µm) are smaller than type I (29.8 ± 0.4 µm; p=0.010) and IIx (30.0 ± 0.4 µm; p=0.007), in Sol muscle. In Pla and Gas muscles, type IIb muscle fibers (39.1 ± 0.5 µm and 40.1± 0.7 µm) are larger than type IIa (23.9 ± 0.4 µm and 24.0 ± 0.4 µm; p<0.001) and IIx (29.4 ± 0.5 µm and 27.0 ± 0.4 µm; p<0.001).

S3 Fig. Cross sectional area and minimal Feret diameter of each muscle fiber in Sol (n= n=96–120).

Regarding CSA, type I muscle fiber (1360.5 ± 43.4 µm^2^) were lager than IIa (1058.3 ± 28.1 µm^2^; p<0.001) and IIx muscle fibers (1060.1 ± 38.1 µm^2^; p<0.001) in the control group. In the disuse group, type IIx muscle fibers (1283.0 ± 47.8 µm^2^) were larger than type I (1071.7 ± 29.5 µm^2^; p<0.001) and IIa (949.0 ± 27.8 µm^2^; p<0.001). In the reloading group, type I (958.4 ± 25.0 µm^2^), IIa (984.4 ± 35.7 µm^2^), and IIx muscle fibers (1051.2 ± 29.4 µm^2^) showed no differences in size. Regarding the minimal Feret diameter, type I muscle fiber (34.2 ± 0.6 µm) were lager than IIa (29.8 ± 0.5 µm; p<0.001) and IIx muscle fibers (29.2 ± 0.7 µm; p<0.001) in the control group. In the disuse group, type IIa muscle fibers (28.1 ± 0.5 µm) were smaller than type I (29.9 ± 0.5 µm; p=0.011) and IIx (31.5 ± 0.6 µm; p<0.001). In the reloading group, type I (29.2 ± 0.5 µm), IIa (28.7 ± 0.6 µm), and IIx muscle fibers (29.9 ± 0.5 µm) showed no differences in size.

2. Limited and basic statistical analyses.

o Only simple unpaired Student’s t-tests are used, with no correction for multiple comparisons (despite >10 comparisons across fiber types and muscles).

o Consider more rigorous statistical approaches (e.g., ANOVA with post hoc tests, correction for multiple comparisons such as Bonferroni or Benjamini-Hochberg).

Thank you for pointing that out. The number of muscle fibers measured was increased to 30 for each group, individual, and muscle fiber type. However, because Sol had fewer than 30 type IIx muscle fibers, a two-way analysis of variance could not be performed, and therefore a Bonferroni correction for multiple comparisons was added to the t-test. The relevant sections of the Abstract (p.3, lines 12–13), Materials and Methods (p.15, lines 6–14), Results (p.16, line 16 – p.17, line 6, p.19, line 3 – p.20, line 3), and Figure (p.17, line 14 – p.18, line 5, p.20, line 12 – p.21, line 1, p.39, lines 3–17, p.40, line 8 – p.41, line 2) have been revised as follows:

Student’s t-tests and the Bonferroni correction were employed for statistical analysis (p<0.05/3=0.0167).

Student’s t-tests were employed for statistical analysis of experimental results between two of the three groups and the Bonferroni correction to adjust for multiple comparisons, using Microsoft Excel X. Data are presented as mean ± standard error for 3 or 4 mice per group. We chose a significance threshold of α = 0.05/n, where n represents the number of comparisons, to account for the increased risk of false positives owing to multiple testing. This correction method helps maintain the overall Type I error rate at an acceptable level while controlling for the familywise error rate. Finally, a P-value below 0.0167 (0.05/3) was considered statistically significant and was indicated throughout by (*). Results represent independent experiments.

In Sol muscle, type IIa, IIx muscle fibers (61.5 ± 1.0%, 56.7 ± 1.1%) have higher HGF distribution in ECM than type I muscle fibers (32.3 ± 1.0%; p<0.001). In Pla muscle, IIa, IIx muscle fibers (55.8 ± 0.9%, 49.6 ± 0.9%) have higher HGF distribution in ECM than IIb muscle fibers (18.2 ± 0.9%; p<0.001), and IIa muscle fibers have higher HGF distribution in ECM than IIx muscle fibers (p=0.006). Additionally, in Gas muscle, IIa, IIx muscle fibers (58.8 ± 1.5%, 48.9 ± 1.1%) have higher HGF distribution in ECM than IIb muscle fibers (13.0 ± 1.0%; p<0.001), and IIa muscle fibers have higher HGF distribution in ECM than IIx muscle fibers (p=0.001).

Regarding CSA, type I muscle fibers were larger in the control group (1360.5 ± 43.4 µm^2^) than in the disuse (1071.7 ± 29.5 µm^2^; p<0.001) and reloading groups (958.4 ± 25.0 µm^2^; p<0.001). Type IIa muscle fibers were also larger in the control group (1058.3 ± 28.1 µm^2^) than in the disuse group (949.0 ± 27.8 µm^2^; p=0.006) but did not differ from the reloading groups (984.4 ± 35.7 µm^2^). Type IIx muscle fibers were larger in the disuse group (1291.3 ± 46.9 µm^2^) than in the control group (1060.1 ± 38.1 µm^2^; p<0.001) and reloading group (1051.2 ± 29.4 µm^2^; p<0.001). Regarding the Minimal Feret Diameter, type I muscle fibers were longer in the control group (34.2 ± 0.6 µm) than in the disuse (29.9 ± 0.5 µm; p<0.001) and reloading groups (29.2 ± 0.5 µm; p<0.001). Type IIa muscle fibers had no difference among the control (29.8 ± 0.5 µm), disuse (28.1 ± 0.5 µm), and reloading groups (28.7 ± 0.6 µm). Type IIx muscle fibers also had no difference among the control (29.2 ± 0.7 µm), disuse (31.5 ± 0.6 µm), and reloading groups (29.9 ± 0.5 µm) (Fig 2c). In the proportion of muscle fiber types (n=4), type I muscle fibers had no difference among control (35.5 ± 4.7%), disuse (30.3 ± 0.5%), and reloading groups (33.7 ± 1.7%). Type IIa muscle fibers also had no difference among control (56.6 ± 5.2%), disuse (58.0 ± 2.3%), and reloading groups (49.5 ± 3.1%). Type IIx muscle fibers were larger in the reloading group (16.8 ± 2.1%) than in the control group (7.9 ± 0.6%; p=0.006), but not in the disuse group (11.7 ± 2.3%) (Fig 2d).

[b] HGF distribution in the ECM of each muscle fiber type in the Sol, Pla, and Gas muscles (n=147–180). The length of the ECM surrounding each muscle fiber was determined using an anti-laminin antibody. Total HGF length in the ECM was determined using an anti-HGF antibody. In the Sol, IIa and IIx muscle fibers had a higher HGF distribution in the ECM than I muscle fibers (p<0.001). In the Pla and Gas muscles, IIa and IIx muscle fibers had a higher HGF distribution in the ECM than IIb muscle fibers (p<0.001).

MyHC: Myosin heavy chain, Sol: Soleus muscle, Pla: Plantaris muscle, Gas: Gastrocnemius muscle, HGF: Hepatocyte growth factor. Statistically significant difference between the two groups at p<0.0167 (0.05/3) is indicated throughout by (*).

[c] Cross sectional area and minimal Feret diameter of each muscle fiber in the Sol (n=96–120). Type I muscle fibers decreased in the disuse and reload groups compared to the control group (p<0.001).

[d] Proportions of muscle fiber types in the Sol for the control, disuse, and reloading groups (n=4). Type IIx muscle fibers increased in the reloading group compared to those in the control group (p=0.006).

BW: Body weight. CSA: Cross sectional area. Statistically significant difference between the two groups at p<0.0167 is indicated throughout by (*).

S1 Fig. Cross sectional area and minimal Feret diameter of each muscle fiber in the first experiment (n=147–180).

Regarding CSA, type IIx muscle fibers (1144.9 ± 27.7 µm^2^) were larger than type I (1036.6 ± 18.3 µm^2^; p=0.001) and IIa (966.8 ± 24.0 µm^2^; p<0.001) in Sol muscle. In Pla and Gas muscles, type IIb muscle fibers (1695.4 ± 46.6 µm^2^ and 1883.7 ± 57.3 µm^2^) were larger than type IIa (749.7 ± 21.9 µm2 and 702.4 ± 17.7 µm2; p<0.001) and IIx (1064.2 ± 25.1 µm^2^ and 888.0 ± 25.0 µm^2^; p<0.001). Regarding the Minimal Feret diameter, type IIa muscle fibers (28.3 ± 0.4 µm) are smaller than type I (29.8 ± 0.4 µm; p=0.010) and IIx (30.0 ± 0.4 µm; p=0.007), in Sol muscle. In Pla and Gas muscles, type IIb muscle fibers (39.1 ± 0.5 µm and 40.1± 0.7 µm) are larger than type IIa (23.9 ± 0.4 µm and 24.0 ± 0.4 µm; p<0.001) and IIx (29.4 ± 0.5 µm and 27.0 ± 0.4 µm; p<0.001).

MyHC, myosin heavy chain; Sol: Soleus muscle; Pla: Plantaris muscle; Gas: Gastrocnemius muscle; HGF: Hepatocyte growth factor. Statistically significant differences between the two groups at p<0.0167, as indicated by (*).

S3 Fig. Cross sectional area and Minimum Feret diameter of each muscle fiber in Sol (n=96–120).

Regarding CSA, type I muscle fiber (1360.5 ± 43.4 µm^2^) were lager than IIa (1058.3 ± 28.1 µm^2^; p<0.001) and IIx muscle fibers (1060.1 ± 38.1 µm^2^; p<0.001) in the control group. In the disuse group, type IIx muscle fibers (1283.0 ± 47.8 µm^2^) were larger than type I (1071.7 ± 29.5 µm^2^; p<0.001) and IIa (949.0 ± 27.8 µm^2^; p<0.001). In the reloading group, type I (958.4 ± 25.0 µm^2^), IIa (984.4 ± 35.7 µm^2^), and IIx muscle fibers (1051.2 ± 29.4 µm2) showed no differences in size. Regarding the minimal Feret diameter, type I muscle fiber (34.2 ± 0.6 µm) were lager than IIa (29.8 ± 0.5 µm; p<0.001) and IIx muscle fibers (29.2 ± 0.7 µm; p<0.001) in the control group. In the disuse group, type IIa muscle fibers (28.1 ± 0.5 µm) were smaller than type I (29.9 ± 0.5 µm; p=0.011) and IIx (31.5 ± 0.6 µm; p<0.001). In the reloading group, type I (29.2 ± 0.5 µm), IIa (28.7 ± 0.6 µm), and IIx muscle fibers (29.9 ± 0.5 µm) showed no differences in size.

3. Qualitative rather than quantitative evaluation of critical endpoints.

o For key outcomes (like levels of nitrated HGF), no real quantification is presented — only representative images and subjective descriptions.

o Signal intensity from immunostaining could be quantified (e.g., using ImageJ/Fiji or a similar software, standardized thresholds, and reporting mean fluorescence intensity ± SEM).

o Without quantification, the claims about "increase" or "decrease" remain anecdotal.

Thank you very much for your important comment. In past studies, even when the amount of HGF was qualitatively determined to be high, there were cases where no significant difference was observed in the mean fluorescence intensity. This may have been due to a correlation between the distribution area and amount of HGF, and we limited ourselves to a qualitative evaluation out of concern that incorrect quantification could lead to misunderstandings. The authors believe that if a method for extracting the surrounding basement membrane layer of each muscle fiber as a specified region is established, more detailed verification will be possible, and we hope that the technique will be improved in the future. The relevant sections of Introduction (p.6, line 17 – p.18, line 10), Results (p.21, lines 5–11), Discussion (p.26, lines 1–2, p.27, lines 15–17) and Figure (p.22, lines 2–11, p.41, lines 6–9, lines 13–14) have been added and revised as follows:

We attempted to quantitatively evaluate the distribution of HGF around muscle fibers as the average signal intensity in immunofluorescence staining, but gave up owing to the difficulty of extracting the cross-sectional area of the extracellular matrix layer around a single muscle fiber as a specified region and distinguishing it from the extracellular matrix of adjacent muscle fibers. To assess the distribution of HGF in the tissue surrounding myofibers, the activation rates of neuronal nitric oxide (NO) synthase (nNOS) [20] can be used as a reference. Sarcolemmal nNOS activity was assessed using modified NADPH-diaphorase (NADPH-d) histochemistry, and the length of NADPH-d-positive sarcolemma was calculated relative to the cross-sectional circumference (CSC). Therefore, the main objective of this study was to quantitatively evaluate the distribution of HGF in the ECM surrounding different types of myofibers using this method.

In the qualitative evaluation based on antibody signal intensity, the amount of nitro-HGF was more in the disuse group than that in the control group. In contrast, the amount of nitro-HGF was less in the reloading group than that in the disuse group (Fig 3a). Additionally, in the qualitative evaluation based on antibody signal intensity, the amount of HGF was more in the disuse group than in the control group and less in the reloading group than in the disuse group (Fig 3a).

In the qualitative evaluation, HGF in the disuse group were more than those in the control group.

A means must be established to distinguish the extracellular matrix surrounding muscle fibers from adjacent muscle fibers and extract designated regions.

Fig 3. Qualitative evaluation of nitration and increase in HGF levels in short-term disuse and reloading models by immunofluorescence staining.

[a] Qualitative evaluation of immunofluorescence staining with anti-HGF and anti-nitro-HGF antibodies. NitroHGF in the ECM was more in the disuse group than that in the control group and less in the reloading group than in the disuse group. In addition, HGF in the ECM was more in the disuse group than the control group and less in the reloading group than in the disuse group.

[b] Qualitative evaluation of immunofluorescence staining with anti-HGF antibody in the control group. The amount of HGF around type IIa and IIx muscle fibers in the Sol was more than that in the Pla and Gas muscles.

NitroHGF in the ECM was more in the disuse group than that in the control group and less in the reloading group than in the disuse group. In addition, HGF in the ECM was more in the disuse group than in the control group and less in the reloading group than in the disuse group.

The amount of HGF around type IIa and IIx muscle fibers in the Sol was more than that in the Pla and Gas muscles.

4. No functional confirmation of biological consequences.

o The study links HGF nitration to impaired satellite cell activation, but no direct measurement of satellite cell behavior (activation markers, proliferation rates, fusion assays) is provided.

o Consider adding markers like Pax7, MyoD, or Ki67 staining to actually assess satellite cell status.

The authors apologize for the insufficient explanation of the significance of the experimental design. We have been studying the activation of c-met receptor by HGF and the associated activation of satellite cells. In addition, in previous research conducted in our laboratory, we evaluated the poor binding of nitroHGF to c-met receptor and its inactivation in satellite cell culture systems (Alaa, et al., 2024). Therefore, this experiment focuses only on the generation of nitroHGF in the disuse model. A brief explanation of this has been added to the Introduction (p.7, line 12 – p.8, line 9).

In our past BrdU-activation assay experiment, 1-h low-frequent stretch cultures (LF: 5.0 cycles/min) activated satellite cells to a level equivalent to a positive control culture (2.5 ng/mL HGF), while a 1-h highly-frequent (HF: 8.6 cycles/min) stretch did not activate satellite cells. HGF has some tyrosine residue, which is aromatic amino acids, and two of the tyrosine residue (Y198, Y250) present on the binding surface of HGF with the receptor c-met [19]. Aromatic amino acids are easily nitrated by ONOO^-^ formed from the major messenger NO and superoxide (O_2_^-^), and nitrotyrosine is a promising biomarker of oxidative stress caused [22]. In our past experiment, conditioned media from stretch cultures were evaluated for nitration of tyrosine residues by western blotting, and nitrotyrosine was detected exclusively in 1-h HF stretch cultures. In vitro experiment of satellite cultures with nitroHGF, by acute exposure of HGF to ONOO^-^, showed a decrease in activation rate at BrdU-activation assay and receptor c-met binding activity at c-Met binding assay (ELISA-like assay). From the above, nitroHGF shows poor binding possibly due to a change in polarity, resulting in the failure to activate muscle satellite cells and repair of muscle damage sites [19, 21].

5. Overinterpretation of findings.

o Some conclusions are speculative (e.g., mechanisms that "counteract nitration") without experimental validation.

o It would strengthen the manuscript to more clearly separate observational findings from speculative discussion.

Thank you for this accurate observation. The existence of an enzyme that reduces nitration has been confirmed by members of the same laboratory, but the protein structure has not yet been identified. To protect the rights of those members, the author cannot mention it in this paper. The relevant sections of the Discussion (p.28, lines 14–16) have been revised as follows:

Satellite cells may be reactivated by a reduction mechanism for nitration during reloading, which has a rapid effect on type IIa and IIx muscle fibers in young individuals.

6. Important experimental details missing.

o How were muscle fiber types randomly selected? How was consistency ensured between samples?

o How was antibody specificity validated (particularly for anti-nitrotyrosine and anti-nitrated HGF antibodies)?

o How was background fluorescence corrected in imaging?

Thank you for your question. The muscle fibers to be measured were selected by assigning numbers to all muscle fibers after classification with Laminin, and randomly selecting them using the random number function in Excel. Antibodies that are not commonly used, such as anti-HGF antibodies and anti-nitroHGF antibodies, were used after our laboratory verified their specificity in previous studies and approved them (Alaa, et al., 2024). In addition, the background fluorescence in imaging was corrected with the threshold at which the negative control was not detected as the lower limit. Explanations regarding these have been added in the relevant sections of Materials and Methods (p.13 line 18 – p.14, line 2, p.14, lines 5–6, lines 10–13).

These antibodies have been used in previous studies and their specificity has been confirmed [19,34].

In imaging, the background fluorescence of the negative control without antibody was corrected at a threshold that did not show the fluorescence.

To measure each muscle fiber, we distinguished each muscle fiber using the ECM identified by laminin, and compared serial sections to randomly select 30 fibers for each muscle and muscle fiber type for each individual. However, certain individuals in Sol had fewer than 30 type IIx fibers; therefore, we measured the maximum number of fibers.

7. Unclear normalization strategy.

o It is not clearly stated how measurements were normalized across different animals, images, or staining batches.

o This is important for comparability, especially in immunofluorescence studies.

Thank you for your question. The authors believe that individual differences are unlikely to affect the distribution rate. Although muscle cross-sectional area and short diameter can be significantly affected by the size of the individual, no object can serve as a basis for normalization in isolated muscle tissue, and the weight of the animals in each group was limited to the average; therefore, we added this to the limitations. Then, as mentioned above, we did not use fluorescent signal intensity. Explanations regarding these have been added in the relevant sections (p.10, lines 7–9, p.28, lines 5–7).

After one week of recovery, mice were divided into 3 groups so that their mean body weights were as equal as possible: control group, disuse group, and reloading group, with four animals per group.

(7) Insufficient normalization strategy for muscle fiber size. A normalization strategy that is not affected by muscle atrophy, such as some kind of bone length ratio, should have been used to normalize for size differences between individuals.

Moderate Concerns

• Muscle fiber type transitions are discussed but not tracked properly.

They discuss fiber type transitions during reloading but do not use fiber typing across timepoints in a rigorous, quantified way (e.g., no longitudinal tracking or lineage tracing).

The authors apologize for not providing a sufficient explanation. Because the transition of muscle fiber types is not directly related to the distribution and nitration of HGF, we did not perform follow-up verification at multiple time points in this experimental system. However, to verify the difference in myonuclear addition rate and hypertrophy rate for each muscle fiber type depending on the distribution amount of muscle stem cells and HGF around the muscle fibers, it was necessary to explain that the transition of muscle fiber types during reloading after disuse was an impediment to verification. Because of this, certain descriptions in the text have been changed (p.23, line 17 – p.24 line 11).

However, in our experimental system, the size of type IIx muscle fibers in the disuse group were larger than that of the control group, and the size of type IIx muscle fibers in the reloading group was smaller than that of the disuse group. In the disuse group, type IIx muscle fibers were significantly larger than type IIa muscle fibers, but this significant difference disappeared in the reload group. Furthermore, the proportion of type IIx muscle fibers in the reloading group were significantly higher than that of the control group (S3 Fig). As reported in other experimental studies of disuse muscle atrophy and spinal cord injury models [36,40], we hypothesize that this occurs through the transition into type IIx muscle fibers from other smaller muscle fibers. This transition may complicate the evaluation of the characteristic hypertrophy for each muscle fiber type.

• Oxidative stress mechanisms are speculative.

They suggest oxidative stress during disuse causes nitration, but they only use indirect indicators (fiber atrophy and nitrotyrosine staining). Direct ROS assays or mitochondrial stress markers would strengthen the conclusions.

Thank you for the correct indication. In the field of research into disuse muscle atrophy, oxidative stress must be directly evaluated using ROS assays and mitochondrial stress markers; however, nitrotyrosine has recently become popular as a direct oxidative stress marker. Therefore, based on the common understanding that "disuse muscle atrophy is a pathology caused by mitochondrial-produced reactive oxygen species," as reported in previous studies, we attempted to use nitred tyrosine residue of HGF, as an alternative. In accordance with the above, certain parts of the text have been revised (p.24, lines 13–16).

However, type I muscle fibers were significantly smaller in the disuse group than in the control group, which we attributed to increased oxidative stress owing to the introduction of the disuse model, as reported in previous studies [28-31].

Line notes

• Pg. 5 lines 2-5: Satellite cell fusion (SSC fusion)—which you pointed out—is a key mechanism. When satellite cells are activated, they proliferate and fuse with existing muscle fibers, contributing new myonuclei, which supports muscle growth by allowing increased transcriptional capacity. The sentence suggests satellite cells "cause hypertrophy" simply by "providing new myonuclear cells," but this skips over the crucial fusion step.

Thank you for pointing that out. The authors have added the following explanation (p.5, lines 9–15).

The activated satellite cells then migrate toward the injury site and re-enter the cell cycle from resting conditions to proliferate. At this stage, committed satellite cells are called myoblasts. After the proliferative phase, myoblasts exit the cell cycle and differentiate into mature myocytes. Finally, myocytes fuse together to form multinucleated myotubes and/or fuse to damaged myofibers, providing new myonuclear. At this stage, the expression of myosin heavy chain (MyHC) and other contractile proteins begins [5,6].

• Pg. 6 line 12-13: It would be helpful to provide more information on why nNOS is being used as a proxy for HGF so the reader understands how they are connected and the spatial correlation between them.

The authors apologize for the inappropriate explanation. We meant that nNOS does not substitute for HGF, but that the HGF distribution rate was calculated based on the method for measuring the activation rate of nNOS in the sarcolemma. The explanation has been revised as follows (p.7, lines 3–10).

To assess the distribution of HGF in the tissue surrounding myofibers, the activation rates of neuronal nitric oxide (NO) synthase (nNOS) [20] can be used as a reference. Sarcolemmal nNOS activity was assessed using modified NADPH-diaphorase (NADPH-d) histochemistry, and the length of NADPH-d-positive sarcolemma was calculated relative to the cross-sectional circumference (CSC). Therefore, the main objective of this study was to quantitatively evaluate the distribution of HGF in the ECM surrounding different types of myofibers using this method.

• Pg. 7 lines 7-8: I’m not sure that using the tamoxifen example is the most effective for highlighting inactivation of SSCs, since lack of estrogen is known to have detrimental impacts on muscle that include more than just SSC inactivation.

Thank you for your comment. Age-related changes in skeletal muscles have been reported, including the slow progression of atrophy predominantly in fast-twitch fibers and the inactivation of muscle stem cells. However, because muscle stem cells are abundant around slow-twitch fibers, which are relatively unaffected by aging, we have been asked whether the inactivation of muscle stem cells is not significantly involved in age-related muscle atrophy. In response to this question, we attempted to cite the effects of administering tamoxifen to Pax7-diphtheria toxin A (DTA) mice, where muscle stem cell depletion inhibits muscle fiber hypertrophy in young to middle-aged individuals. We would have liked to consider adopting reports of muscle stem cell inactivation in animal models of other aging-related fast-twitch fiber-predominant muscle atrophy, such as nutritional disorders (diabetes, emaciation, cancer, etc.), which slowly increase oxidative stress, but we were unable to find such literature.

• Pg. 9 line 3: How was 4 mice per group decided? Power analysis based on prior data?

Thank you for your comment. As stated in Major Concern 1, previous studies in our laboratory used three mice per group (Alaa, et al., 2024), but we decided to use four mice per group to account for the possibility of death during the study. We attempted to extract designated regions to obtain the average signal intensity for each muscle fiber type by performing image processing such as increasing the laminin-stained extracellular matrix layer by several pixels inward, but this did not distinguish between adjacent muscle fibers, so we did not increase the number of individuals to perform a quantitative evaluation.

• Pg. 9/10: How were 5 days of deloading and 3 days reloading decided? Citation?

Thank you for pointing that out. In research into disuse muscle atrophy, the shortest disuse period is set at 2–7 days, with disuse periods of more than 10 days being treated as long-term disuse models. We set it at 5 days to confirm that nitration modification of HGF occurs early on. We had originally planned to use a group with a 14-day disuse period as a long-term disuse model, but this was abandoned because the increase in autofluorescence owing to cytoplasmic lipofuscin exceeded the signal brightness of the nitro-HGF antibody, making it impossible to distinguish from the cytoplasm. The reloading period was set with reference to the period it takes for muscle stem cells to form and fuse after exercise-induced activation (Dumon, et al., 2015).

• Pg. 13: Was t-test really the appropriate statistical method to use? Since there were three groups wouldn’t an ANOVA have been more appropriate?

Thank you for pointing that out. The authors added the analysis of Bonferroni correction for multiple comparisons, as stated in Major Concern 2.

• Pg. 18: Why was signal intensity assessed qualitatively? It’s unclear why HGF was quantitatively assessed in the first part of the study but not in the disuse section.

Thank you for pointing that out. As replied in Major Concerns 3, because the cross-sectional area of the basement membrane layer cannot be extracted as a specified region, in the first half, I used the ratio of the perimeter to the distribution length to describe the differences between muscle fiber types. However, because this method fails to consider the width of the basement membrane layer, I decided that it was inappropriate for comparing individuals of the same muscle fiber type, and did not use it for the quantitative evaluation in the second half.

• Pg. 21 lines 16-18: Is that statement that oxidative stress shortened the fiber diameter really supported?

As mentioned in Moderate Concerns, the authors understand that the description in this study alone does not provide support, but in the field of disuse muscle atrophy research, it has been repeatedly confirmed that "type I muscle fiber-dominant atrophy occurs in disuse models," and the collective wisdom of protein degradation pathway research has suggested that "mitochondrial-produced reactive oxygen is the cause." No supporting research has been reported to date.

• Misc. method note: Need to justify why min fiber diameter used versus other metrics to look at overall changes (CSA, avg fiber diameter, etc.). Were other metrics collected on the fibers? Also, were 10 fibers really enough? Was it tested with more to see if it was sufficient?

Thank you for pointing that out. The author added the image analysis item to the Materials and Methods. The author also clarified that the boundary area with adjacent muscle fibers is included in both measurements. If we could distinguish the extracellular matrix from adjacent muscle fibers and extract it in a specified area, this processing would not be necessary. However, even with this method, the authors noted the difference in the muscle stem cell activation control mechanism for each muscle fiber type, as there was a significant difference in the distribution length of HGF for each muscle fiber type. In addition, because muscle fibers do not necessarily run horizontally to skeletal muscles, even if skeletal muscles are cut horizontally, the muscle fibers may be cut obliquely. In an elliptical cut surface, the short diameter is not affected by the angle of the cut surface to the muscle fibers, so this was prioritized. In response to the reviewers' comments, the number of muscle fibers measured was increased to 30, and both the minimal Feret diameter, as the short diameter, and the cross-sectional area were measured. As a result, the cross-sectional area showed more statistically significant differences. The relevant parts of the Abstract (p.3, line 14 – p.4, line 1), Introduction (p.6, line 17 – p.7. line 10), Materials and Methods (p.14, line 8 – p.15, line 3), Results (p.16, lines 11–15, p.16, line 16 – p.17, line 6, p.18, line 18 – p.19, line 3, p.19, line 3 – p.20, line 3), Discussion (p.22, lines 15–18, p.24 lines 13–16, p.27, lines 15–17), and Figures (p.20, lines 12–14, p.39. lines 3–13, p.40, line 8 – p.41, line 2) have been revised as follows.

In Sol muscle, type IIa and IIx muscle fibers exhibited higher HGF distribution in the ECM (61.5±1.0% and 56.7±1.1%, respectively) than type I fibers 32.3±1.0%; p<0.001). In Pla and Gas muscle, type IIa 55.8±0.9% and 58.8±1.5%, respectively) and type IIx fibers (49.6±0.9% and 48.9±1.1%, respectively) had significantly higher HGF distribution in the ECM than type IIb fibers (18.6±0.9% and 13.0±1.0%; p<0.001, respectively).

We attempted to quantitatively evaluate the distribution of HGF around muscle fibers as the average signal intensity in immunofluorescence staining, but gave up owing to the difficulty of extracting the cross-sectional area of the extracellular matrix layer around a single muscle fiber as a specified region and distinguishing it from the extracellular matrix of adjacent muscle fibers. To assess the distribution of HGF in the tissue surrounding myofibers, the activation rates of neuronal nitric oxide (NO) synthase (nNOS) [20] can be used as a reference. Sarcolemmal nNOS activity was assessed using modified NADPH-diaphorase (NADPH-d) histochemistry, and the length of NADPH-d-positive sarcolemma was calculated relative to the cross-sectional circumference (CSC). Therefore, the main objective of this study was to quantitatively evaluate the distribution of HGF in the ECM surrounding different types of myofibers using this method.

Morphometric analyses

Micrographs were analyzed with morphometric measurements (ImageJ software). To measure each muscle fiber, we distinguished each muscle fiber by the ECM identified by laminin, and compared serial sections to randomly select 30 fibers for each muscle and muscle fiber type for each individual. However, certain individuals in Sol had fewer than 30 type IIx fibers, so we measured the maximum number of fibers. Myofiber size was evaluated by measuring myofiber cross-sectional area (CSA), and additionally the Minimal Feret's diameter as short diameter, as its value is independent of orientation allowing obliquely oriented myofibers. Subsequently, to calculate the distribution rates of HGF in ECM around each muscle fiber types, we measured the length of BL, as the cross-sectional circumference, rebelled with anti-laminin and the length of HGF distribution in ECM, rebelled with anti-HGF antibody. The antibody-positive areas at the boundaries between the muscle fibers being measured and adjacent muscle fibers were not distinguished and were all included in the distribution length.

Thirty muscle fibers were randomly selected for each muscle fiber type from the stained images of each muscle, and the length of ECM surrounding each muscle fiber labeled with anti-laminin antibody or the HGF distribution on ECM labeled with anti-HGF antibody was measured using Image J, and the distribution rate was calculated (n=147–180).

In Sol muscle, type IIa, IIx muscle fibers (61.5 ± 1.0%, 56.7 ± 1.1%) have higher HGF distribution in ECM than type I muscle fibers (32.3 ± 1.0%; p<0.001). In Pla muscle, IIa, IIx muscle fibers (55.8 ± 0.9%, 49.6 ± 0.9%) have higher HGF distribution in ECM than IIb muscle fibers (18.2 ± 0.9%; p<0.001), and IIa muscle fibers have higher HGF distribution in ECM than IIx muscle fibers (p=0.006). Additionally, in Gas muscle, IIa, IIx muscle fibers (58.8 ± 1.5%, 48.9 ± 1.1%) have higher HGF distribution in ECM than IIb muscle fibers (13.0 ± 1.0%; p<0.001), and IIa muscle fibers have higher HGF distribution in ECM than IIx muscle fibers (p=0.001).

Thirty muscle fibers were randomly selected for each muscle fiber type from the stained images of the Sol muscles in each group, then CSA and the Minimal Feret Diameter of each muscle fiber, as the short diameter, was measured using ImageJ (n=96–120)

Regarding CSA, type I muscle fibers were larger in the control group (1360.5 ± 43.4 µm^2^) than in the disuse (1071.7 ± 29.5 µm^2^; p<0.001) and reloading groups (958.4 ± 25.0 µm^2^; p<0.001). Type IIa muscle fibers were also larger in the control group (1058.3 ± 28.1 µm^2^) than in the disuse (949.0 ± 27.8 µm^2^; p=0.006) but did not differ from the reloading groups (984.4 ± 35.7 µm^2^). Type IIx muscle fibers were larger in the disuse group (1291.3 ± 46.9 µm^2^) than in the control group (1060.1 ± 38.1 µm2; p<0.001) and the reloading group (1051.2 ± 29.4 µm^2^; p<0.001). Regarding the Minimal Feret Diameter, type I muscle fibers were longer in the control group (34.2 ± 0.6 µm) than in the disuse (29.9 ± 0.5 µm; p<0.001) and reloading groups (29.2 ± 0.5 µm; p<0.001). Type IIa muscle fibers had no difference among the control (29.8 ± 0.5 µm), disuse (28.1 ± 0.5 µm), and reloading groups (28.7 ± 0.6 µm). Type IIx muscle fibers also had no difference among the control (29.2 ± 0.7 µm), disuse (31.5 ± 0.6 µm), and reloading groups (29.9 ± 0.5 µm) (Fig 2c). In the proportion of muscle fiber types (n=4), type I muscle fibers had no difference among control (35.5 ± 4.7%), disuse (30.3 ± 0.5%), and reloading groups (33.7 ± 1.7%). Type IIa muscle fibers also had no difference among control (56.6 ± 5.2%), disuse (58.0 ± 2.3%), and reloading groups (49.5 ± 3.1%). Type IIx muscle fibers were larger in the reloading group (16.8 ± 2.1%) than in the control group (7.9 ± 0.6%; p<0.0167), but not in the disuse group (11.7 ± 2.3%) (Fig 2d).

This may be because type I muscle fibers, around which satellite cells are abundant [7,8], are more prevalent in slow-twitch muscles, whereas type IIb muscle fibers are the largest and most abundant in fast-twitch muscles (S1 and S2 Figs)

However, type I muscle fibers were significantly smaller in the disuse group than in the control group, which we attributed to increased oxidative stress owing to the introduction of the disuse model, as reported in previous studies [28-31].

A means must be established to distinguish the extracellular matrix surrounding muscle fibers from adjacent muscle fibers and extract designated regions.

[c] Cross sectional area and minimal Feret diameter of each muscle fiber in the Sol (n=96–120). Type I muscle fibers decreased in the disuse and reload groups compared to the control group (p<0.001).

S1 Fig. Cross sectional area and minimal Feret diameter of each muscle fiber in the first experiment (n=147–180).

Regarding CSA, type IIx muscle fibers (1144.9 ± 27.7 µm^2^) were larger than type I (1036.6 ± 18.3 µm^2^; p=0.001) and IIa (966.8 ± 24.0 µm^2^; p<0.001) in Sol muscle. In Pla and Gas muscles, type IIb muscle fibers (1695.4 ± 46.6 µm^2^ and 1883.7 ± 57.3 µm^2^) were larger than type IIa (749.7 ± 21.9 µm^2^ and 702.4 ± 17.7 µm^2^; p<0.001) and IIx (1064.2 ± 25.1 µm^2^ and 888.0 ± 25.0 µm^2^; p<0.001). Regarding the Minimal Feret diameter, type IIa muscle fibers (28.3 ± 0.4 µm) are smaller than type I (29.8 ± 0.4 µm; p=0.010) and IIx (30.0 ± 0.4 µm; p=0.007), in Sol muscle. In Pla and Gas muscles, type IIb muscle fibers (39.1 ± 0.5 µm and 40.1± 0.7 µm) are larger than type IIa (23.9 ± 0.4 µm and 24.0 ± 0.4 µm; p<0.001) and IIx (29.4 ± 0.5 µm and 27.0 ± 0.4 µm; p<0.001).

S3 Fig. Cross sectional area and minimum Feret diameter of each muscle fiber in Sol (n=96–120).

Regarding CSA, type I muscle fiber (1360.5 ± 43.4 µm^2^) were lager than IIa (1058.3 ± 28.1 µm^2^; p<0.001) and IIx muscle fibers (1060.1 ± 38.1 µm^2^; p<0.001) in the control group. In the disuse group, type IIx muscle fibers (1283.0 ± 47.8 µm^2^) were larger than type I (1071.7 ± 29.5 µm^2^; p<0.001) and IIa (949.0 ± 27.8 µm^2^; p<0.001). In the reloading group, type I (958.4 ± 25.0 µm^2^), IIa (984.4 ± 35.7 µm^2^), and IIx muscle fibers (1051.2 ± 29.4 µm^2^) showed no differences in size. Regarding the minimal Feret diameter, type I muscle fiber (34.2 ± 0.6 µm) were lager than IIa (29.8 ± 0.5 µm; p<0.001) and IIx muscle fibers (29.2 ± 0.7 µm; p<0.001) in the control group. In the disuse group, type IIa muscle fibers (28.1 ± 0.5 µm) were smaller than type I (29.9 ± 0.5 µm; p=0.011) and IIx (31.5 ± 0.6 µm; p<0.001). In the reloading group, type I (29.2 ± 0.5 µm), IIa (28.7 ± 0.6 µm), and IIx muscle fibers (29.9 ± 0.5 µm) showed no differences in size.

• I don’t think this sentence is supported by the data or prior literature “This suggests that similar to aging, the activation of satellite cells is suppressed when exercise is resumed in disuse muscle atrophy.”

Thank you for pointing that out. There was an error in the text, so author have corrected it (p.25, lines 1–4).

This result suggests that similar to aging, the activation of satellite cells is suppressed in disuse muscle atrophy. In contrast, HGF and nitroHGF was less in the reloading group, so the release of HGF from the ECM did not appear to be inhibited.

• Pg. 24 lines 7-9: limitation is unclear sounds more like a general statement of a scientific need.

Thank you for pointing that out. The authors have modified and added a note regarding the limitations of the measurement method (p.27, line 3, lines 6–11, lines 15–17, p.28, lines 5–7).

Our experiment has seven limitations: (1) The study was conducted on mice, and similar reactions in humans are not guaranteed. Using human muscle tissue samples, the authors confirmed that HGF in the ECM is abundant around type IIa and IIx muscle fibers is necessary. (2) The merits of HGF distribution in the ECM have not been clearly characterized. The increase in myonuclear number and trends in muscle hypertrophy should be investigated by distinguishing between subtypes of type II muscle fibers. In particular, type IIb muscle fibers may hypertrophy faster than type IIa and IIx muscle fibers even if satellite cells are depleted in Pax7-diphtheria toxin A (DTA) mice treated with tamoxifen. (3) We used only nitrotyrosine as an oxidative stress marker, and the generation of reactive oxygen species was indirectly indicated by the atrophy of type I muscle fibers. A more detailed evaluation would require the use of other oxidative stress markers. (4) Other more accurate methods may exist for quantifying HGF levels in the ECM surrounding each muscle fiber. A means must be established to distinguish the extracellular matrix surrounding muscle fibers from adjacent muscle fibers and extract designated regions. (5) More quantitative methods are required to determine the degree of nitro-HGF. HGF is present in extremely low amounts in the skeletal muscle tissue, making it difficult to quantify by western blotting. However, a quantitative evaluation is necessary for a clear evaluation. (6) More detailed verification, such as reverse transcription polymerase chain reaction (PCR) is required regarding the increased expression of HGF in response to oxidative stress and the conditions for muscle fiber-type transition. (7) Insufficient normalization strategy for muscle fiber size. A normalization strategy that is not affected by muscle atrophy, such as some kind of bone length ratio, should have been used to normalize for size differences between individuals.

Reviewer #3: This is an automated report for PONE-D-25-12995. This report was solicited by the PLOS One editorial team and provided by ScreenIT.

ScreenIT is an independent group of scientists developing automated tools that analyze academic papers. A set of automated tools screened your submitted manuscript and provided the report below. Each tool was created by your academic colleagues with the goal of helping authors. The tools look for factors that are important for transparency, rigor and reproducibility, and we hope that the report might help you to improve reporting in your manuscript. Within the report you will find links to more information about the items that the tools check. These links include helpful papers, websites, or videos that explain why the item is important. While our screening tools aim to improve and maintain quality standards they may, on occasion, miss nuances specific to your study type or flag something incorrectly. Each tool has limitations that are described on the ScreenIT website. The tools screen the main file for the paper; they are not able to screen supplements stored in separate files. Please note that the Academic Editor had access to these comments while making a decision on your manuscript.

Thank you for your comment. Your report on our manuscript did not include any items that were found to be "a critical issue was found by a tool." Regarding the five items that were found to be "review for potential issues," we would like to respond as follows.

Rigor

Flow charts and attrition

We did not find a study flow chart of excluded observations. We strongly recommend using flow charts because they provide an overview of the study design and more information about attrition. If you included a study flow chart in your supplemental files, we apologize for missing this. Our tool is not able to screen separate supplemental files.

Thank you for your comment. This study consists of two experimental groups, each dealing with a different topic. For this reason, we have determined that a flowchart for gradually narrowing the focus is not necessary.

Transparency

Open code

If you wrote code to analyze your data, please consider sharing the code in a public repository. This makes it easier for others to reproduce your analyses, and may also aid others seeking to analyze similar datasets.

Thank you for your comment. We did not use any proprietary code for our analysis and do not have any code that needs to be made public.

Open data

If permitted, sharing data on a public repository with a persistent identifier (a DOI or accession number) can improve reproducibility and make it easier for other scientists to expand on your work. Papers with open data are cited more often than papers without open data. Some institutions have an expert who can provide advice on data sharing.

Thank you for your comment. We have published the measured values of the analyzed data as a Supporting Table. All three reviewers have answered "Yes" to the question "3. Have the authors made all data underlying the findings in their manuscript fully available?" in the "Reviewer's Responses to Questions - Comments to the Author" section.

Figures

Bar graphs of continuous data

We found bar graphs of continuous data. We recommend replacing these with more informative graphics (e.g. dotplots, box plots, violin plots), as many different datasets can lead to the same bar graph. The actual data may suggest different conclusions from the summary statistics alone. Please see the following resources for more information on the problems with bar graphs (Weis sgerber et al 2015) and what to use instead (Weissg erber et al 2019).

Thank you for pointing that out. As per your instructions, I changed the graphs with fewer data to dot plots and the graphs with more data to box plots.

Rainbow color maps

Please consider replacing the rainbow (“jet”) colormap(s) that we detected in at least one figure with alternatives like Viridis or Cividis. Rainbow color maps distort readers’ perception of the data by introducing visual artifacts. Rainbow color maps are also inaccessible to readers with colorblindness. Alternatives colormaps like Viridis and Cividis are accessible to colorblind readers and avoid visual

artifacts.

Thank you for your comment. The author presumes that "the rainbow colormap" you pointed out refers to a multi-stained image in direct-immunofluorescence microscopy. Since it is consistent with the staining in previous studies (Alaa, et al., 2024) and is not a color map, we have not made any corrections this time. However, if this stained image is difficult for readers with color vision deficiencies to see, we would appreciate it if you could point it out again.
